# Navigating Transition Metal‐Dependent Cell Death: Mechanisms, Crosstalk, and Future Directions

**DOI:** 10.1002/advs.202501974

**Published:** 2025-11-03

**Authors:** Qinghang Song, Yuxuan Yang, Lina Yang

**Affiliations:** ^1^ School of Basic Medicine Qingdao University Qingdao 266071 China; ^2^ Department of Oncology The Affiliated Yantai Yuhuangding Hospital Qingdao University Yantai 264000 China

**Keywords:** advanced technology, cobaltosis, cuproptosis, ferroptosis, metal death code, regulated cell death, transition metal

## Abstract

Regulated cell death is fundamental to cellular homeostasis, with metal‐dependent pathways emerging as critical mechanisms influencing cell fate decisions. This comprehensive review examines the emerging landscape of metal‐dependent regulated cell death pathways, focusing on ferroptosis, cuproptosis, and the hypothetical concept of “cobaltosis.” Ferroptosis, characterized by iron‐catalyzed lipid peroxidation and glutathione depletion, has emerged as a critical pathway in cancer, neurodegeneration, and ischemia‐reperfusion injury. Cuproptosis, a recently defined copper‐induced death mechanism, operates through direct binding of copper to lipoylated mitochondrial proteins, causing proteotoxic stress and metabolic collapse. Evidence for cobaltosis is critically evaluated as a potential distinct regulated cell death pathway, cobalt's known cellular impacts are examined, and a rigorous experimental framework is proposed for its investigation and validation as a distinct entity. This review comparatively analyzes the core mechanisms, highlighting the distinct roles of reactive oxygen species and mitochondria in each pathway. The intricate crosstalk and divergence among these death modalities are explored, introducing the concept of a “metal death code” that dictates the cellular fate in response to specific metal stresses. Furthermore, advanced technologies for detecting and dissecting these pathways are discussed, and their significant translational potential for treating diseases characterized by metal dyshomeostasis is considered, such as cancer and neurodegeneration.

## Graphical abstract

Transition metals trigger distinct regulated cell death pathways beyond traditional apoptosis. This review examines ferroptosis (iron‐catalyzed lipid peroxidation) and cuproptosis (copper‐induced mitochondrial proteotoxicity), while exploring hypothetical “cobaltosis” as a novel pathway. Advanced detection methods, therapeutic applications, and the concept of a “metal death code” governing cellular fate decisions are discussed, offering new perspectives for precision medicine targeting metal dyshomeostasis.

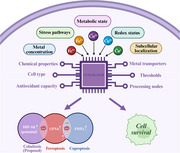

## 1 Introduction

### 1.1 Overview of Regulated Cell Death (RCD)

Cell death is a natural process in which cells permanently cease to perform their essential functions, resulting in the breakdown of their structure, function, and biochemical components.^[^ ^1^ ^]^ The balance between cell survival and death is crucial for maintaining organismal homeostasis.^[^ ^2^, ^3^ ^]^ To better understand and classify cell death, the Nomenclature Committee on Cell Death (NCCD) has defined the major forms of cell death on the basis of genetics, biochemistry, pharmacology, and function, rather than morphology, and established criteria for identifying dead cells through irreversible plasma membrane permeability or complete disintegration.^[^ ^4^, ^5^, ^6^, ^7^, ^8^ ^]^ RCD is often synonymous with caspase‐dependent apoptosis, but it also includes several non‐apoptotic forms, such as necroptosis, pyroptosis, ferroptosis (derived from the Greek “ptosis,” meaning “falling,” and the Latin “ferrum,” meaning “iron”), entosis, autophagy‐dependent cell death, and alkaliptosis, among others. These forms of cell death differ in their biochemical, functional, and morphological characteristics. The elucidation of these diverse RCD pathways has revealed an intricate cellular decision‐making process, in which the choice of death modality is influenced by the nature and intensity of the stress stimulus, the cellular metabolic state, the availability of specific pathway components, and extensive crosstalk between different RCD signaling cascades.^[^ ^3^ ^]^ Understanding this complex landscape is paramount, as it not only deepens our understanding of fundamental biology but also offers novel therapeutic avenues for diseases characterized by aberrant cell death.

### 1.2 Biological Roles of Transition Metals

Metals are vital for life as they are required for fundamental biological processes. Redox‐inert metals, such as calcium, potassium, sodium, zinc, cadmium, and lead,^[^ ^9^ ^]^ function mainly through fluctuations in their ionic pools to signal cellular responses. In contrast, redox‐active transition metals, like copper and iron, drive catalytic activity and are generally described as static cofactors.^[^ ^10^ ^]^ These cofactors are tightly sequestered within protein active sites to prevent harmful reactive oxygen species (ROS) production through Fenton chemistry. Copper's role has expanded from a catalytic cofactor to a dynamic signaling molecule that influences both normal physiology and disease states.^[^ ^11^ ^]^

Transition metals, a group of elements characterized by their partially filled d orbitals, are indispensable micronutrients for virtually all forms of life. Their unique chemical properties, particularly their ability to exist in multiple oxidation states and form complex coordination compounds, allow them to participate in a vast array of fundamental biological processes. They serve as essential structural components of numerous proteins, ensuring their correct folding and stability. More prominently, they act as catalytic cofactors for a myriad of enzymes involved in vital biochemical reactions, including those central to cellular respiration,^[^ ^12^, ^13^ ^]^ DNA synthesis and repair,^[^ ^14^, ^15^ ^]^ antioxidant defense,^[^ ^16^ ^]^ ROS metabolism,^[^ ^17^, ^18^, ^19^ ^]^ and oxygen transport.^[^ ^20^ ^]^ Transition metals also form stable complexes with ligands, allowing their participation in various biological processes. For instance, cobalt is the central atom in vitamin B12, which is essential for metabolism in human cells.^[^ ^21^, ^22^ ^]^

Given their essentiality and potential toxicity, the homeostasis of transition metals is exquisitely regulated within organisms and individual cells through sophisticated networks of transporters, chaperones, storage proteins, and efflux systems, ensuring that cells maintain an adequate supply of these metals for physiological functions while preventing their accumulation to toxic levels.^[^ ^23^, ^24^ ^]^

### 1.3 Emergence of Metal‐Dependent Cell Death Pathways

Building upon the expanded understanding of RCD pathways and the evolving appreciation of transition metals as active modulators of cell fate, the concept of metal‐dependent regulated cell death has emerged as a significant and rapidly advancing frontier in cell biology. This field recognizes that the dysregulation of specific metal ions can trigger not just non‐specific toxicity leading to accidental cell death, but rather distinct, genetically programmed, and often targetable RCD pathways.

Previous reviews have summarized the characteristics, mechanisms, and regulatory factors of ferroptosis and cuproptosis.^[^ ^25^, ^26^, ^27^, ^28^, ^29^, ^30^ ^]^ These pathways possess distinct molecular mechanisms, unique regulatory networks, and specific morphological and biochemical hallmarks that clearly differentiate them from each other and from other classical RCDs like apoptosis or necroptosis. Their increasingly recognized involvement in a wide array of physiological processes and, more prominently, in the pathogenesis of numerous human diseases.

There are many new discoveries in 2025. Calcium‐induced cell death has taken on new dimensions with the discovery of CaToptosis, a regulated pathway mediated by Tousled‐like kinase 2 (TLK2) that specifically targets postmitotic neurons.^[^ ^31^ ^]^ The so‐called “Znproptosis” involves a combination of zinc overload and ROS generation that disrupts the mitochondrial electron transport chain. The pathway specifically targets the FDX2/LIAS axis, which is similar to cuproptosis, leading to degradation of iron‐sulfur cluster proteins and proteotoxic stress.^[^ ^32^ ^]^ Necrosis by Sodium Overload (NECSO) represents the first characterized sodium‐dependent RCD pathway, focusing on TRPM4 channel activation by the compound Necrocide‐1, leading to persistent sodium influx, membrane depolarization, and osmotic cell death.^[^ ^33^ ^]^ The growing list of metal‐ and ion‐implicated RCDs further emphasizes that cellular life maintains a complex and often precarious relationship with a wide range of inorganic ions. Each metal, owing to its unique chemical properties, can exploit specific cellular vulnerabilities when its homeostasis is disrupted.^[^ ^34^ ^]^ This review will delve deeply into the process of discovery and established mechanisms of ferroptosis and cuproptosis, and will then critically examine the concept of a hypothetical cobalt‐dependent RCD, termed “cobaltosis,” by evaluating the existing knowledge of cobalt biology and proposing a framework for investigating whether cobalt can indeed trigger a unique, regulated cell death program. Understanding these specific metal‐dependent pathways not only provides fundamental insights into cellular physiology but also opens novel therapeutic avenues for diseases characterized by metal dysregulation, by offering opportunities to selectively induce or inhibit these death programs.

Our focus is on connecting the conceptual understanding with the methodological and technological advances required for their investigation. We will begin by summarizing the established biology of ferroptosis and cuproptosis. From there, we will pivot to the frontiers of the field, using the hypothetical ““cobaltosis”” to illustrate the process of scientific inquiry into a potential new RCD. This conceptual exploration naturally leads to a dedicated discussion of the advanced tools that enable such discoveries. Moreover, we will synthesize these threads by exploring the “metal death code” that dictates cellular decisions and the resulting translational opportunities that drive this research.

## 2 Ferroptosis and Cuproptosis: A Focused Overview of Established Metal‐Dependent RCDs

### 2.1 Ferroptosis: An Iron‐Dependent Cascade of Lethal Lipid Peroxidation

#### 2.1.1 Discovery and Core Molecular Mechanisms

The term “ferroptosis” was coined by Stockwell and colleagues in 2012 following their investigation into the mechanisms of certain small molecules that induced non‐apoptotic cell death in cancer cells. The discovery of ferroptosis emerged from efforts to understand the cytotoxic effects of a compound named erastin, which was identified through a synthetic lethal screening aimed at finding molecules that selectively kill cancer cells harboring oncogenic RAS mutations.^[^ ^35^ ^]^ Erastin was observed to induce a form of cell death that did not exhibit classical features of apoptosis, such as caspase activation, DNA fragmentation, or membrane blebbing. Instead, affected cells displayed necrosis‐like morphological changes, including cell rounding, swelling of mitochondria with reduced or absent cristae, and rupture of the outer mitochondrial membrane, without significant nuclear alterations. Furthermore, lipophilic antioxidants (α‐tocopherol, butylated hydroxytoluene, and β‐carotene) are potent inhibitors of erastin‐induced cell death, suggesting that ROS (possibly lipophilic) are involved in this cell death process.^[^ ^36^, ^37^ ^]^ Additionally, iron chelators were identified as inhibitors of cell death induced after RAS‐selective lethal compound 3 (RSL3) treatment, revealing the cellular requirement for iron.^[^ ^38^ ^]^

Through modulatory profiling, an unbiased pharmacological and genetic analysis system that classifies lethal compounds based on functional characteristics, erastin and RSL3 were found to cluster together, indicating that they share a similar mechanism of cell death.^[^ ^39^, ^40^ ^]^ This erastin‐RSL3 cluster is distinct from other lethal compounds that induce apoptosis and necrosis.

Further investigations revealed that erastin inhibits the cystine/glutamate antiporter system Xc^−^, leading to depletion of intracellular cysteine, a precursor for glutathione (GSH) synthesis.^[^ ^41^ ^]^ The reduction in GSH levels impairs the activity of glutathione peroxidase 4 (GPX4), a key enzyme that reduces lipid hydroperoxides to non‐toxic lipid alcohols.^[^ ^42^ ^]^ The inactivation of GPX4 results in the accumulation of lipid hydroperoxides, particularly within phospholipids containing polyunsaturated fatty acid (PUFA) chains, ultimately causing oxidative damage to cell membranes and inducing ferroptosis.^[^ ^43^ ^]^

The iron dependency of this cell death process was established when iron chelators such as deferoxamine were found to rescue cells from erastin‐induced death, whereas supplementation with iron exacerbated cytotoxicity. This indicated that iron plays a crucial role in lipid peroxidation during ferroptosis, likely through the Fenton reaction, where Fe^2^⁺ reacts with hydrogen peroxide to generate highly reactive hydroxyl radicals.^[^ ^36^, ^38^ ^]^ These radicals can abstract hydrogen atoms from PUFAs, initiating lipid peroxidation chain reactions.

Genetic studies have further elucidated the regulatory network of ferroptosis. The oncogenic RSL3 was identified as a direct inhibitor of GPX4, suggesting that GPX4 is a central regulator of ferroptosis.^[^ ^42^ ^]^ Additionally, acyl‐CoA synthetase long‐chain family member 4 (ACSL4) was found to be essential for ferroptosis by facilitating the incorporation of PUFAs into membrane phospholipids, thereby providing substrates for peroxidation.^[^ ^44^ ^]^

Additionally, the FSP1/CoQ10/NAD(P)H axis provides a parallel, GSH‐independent mechanism of ferroptosis suppression. Ferroptosis Suppressor Protein 1 (FSP1), is an NAD(P)H‐dependent oxidoreductase that reduces coenzyme Q10 (CoQ10, ubiquinone) to its reduced antioxidant form, ubiquinol (CoQH_2_).^[^ ^45^ ^]^ And, the GTP cyclohydrolase‐1 (GCH1)/tetrahydrobiopterin (BH4)/dihydrofolate reductase (DHFR) axis constitutes another recently identified endogenous antioxidant system that protects against ferroptosis. Furthermore, BH4 is required for the synthesis of CoQ10, linking this pathway to the FSP1 axis. DHFR is involved in regenerating BH4 from its oxidized forms, thus maintaining its antioxidant capacity.^[^ ^46^ ^]^

Two groundbreaking studies have definitively established NADPH‐cytochrome P450 reductase (POR) as the critical oxidant‐generating enzyme that executes ferroptosis. The mechanistic clarity is remarkable: NADPH flows through POR's FAD and FMN cofactors to reduce oxygen to H_2_O_2_, which then generates hydroxyl radicals in the presence of Fe^2^⁺. These radicals abstract hydrogen from polyunsaturated fatty acids, initiating the lipid peroxidation cascade that ultimately ruptures cellular membranes.^[^ ^47^, ^48^ ^]^

**Figure 1:**
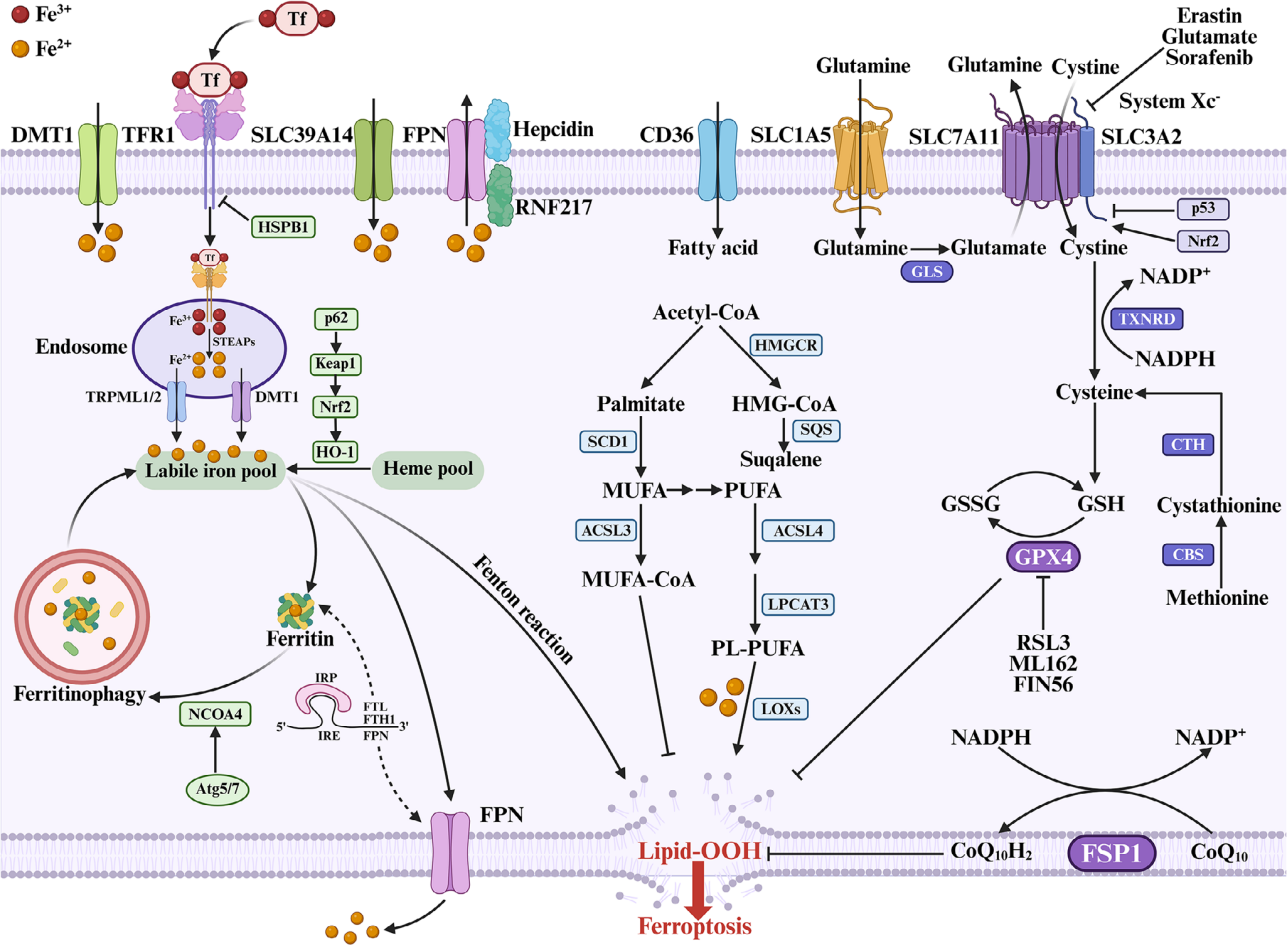
Molecular mechanisms of ferroptosis. The mechanism of ferroptosis involves three main pathways, including iron metabolism, lipid peroxidation, and antioxidant defense systems. Iron metabolism pathway: Transferrin‐bound Fe^3+^ enters cells via TFR1‐mediated endocytosis. In endosomes, STEAP proteins reduce Fe^3+^ to Fe^2+^, which is transported into the labile iron pool via DMT1 and TRPML1/2. Iron can be sequestered by ferritin or exported via FPN. The labile iron pool (Fe^2+^) participates in the Fenton reaction to induce Lipid‐OOH. The IRP proteins regulate cellular iron homeostasis by binding to IREs on target mRNAs, including FTL, FTH1, and FPN. The HSPB1‐mediated pathway and p62/Keap1/Nrf2/HO‐1 signaling cascade modulate iron homeostasis and heme metabolism. Ferritinophagy, mediated by NCOA4 and autophagy‐related proteins Atg5 and Atg7, releases iron from ferritin stores. Lipid peroxidation pathway: ACSL4 and LPCAT3 catalyze the incorporation of PUFA into PL‐PUFA. LOXs oxidize PL‐PUFAs, generating Lipid‐OOH that drive ferroptosis. SCD1 converts palmitate to MUFAs, providing a protective mechanism. Antioxidant defense systems: System Xc^−^ (composed of SLC7A11 and SLC3A2) imports cystine in exchange for glutamate. Intracellular cystine is reduced to cysteine, which is a substrate for GSH synthesis. GSH is essential for GPX4 function, which reduces lipid peroxides to lipid alcohols. The transsulfuration pathway, involving CBS and CTH, provides an alternative cysteine source from methionine. Additional antioxidant systems include FSP1‐mediated reduction of CoQ_10_ to CoQ_10_H_2_ at the plasma membrane, and the NADPH‐dependent pathways involving TXNRD. Key regulatory proteins, including p53 and Nrf2, inhibit and activate System Xc^−^, respectively. Pharmacological modulators such as erastin, glutamate, sorafenib (system Xc^−^ inhibitors), RSL3, ML162, and FIN56 (GPX4 inhibitors) can induce ferroptosis by disrupting these protective pathways.

The core molecular mechanisms of ferroptosis, largely established through in vitro studies using specific cell lines and chemical modulators, with subsequent validation and contextualization in various animal models, are graphically summarized in **Figure** **1**.

#### 2.1.2 Iron Metabolism and Homeostasis

Iron metabolism plays a pivotal role in ferroptosis, as the accumulation of intracellular iron is a key driver of this unique form of RCD.^[^ ^49^ ^]^ Excess free iron can catalyze the formation of ROS through Fenton chemistry, leading to oxidative damage of cellular components.^[^ ^50^, ^51^ ^]^ The delicate balance of iron homeostasis is maintained through tightly regulated mechanisms involving iron uptake, storage, utilization, and export.^[^ ^52^, ^53^ ^]^

Transferrin‐bound iron circulates in the bloodstream and delivers iron to cells throughout the body.^[^ ^54^ ^]^ Cells acquire iron primarily through the transferrin receptor 1 (TfR1), which binds diferric transferrin (holo‐transferrin) with high affinity.^[^ ^55^ ^]^ The Tf‐TfR1 complex undergoes clathrin‐mediated endocytosis, forming an endosome where the acidic environment promotes the release of Fe^3^⁺ from transferrin.^[^ ^56^, ^57^ ^]^ Fe^3^⁺ is then reduced to Fe^2^⁺ by the six‐transmembrane epithelial antigen of the prostate 3 (STEAP3) ferrireductase.^[^ ^58^ ^]^ Fe^2^⁺ is transported out of the endosome into the cytosol by DMT1.^[^ ^59^ ^]^

Within the cytosol, iron participates in various cellular processes or is stored to prevent toxicity.^[^ ^60^ ^]^ The LIP consists of low‐molecular‐weight, weakly chelated Fe^2^⁺ and represents the metabolically active and redox‐active fraction of intracellular iron.^[^ ^61^ ^]^ Excess iron is sequestered by ferritin, a heteropolymeric protein composed of heavy (FTH1) and light (FTL) chains, which can store up to 4500 iron atoms in a non‐toxic, bioavailable form.^[^ ^62^, ^63^ ^]^ Ferritin acts as a buffer, mitigating fluctuations in iron levels and protecting cells from iron‐induced oxidative damage.^[^ ^64^ ^]^

Under pathological conditions, iron homeostasis may be disrupted at various levels, including absorption, systemic transport, and cellular uptake and storage. Cancer cells exhibit dysregulation of iron homeostasis and an increased labile iron pool (LIP). This loosely bound iron can catalyze the Fenton reaction and possibly even other reactions that produce reactive oxygen species, resulting in cell death or cell transformation.^[^ ^65^ ^]^ In the context of ferroptosis, dysregulation of iron metabolism leads to increased intracellular iron levels, particularly in the LIP, promoting ROS generation through Fenton chemistry.^[^ ^66^ ^]^ The Fe^2^⁺ in the LIP reacts with hydrogen peroxide to produce hydroxyl radicals (•OH), potent oxidizing agents that initiate lipid peroxidation. Ferritinophagy, the selective autophagic degradation of ferritin mediated by the cargo receptor nuclear receptor coactivator 4 (NCOA4), increases the LIP by releasing stored iron from ferritin.^[^ ^67^, ^68^ ^]^ Upregulation of ferritinophagy enhances ferroptosis susceptibility by elevating intracellular iron availability.

Conversely, the overexpression of ferritin or the inhibition of ferritinophagy can reduce the LIP and protect cells from ferroptosis. Iron chelators such as deferoxamine (DFO) sequester free iron, inhibiting Fenton reactions and preventing lipid peroxidation, thereby rescuing cells from ferroptosis.^[^ ^69^, ^70^ ^]^ Iron export via ferroportin can also influence ferroptosis sensitivity. Reduced expression or activity of ferroportin leads to iron accumulation within cells, increasing the risk of ferroptosis. The regulation of ferroportin by hepcidin, inflammatory cytokines, and the cellular iron status can thus indirectly affect ferroptosis.^[^ ^30^, ^71^ ^]^ Is the role of iron in ferroptosis limited to induction, or are there other iron‐dependent genes involved downstream during lipid peroxidation? To date, there is no clear answer.^[^ ^72^ ^]^

Mitochondria are critical sites for iron utilization, particularly for the synthesis of heme and iron–sulfur (Fe‐S) clusters, which are essential cofactors for numerous proteins involved in electron transport, TCA cycle activity, and DNA metabolism.^[^ ^73^, ^74^ ^]^ Disruption of iron‐sulfur cluster assembly or heme synthesis can affect mitochondrial function and redox homeostasis, potentially influencing ferroptosis.^[^ ^74^, ^75^ ^]^ Mitochondrial iron uptake is mediated by transporters like mitoferrin‐1 (Mfrn1, SLC25A37) and mitoferrin‐2 (Mfrn2, SLC25A28).^[^ ^76^, ^77^ ^]^ While essential, mitochondrial iron overload or defects in Fe‐S cluster biogenesis can contribute to mitochondrial dysfunction, ROS production, and potentially sensitize cells to ferroptosis.^[^ ^78^ ^]^ Fe–S clusters‐containing enzymes, such as aconitase and complexes of the electron transport chain, are sensitive to oxidative damage, leading to mitochondrial dysfunction.^[^ ^73^, ^79^ ^]^ Targeting iron metabolism can sensitize cancer cells to ferroptosis, offering a therapeutic strategy.^[^ ^80^ ^]^

Systemic iron homeostasis is regulated primarily by hepcidin. At the cellular level, iron homeostasis is regulated post‐transcriptionally by iron regulatory proteins (IRP1 and IRP2), which bind to iron‐responsive elements (IREs) on target mRNAs. Under low iron conditions, IRPs bind to IREs in the untranslated regions (UTRs) of mRNAs to modulate the expression of proteins involved in iron metabolism.^[^ ^53^ ^]^ Binding of IRPs to IREs in the 5′ UTR represses translation (e.g., ferritin and ferroportin), while binding to the 3′ UTR stabilizes mRNAs (e.g., TfR1 and DMT1), thus enhancing iron uptake and reducing iron storage and export.^[^ ^53^, ^81^ ^]^ And transcription factors such as nuclear factor erythroid 2–related factor 2 (Nrf2) also modulate the expression of genes involved in iron metabolism and antioxidant defenses.^[^ ^82^, ^83^ ^]^

#### 2.1.3 Biological Significance of Ferroptosis: Roles in Health and Disease

Ferroptosis was initially explored for its potential in cancer therapy and is now recognized to play crucial roles in a wide array of physiological and pathological processes. Emerging evidence suggests roles for ferroptosis in normal development, immune surveillance, and elimination of damaged cells.^[^ ^84^, ^85^ ^]^

Pathologically, ferroptosis has been implicated as a key driver of tissue damage in ischemia‐reperfusion injury (IRI) across various organs, including the heart, kidney, brain, and liver.^[^ ^86^, ^87^, ^88^, ^89^ ^]^ In neurodegenerative diseases such as Alzheimer's disease, Parkinson's disease, and Huntington's disease, iron accumulation and lipid peroxidation are common features, and ferroptosis is increasingly recognized as a contributing mechanism to neuronal death.^[^ ^90^, ^91^, ^92^ ^]^

In the context of cancer, ferroptosis exhibits a dual role: it can act as an endogenous tumor suppressor pathway, and conversely, inducing ferroptosis in cancer cells has emerged as a promising therapeutic strategy, particularly for therapy‐resistant tumors. Intrinsically, mesenchymal and dedifferentiated cancer cells, which are usually resistant to apoptosis and traditional therapies, are exquisitely vulnerable to ferroptosis.^[^ ^93^, ^94^ ^]^ However, cancer cells can also develop mechanisms to evade ferroptosis. Drug‐tolerant persister cancer cells are vulnerable to GPX4 inhibition.^[^ ^93^, ^95^, ^96^ ^]^ Furthermore, ferroptosis contributes to the pathogenesis of acute kidney injury, non‐alcoholic steatohepatitis (NASH), lung diseases, infectious diseases, and various inflammatory conditions.^[^ ^30^, ^97^, ^98^ ^]^

The therapeutic implications are significant. Ferroptosis inducers (e.g., erastin, RSL3, sorafenib, sulfasalazine, and newer generation compounds) are being actively investigated as anti‐cancer agents.^[^ ^99^, ^100^, ^101^, ^102^ ^]^ Conversely, ferroptosis inhibitors (e.g., ferrostatin‐1, liproxstatin‐1, vitamin E) hold promise for mitigating tissue damage in conditions like IRI and neurodegeneration.^[^ ^103^, ^104^, ^105^ ^]^

### 2.2 Cuproptosis: A Copper‐Driven Pathway of Mitochondrial Proteotoxic Cell Death

#### 2.2.1 Discovery and Core Molecular Mechanisms of Cuproptosis

Prior to the formal naming of cuproptosis, studies with certain copper‐binding compounds had hinted at unique mechanisms of copper‐induced cell death. For instance, the anti‐cancer agent elesclomol, a small molecule initially investigated for its ability to induce oxidative stress, was found to exert its cytotoxic effects in a manner strictly dependent on the presence of copper. Research by Tsvetkov and colleagues in 2019 demonstrated that elesclomol complexes with copper ions, facilitating their delivery into cells and specifically targeting mitochondria, leading to the induction of mitochondrial oxidative stress and cell death, particularly in cells with high rates of mitochondrial respiration.^[^ ^106^ ^]^ While this study did not define a new RCD pathway per se, it underscored that copper, when delivered appropriately, could trigger a distinct form of mitochondrial‐centric cell death that was not simply a consequence of generalized copper poisoning. This work set the stage by highlighting a specific vulnerability related to copper and mitochondrial metabolism, suggesting that precise molecular interactions are at play.

The pivotal moment in the definition of cuproptosis came with a subsequent study in 2022.^[^ ^107^ ^]^ This research adopted a hypothesis‐free, unbiased genome‐wide CRISPR‐Cas9 loss‐of‐function screen to identify genes whose inactivation would confer resistance to cell death induced by copper ionophores. This systematic approach, screening for genetic modifiers of copper sensitivity, is a powerful tool for uncovering novel pathway components and was instrumental in delineating the unique mechanism of cuproptosis. The screens revealed a striking enrichment of genes involved in mitochondrial metabolism, particularly those associated with the tricarboxylic acid (TCA) cycle and, crucially, protein lipoylation.

Among the top genetic hits whose loss conferred resistance to copper‐induced death were *FDX1* (Ferredoxin 1) and genes involved in the lipoic acid synthesis and conjugation pathway. These genes included *LIAS* (lipoic acid synthetase), *LIPT1* (lipoic acid transferase 1, which attaches lipoic acid to the E2 components of α‐ketoacid dehydrogenases), *DLAT* (dihydrolipoamide S‐acetyltransferase, the E2 component of the pyruvate dehydrogenase complex (PDC)), and *DLST* (dihydrolipoamide S‐succinyltransferase, the E2 component of the α‐ketoglutarate dehydrogenase complex (OGDC)).

These genetic findings strongly implicated mitochondrial protein lipoylation as a central process in copper‐induced cell death. The researchers then demonstrated that excess intracellular copper, likely facilitated in its action or redox state by FDX1 (which can reduce Cu^2^⁺ to the more reactive Cu⁺), directly targets the lipoic acid moieties covalently attached to DLAT and DLST. Lipoic acid is a sulfur‐containing cofactor essential for the catalytic activity of these α‐ketoacid dehydrogenase complexes. The binding of copper to these lipoylated proteins was shown to induce their oligomerization and aggregation, leading to the formation of insoluble protein clumps within the mitochondria. This aggregation, in turn, caused profound proteotoxic stress within the organelle, a loss of function of the PDC and OGDC, and a collapse of the TCA cycle. A critical downstream consequence of this targeted protein aggregation was the destabilization and loss of other mitochondrial iron‐sulfur (Fe‐S) cluster‐containing proteins, further crippling mitochondrial respiration and metabolic function.

Crucially, this copper‐induced cell death pathway was found to be mechanistically distinct from other known RCDs. It was not inhibited by blockers of apoptosis, necroptosis, or ferroptosis. Furthermore, hallmarks of ferroptosis, such as widespread lipid peroxidation, were not primary features of this copper‐induced death. Based on these unique mechanistic features—copper dependence, targeting of lipoylated mitochondrial proteins, FDX1 involvement, protein aggregation, and insensitivity to inhibitors of other RCDs—the authors coined the term “cuproptosis” to define this novel cell death modality.

Since then, several outstanding studies have enriched the mechanism. Gale et al. demonstrated that ES/Cu induces FDX1‐independent astrocyte toxicity mediated by oxidative stress, as FDX1 knockdown did not block ES/Cu toxicity to astrocytes. Additionally, the inhibition of mitochondrial respiration failed to rescue the ES/Cu toxicity,^[^ ^108^ ^]^ and intracellular copper can be released from the ES/Cu complex and become bioavailable outside the mitochondria, suggesting a high likelihood of mitochondria‐independent cuproptosis.^[^ ^109^ ^]^ Similarly, DSF/Cu can mediate the aggregation and lock the conformational transition of cytoplasmic p97 complex, which plays a central role in cellular protein homeostasis, thereby inhibiting cellular ubiquitin‐proteasome degradation pathways and further leading to increased proteotoxic stress and cell death.^[^ ^110^, ^111^ ^]^ These findings highlight the existence of mitochondria‐independent cuproptosis, although the molecular mechanisms remain inadequately understood.

**Figure 2:**
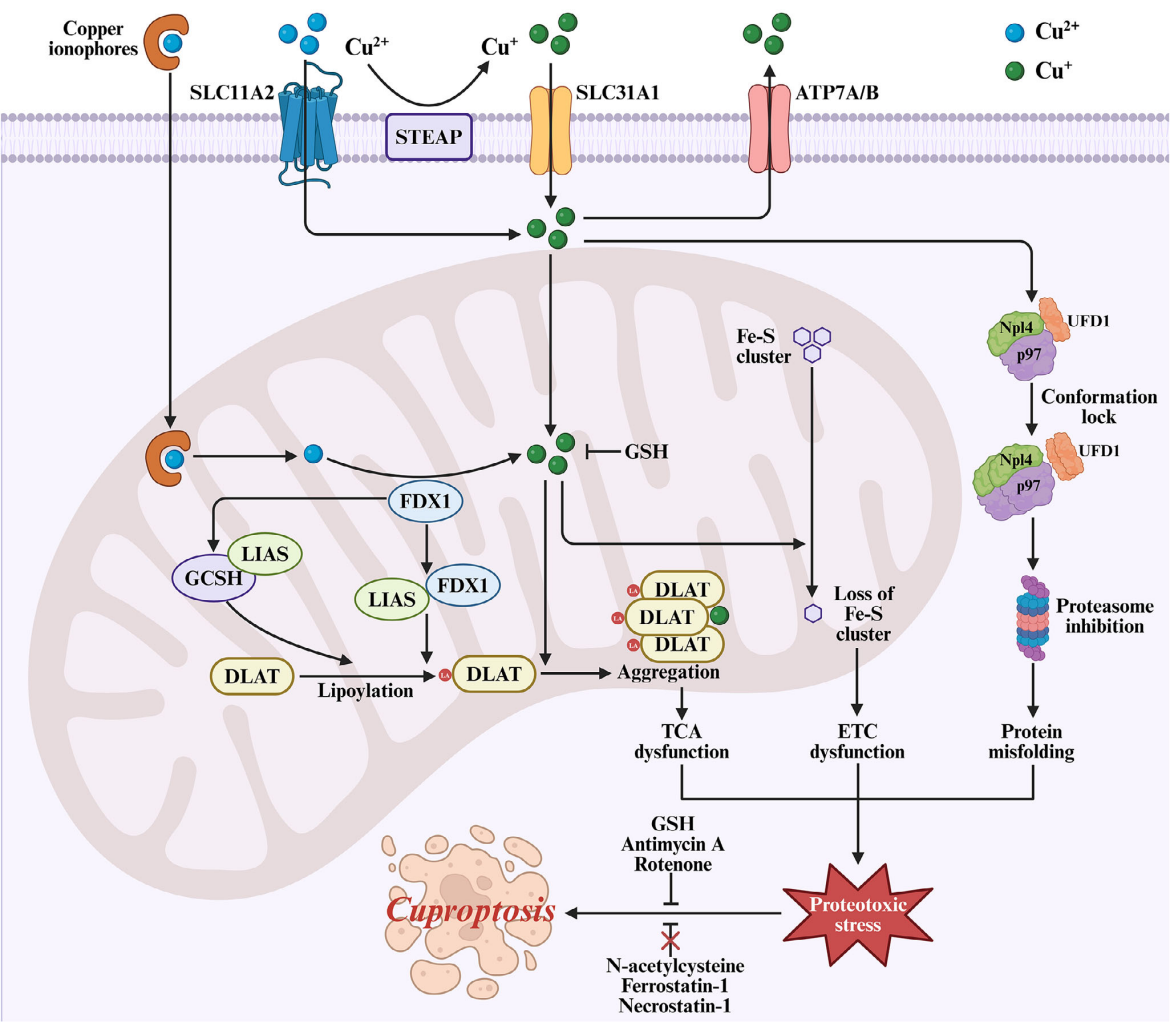
Molecular mechanisms of cuproptosis. The mechanism of cuproptosis involves both mitochondria‐dependent and mitochondria‐independent pathways. Extracellular copper enters the cell as Cu^2+^ through the SLC11A2 transporter. Within the cytoplasm, STEAP metalloreductase converts Cu^2+^ to Cu^+^, which is subsequently transported to mitochondria via SLC31A1 or exported via ATP7A/B transporters. In mitochondria, FDX1 has dual functions: reducing Cu^2+^ to the more toxic Cu^+^ and promoting protein lipoacylation. FDX1 directly binds to LIAS and enhances its interaction with GCSH, together promoting the lipoylation of DLAT. Cu^+^ binding induces the aggregation of DLAT and destabilizes Fe‐S cluster proteins, disrupting TCA cycle and ETC, respectively. Additionally, DSF/Cu mediates the aggregation and conformation lock of the Npl4‐p97 complex in the cytoplasm, inhibiting the ubiquitin proteasome degradation pathway, which contributes to protein misfolding. The combined effects of TCA dysfunction, ETC dysfunction, and protein misfolding produce proteotoxic stress, ultimately triggering cuproptosis. GSH, antimycin A, and rotenone can inhibit this process, while N‐acetylcysteine, ferrostatin‐1 and necrostatin‐1 do not confer such protection.

The entire cascade, from copper entry to mitochondrial collapse, is graphically summarized in **Figure** **2**.

#### 2.2.2 Copper Metabolism and Homeostasis

Like iron metabolism, copper metabolism is tightly regulated through a complex network of proteins responsible for its absorption, distribution, utilization, storage, and excretion to maintain cellular homeostasis and prevent toxicity.

Copper transporter 1 (CTR1), also known as SLC31A1, is a transmembrane protein that mediates copper uptake not only in enterocytes but also in other cell types throughout the body. The expression of CTR1 is regulated by copper levels.^[^ ^112^, ^113^ ^]^ Once inside the cell, copper ions are bound by specialized chaperone proteins to prevent free copper from participating in harmful redox reactions. These chaperones facilitate the safe transport of copper to specific intracellular destinations, ensuring proper utilization while minimizing toxicity. Key copper chaperones include Antioxidant Protein 1 (ATOX1),^[^ ^114^ ^]^ which delivers copper to the trans‐Golgi network for incorporation into copper‐dependent enzymes and for export via the copper‐transporting ATPases ATP7A and ATP7B;^[^ ^115^ ^]^ COMMD1 (formerly known as MURR1) is a membrane transport‐related protein that specifically binds Cu to one methionine and two histidine residues in a 1:1 ratio. COMMD1 interacts directly with ATP7A and ATP7B and is thought to be a regulator of Cu homeostasis.^[^ ^116^, ^117^, ^118^ ^]^ Copper Chaperone for Superoxide Dismutase (CCS) supplies copper to cytosolic superoxide dismutase 1 (SOD1), an enzyme critical for detoxifying superoxide radicals^[^ ^119^, ^120^ ^]^ Additionally, Cytochrome c Oxidase Copper Chaperones (COX17, COX11, SCO1, and SCO2) direct copper to the mitochondria for assembly into cytochrome c oxidase, which is essential for the electron transport chain.^[^ ^121^, ^122^ ^]^ These chaperones ensure that copper reaches its target enzymes efficiently, supporting essential cellular functions while preventing the accumulation of free copper ions that can induce oxidative stress.

To prevent copper toxicity, excess intracellular copper is sequestered by metallothioneins, which are low‐molecular‐weight, cysteine‐rich proteins capable of binding multiple metal ions through thiolate bonds.^[^ ^123^ ^]^ Metallothioneins act as intracellular copper buffers, mitigating potential toxicity by binding free copper ions and regulating their availability for metabolic processes. The liver plays a central role in systemic copper homeostasis. Hepatocytes incorporate copper into ceruloplasmin, the primary copper‐carrying protein in the blood that also exhibits ferroxidase activity, facilitating iron metabolism.^[^ ^124^ ^]^ Excess copper is excreted into bile via the copper‐transporting ATPase ATP7B, enabling elimination through the gastrointestinal tract.^[^ ^125^ ^]^

Cuproptosis is triggered when this homeostatic balance is disrupted, leading to an excess of intracellular copper that overwhelms the cell's buffering capacity, particularly within mitochondria. Conditions that can lead to such copper overload include genetic defects, exposure to copper ionophores, or potentially high environmental copper exposure.^[^ ^29^, ^126^ ^]^ The interplay among systemic copper levels, the cellular copper handling machinery, the mitochondrial metabolic state (determining the abundance of lipoylated TCA cycle enzymes), and the activity of FDX1 collectively determines a cell's susceptibility to cuproptosis. These aspects, primarily elucidated through in vitro experiments often involving genetic manipulation of these components in cultured cell lines, highlight multiple potential points for therapeutic intervention.

#### 2.2.3 Biological Significance and Challenges

This is currently the most active area for exploring the translational potential of cuproptosis. Many cancer cells exhibit a distinct copper metabolism phenotype compared to normal cells. They often show increased copper uptake and higher intracellular copper concentrations. These elevated copper levels are thought to be necessary to support rapid proliferation, angiogenesis (as many angiogenic factors are cuproenzymes), and altered mitochondrial metabolism.^[^ ^127^, ^128^ ^]^ This “copper addiction” of some tumors might paradoxically create a specific vulnerability that can be exploited by cuproptosis‐inducing strategies.

Several approaches are being investigated to leverage cuproptosis as an anti‐cancer strategy. Copper ionophores, such as elesclomol and disulfiram, have shown anti‐cancer activity in preclinical models. Their mechanisms are increasingly linked to cuproptosis induction.^[^ ^129^, ^130^, ^131^ ^]^ Furthermore, strategies aimed at modulating FDX1 activity or the abundance of lipoylated proteins specifically in cancer cells could offer more targeted ways to induce or sensitize tumors to cuproptosis. Nanomedicine approaches are also being developed to selectively deliver copper or copper‐based drugs to tumors, aiming to achieve therapeutic intracellular copper concentrations while minimizing systemic toxicity.^[^ ^132^, ^133^ ^]^ A key challenge in this field is to identify biomarkers—such as the expression levels of CTR1, FDX1, LIAS, DLAT/DLST, mitochondrial respiratory capacity, or baseline copper levels—that can predict which tumors will be most sensitive to cuproptosis‐based therapies.^[^ ^134^ ^]^

Wilson's Disease, an autosomal recessive disorder caused by mutations in the ATP7B gene, leads to toxic copper accumulation primarily in the liver and brain. This accumulation causes hepatic cirrhosis, liver failure, and severe neurological and psychiatric symptoms.^[^ ^135^, ^136^ ^]^ While the precise mechanisms of cell death in Wilson's disease are complex and likely involve oxidative stress and apoptosis, the core tenets of cuproptosis—copper targeting mitochondrial proteins, leading to mitochondrial dysfunction—could significantly contribute to the observed hepatocyte and neuronal damage. This is especially relevant given the high mitochondrial content and activity in these cells. Recent studies have begun to investigate cuproptosis markers and mechanisms in models of Wilson's disease, which may shed light on these possibilities.^[^ ^137^ ^]^

In neurobiology, the high copper content and metabolic demands of the brain make it a critical area of study for cuproptosis. Dysregulated copper homeostasis is implicated in neurodegenerative diseases like Alzheimer's and Parkinson's diseases.^[^ ^138^, ^139^ ^]^ Copper can bind to amyloid‐β peptide in AD, potentially modulating its aggregation and toxicity,^[^ ^140^ ^]^ and is also involved in α‐synuclein biology in PD.^[^ ^141^ ^]^ While direct evidence linking cuproptosis to these conditions in vivo is still limited, the high metabolic activity and mitochondrial dependence of neurons make them plausible targets if intracellular copper levels become dysregulated. This remains a speculative but highly active area of research, primarily utilizing in vitro neuronal models or animal models of copper dyshomeostasis.

The biological significance of cuproptosis is thus multifaceted, extending from fundamental cellular responses to metal stress to potential roles in a variety of human diseases. The field is young, and much research is needed, particularly in vivo studies using sophisticated genetic models and robust biomarkers.

### 2.3 Detection and Characterization of Ferroptosis and Cuproptosis

Both ferroptosis and cuproptosis require a systematic approach to detection. No single assay can definitively identify either form of cell death, necessitating integrated approaches combining cellular phenomena observation with molecular mechanism analysis.

The initial detection strategy for both pathways involves standard cell viability assays, including lactate dehydrogenase (LDH) release^[^ ^142^ ^]^ and MTT/CCK8 assays.^[^ ^143^ ^]^ However, these methods cannot distinguish metal‐dependent cell death from other forms of necrosis. The key differentiating factor is demonstrating rescue by specific inhibitors. Importantly, cell death in both pathways should not be prevented by inhibitors of apoptosis (Z‐VAD‐FMK), necroptosis (necrostatin‐1), pyroptosis (Ac‐YVAD‐cmk), or autophagy (bafilomycin A1).

Morphologically, ultrastructural analysis using transmission electron microscopy (TEM) reveals distinct morphological hallmarks for each pathway, primarily affecting the mitochondria. Cells undergoing ferroptosis typically exhibit mitochondrial shrinkage, an increase in mitochondrial membrane density, a reduction or complete disappearance of mitochondrial cristae, and occasional rupture of the outer mitochondrial membrane. Crucially, unlike apoptosis, ferroptosis does not involve significant chromatin condensation, nuclear fragmentation, or the formation of apoptotic bodies. Under light microscopy, some cells undergoing ferroptosis may appear to swell and rupture, exhibiting a “ballooning” morphology before lysis. Cuproptosis also presents with characteristic mitochondrial alterations, although these differ from ferroptosis. TEM studies of cells undergoing cuproptosis reveal mitochondrial swelling, disruption, or loss of cristae, and loss of inner mitochondrial membrane integrity. A key distinguishing feature is the aggregation of lipoylated mitochondrial enzymes, which can sometimes be observed as electron‐dense aggregates within the mitochondria. Similar to ferroptosis, significant nuclear changes typical of apoptosis, such as chromatin condensation or nuclear fragmentation, are generally absent in cuproptosis.

Biochemically, the hallmark of ferroptosis is the iron‐dependent accumulation of lipid peroxides. This can be detected by measuring lipid peroxidation products like malondialdehyde (MDA) and 4‐hydroxynonenal (4‐HNE) using assays such as the thiobarbituric acid reactive substances (TBARS) assay.^[^ ^144^ ^]^ Fluorescent probes, notably C11‐BODIPY 581/591, are widely used to detect lipid peroxidation in live cells, exhibiting a fluorescence shift from red to green upon oxidation.^[^ ^145^ ^]^ Given the crucial role of the GSH/GPX4 axis in suppressing ferroptosis, decreased levels of glutathione (GSH) and diminished GPX4 activity are key biochemical indicators. The labile iron pool (LIP), representing redox‐active intracellular iron, is often elevated in ferroptosis and can be measured using iron‐sensitive fluorescent probes^[^ ^146^ ^]^ or by quantifying total cellular iron using techniques like inductively coupled plasma mass spectrometry (ICP‐MS).^[^ ^147^ ^]^ For cuproptosis, the primary biochemical events center on copper accumulation and its consequences for mitochondrial proteins. Intracellular copper levels can be quantified using ICP‐MS or atomic absorption spectroscopy (AAS).^[^ ^148^, ^149^ ^]^ Live‐cell imaging of copper can be achieved with fluorescent copper‐binding probes such as CS1^[^ ^150^ ^]^ or mitochondria‐targeted sensors (NFL‐TPA, Triphenylamine–pyridine derivative).^[^ ^151^, ^152^ ^]^

Mitochondrial dysfunction is central to cuproptosis and can be assessed by measuring the mitochondrial membrane potential (Δψm) using dyes like JC‐1 (a decrease indicates dysfunction),^[^ ^153^ ^]^ and by determining the oxygen consumption rate (OCR) via Seahorse XF Analyzer (a decrease suggests impaired respiration).^[^ ^154^ ^]^ A distinguishing biochemical marker of cuproptosis is the aggregation of specific lipoylated mitochondrial proteins, and a critical downstream consequence is the destabilization and loss of various Fe‐S cluster‐containing proteins. These can be detected by Western blotting for specific Fe‐S cluster proteins or by measuring the decreased enzymatic activity of other Fe‐S cluster enzymes like cytosolic or mitochondrial aconitase.^[^ ^155^ ^]^

The expression levels of genes and proteins central to each pathway serve as molecular markers. For ferroptosis, alterations in the expression of *GPX4*, *SLC7A11* (a component of the system Xc^−^ cystine/glutamate antiporter), *ACSL4* (involved in incorporating PUFAs into lipids), and *TFRC* (transferrin receptor 1) are informative.^[^ ^44^, ^156^ ^]^ For instance, decreased expression or protein levels of GPX4 or SLC7A11, or increased expression of ACSL4 or TFRC, can indicate sensitization or induction of ferroptosis. In cuproptosis, the expression and activity of FDX1 are key molecular markers. Upregulation of *FDX1* enhances susceptibility to cuproptosis, while its knockdown confers resistance. Similarly, genes involved in the protein lipoylation pathway, including lipoic acid synthetase (*LIAS*), lipoyltransferase 1 (*LIPT1*), and the target genes themselves (*DLAT*, *DLST*), are important markers. Genetic manipulations, such as CRISPR‐Cas9‐mediated gene knockout or siRNA‐mediated knockdown of these key regulatory genes, are crucial for confirming their role in modulating sensitivity to either ferroptosis or cuproptosis.^[^ ^157^ ^]^

Specific pharmacological agents are instrumental in identifying and studying these pathways. Ferroptosis can be induced by compounds like erastin, which inhibits system Xc^−^, and RSL3, which directly inhibits GPX4.^[^ ^36^, ^158^ ^]^ Conversely, ferroptosis can be inhibited by lipophilic radical‐trapping antioxidants such as ferrostatin‐1 (Fer‐1) and liproxstatin‐1 (Lip‐1), or by iron chelators like deferoxamine (DFO).^[^ ^41^ ^]^ Cuproptosis can be induced by copper ionophores like elesclomol, which increase intracellular copper levels, or by direct exposure to high concentrations of copper salts. Disulfiram, particularly in complex with copper, can also promote cuproptosis‐like cell death. Inhibition of cuproptosis can be achieved using copper chelators such as tetrathiomolybdate (TM) or bathocuproinedisulfonic acid (BCS).^[^ ^159^ ^]^ Additionally, modulation of the lipoylation pathway or the use of mitochondrial protective agents can influence cuproptosis sensitivity.^[^ ^160^ ^]^ The specific rescue of cell death by these respective inhibitors is a critical criterion for defining the pathway.

While ROS are involved in both pathways, their nature and primary role differ. In ferroptosis, lipid ROS are the direct executioners. Specific probes like C11‐BODIPY 581/591 are used for their detection. General ROS probes like DCFH‐DA can also be used, but lack specificity for the critical lipid peroxides.^[^ ^161^ ^]^ For cuproptosis, ROS generation, particularly mitochondrial ROS (detectable with probes like MitoSOX Red), is considered more of a consequence of mitochondrial damage and metabolic collapse rather than the primary initiating event.^[^ ^162^ ^]^ Distinguishing the specific ROS species and their temporal relationship to cell death can help differentiate the pathways.

For in vivo studies, which are crucial for establishing physiological and pathological relevance, a combination of approaches is necessary. This involves using genetically engineered mouse models (e.g., conditional *Gpx4*‐knockout mice for studying ferroptosis in vivo, or *ATP7B*‐knockout mice as a model for copper overload potentially relevant to cuproptosis) and assessing tissues for pathway‐specific markers.^[^ ^163^, ^164^ ^]^ For example, immunohistochemical staining for 4‐HNE can detect lipid peroxidation in tissues susceptible to ferroptosis, while staining for DLAT aggregation could theoretically be developed for cuproptosis. Furthermore, testing the efficacy of specific inhibitors in ameliorating tissue damage in relevant disease models provides strong in vivo evidence for the involvement of a specific RCD pathway.

Recent technological advances have enhanced detection capabilities for both pathways. For ferroptosis, developments in mass spectrometry‐based lipidomics have enabled the precise characterization of oxidized phospholipid species.^[^ ^165^, ^166^ ^]^ And fluorogenic radical trapping antioxidants (RTAs) enable real‐time ferroptosis monitoring.^[^ ^167^, ^168^ ^]^ These organelle‐targeting probes reveal lipid hydroperoxide generation initially in the ER‐Golgi intermediate compartment, subsequently spreading throughout cellular membranes. Additionally, in conventional flow cytometry, Annexin V/7AAD double staining can be performed to assess further mechanisms of cell death.^[^ ^169^ ^]^ Multi‐parameter flow cytometry combines Annexin V/PI staining with TfR1 detection, distinguishing ferroptotic from apoptotic cells with 93% prediction accuracy.^[^ ^170^ ^]^ Moreover, image‐based machine learning has achieved remarkable accuracy in cell death discrimination. Multinomial logistic lasso regression analyzing morphological features distinguishes ferroptosis from apoptosis with 95% accuracy.^[^ ^171^ ^]^ For cuproptosis, novel electrochemical sensors using materials like Ti_3_C_2_T_x_/MWNTs‐Au offer enhanced sensitivity for copper detection,^[^ ^172^ ^]^ while advanced proteomics techniques facilitate identification of aggregated lipoylated proteins. Dynamic light scattering (DLS) and non‐denaturing gel electrophoresis provide complementary approaches for detecting protein aggregation characteristics of cuproptosis.^[^ ^173^, ^174^ ^]^

For a comprehensive comparison of detection methods and biomarkers across all three pathways, refer to **Table** **1**.

**Table 1:** Comprehensive comparison of ferroptosis, cuproptosis, and cobaltosis: mechanisms, detection, and distinct features.

Feature	Ferroptosis	Cuproptosis	Cobaltosis (Hypothetical)
Definition	Iron‐dependent regulated cell death characterized by lipid peroxidation and GPX4 inactivation^[^ ^284^ ^]^	Copper‐dependent regulated cell death through direct binding to lipoylated TCA cycle proteins^[^ ^107^ ^]^	Proposed cobalt‐dependent cell death with unique metabolic disruption patterns distinct from other metal‐dependent RCDs
Primary Mechanism	Iron‐catalyzed lipid peroxidation via Fenton reaction; GPX4 inactivation leads to PUFA oxidation^[^ ^30^ ^]^	Cu binds lipoylated proteins (DLAT/DLST); FDX1‐mediated protein aggregation; proteotoxic stress^[^ ^107^ ^]^	Unknown
Key Metabolic Regulation	Glutathione/GPX4 axis; System Xc^−^ cystine import; ACSL4‐mediated PUFA incorporation^[^ ^285^, ^286^ ^]^	Lipoic acid metabolism; TCA cycle disruption; mitochondrial respiration dependency^[^ ^27^ ^]^	Unknown
ROS Generation	Lipid ROS via iron‐catalyzed Fenton reaction; Mediated by oxidoreductases POR and CYB5R1^[^ ^48^ ^]^	Secondary consequence of mitochondrial dysfunction; not primary driver^[^ ^287^ ^]^	Direct Fenton‐like reactions;^[^ ^211^ ^]^ Indirect via metabolic conflict between hypoxic signaling and oxidative metabolism^[^ ^192^ ^]^
Mitochondrial Impact	Membrane lipid peroxidation; cristae↓; shrinkage; VDAC involvement^[^ ^36^, ^288^ ^]^	Direct targeting of the mitochondrial matrix, causing protein aggregation; swelling; cristae disruption^[^ ^107^ ^]^	Multi‐target dysfunction: ETC inhibition at complexes I/III;^[^ ^220^, ^221^ ^]^ Ca dysregulation;^[^ ^222^ ^]^ metabolic reprogramming via pseudohypoxia^[^ ^189^ ^]^
Metal Homeostasis Disruption	Iron accumulation in labile pool; ferritinophagy↑; transferrin receptor↑^[^ ^288^ ^]^	Copper overload exceeds buffering capacity; affect Fe‐S clusters secondarily^[^ ^289^ ^]^	Promiscuous metal‐interference: Zn,^[^ ^228^ ^]^ Ca,^[^ ^193^ ^]^ Mg,^[^ ^226^ ^]^ Fe^[^ ^189^ ^]^
Morphological Features	Mitochondrial shrinkage; membrane density↑; no nuclear changes^[^ ^288^ ^]^	Mitochondrial swelling; protein aggregates visible^[^ ^107^ ^]^	Membrane shrinkage; cell rounding^[^ ^222^ ^]^ nuclear/perinuclear cobalt accumulation^[^ ^218^, ^219^ ^]^
Biochemical Markers	MDA, 4‐HNE↑; GSH depletion; GPX4 activity↓; C11‐BODIPY oxidation	Lipoylated protein aggregation; OCR↓; Δψm↓; Fe‐S protein destabilization^[^ ^160^ ^]^	Unknown
Detection Methods	C11‐BODIPY (lipid ROS);^[^ ^145^ ^]^ TBARS assay (MDA)^[^ ^144^ ^]^ GPX4 activity/expression Iron levels: FerroOrange, ICP‐MS^[^ ^147^ ^]^	ICP‐MS/AAS (copper levels); Protein aggregation assay^[^ ^148^, ^149^ ^]^ OCR (Seahorse analyzer); JC‐1 (Δψm)^[^ ^290^ ^]^	Co probes: CP1, B, N‐CQDs^[^ ^291^, ^292^, ^293^, ^294^ ^]^
Molecular Markers	↑ACSL4, TFRC, HMOX1 ↓GPX4, SLC7A11 FSP1^[^ ^84^, ^86^, ^295^ ^]^	↑FDX1, LIAS, LIPT1 Aggregated DLAT/DLST^[^ ^107^ ^]^	Unknown
Inducers	Erastin, RSL3, sorafenib, IKE, FINO2^[^ ^100^ ^]^	Elesclomol, disulfiram/Cu, copper salts^[^ ^107^, ^159^ ^]^	Cobalt salts, cobalt nanoparticles, cobalt‐MOFs, organometallic complexes
Inhibitors	Ferrostatin‐1, liproxstatin‐1, vitamin E, DFO, α‐tocopherol^[^ ^105^, ^296^ ^]^	Copper chelators: Tetrathiomolybdate, BCS^[^ ^159^ ^]^	wAlb12 peptide^[^ ^271^ ^]^
Genetic Dependencies	GPX4, SLC7A11, ACSL4, FSP1, GCH1, DHODH^[^ ^98^, ^99^ ^]^	FDX1, LIAS, LIPT1, DLAT, DLST, ATP7A/B^[^ ^107^ ^]^	Unknown
Pathway Crosstalk	Trigger cuproptosis via Fe‐S cluster disruption; share GSH dependency^[^ ^297^ ^]^	Sensitize to ferroptosis through GSH depletion; affect iron metabolism^[^ ^297^ ^]^	Unknown
Clinical Relevance	Cancer therapy; neurodegeneration; IRI; NASH^[^ ^101^ ^]^	Wilson's disease; cancer; neurodegenerative diseases^[^ ^29^, ^137^ ^]^	Unknown
In Vivo Models	GPX4^−/−^ mice; erastin/RSL3 treatment models^[^ ^91^, ^92^ ^]^	ATP7B^−/−^ mice; elesclomol xenograft models^[^ ^107^ ^]^	CoCl_2_ exposure models;^[^ ^298^ ^]^ metal implant studies;^[^ ^299^ ^]^ nanoparticle toxicity models^[^ ^300^ ^]^
Unique Metabolic Features	Dependency on PUFA availability; glutaminolysis for GSH synthesis^[^ ^288^ ^]^	Dependency on mitochondrial respiration; lipoic acid metabolism^[^ ^107^ ^]^	Unknown

## 3 Exploring Cobalt‐Induced Cell Death (“Cobaltosis”)

### 3.1 Biological Significance of Cobalt

Cobalt is a trace element of significant biological importance, primarily recognized for its integral role in the structure and function of vitamin B_12_ (cobalamin). Vitamin B_12_ is essential for the proper functioning of the nervous system, the formation of red blood cells, and DNA synthesis.^[^ ^175^ ^]^ In vitamin B_12_, cobalt is centrally coordinated within a corrin ring structure, serving as a cofactor for two critical enzymes: methionine synthase and methylmalonyl‐CoA mutase.^[^ ^176^ ^]^ And almost all vitamin B_12_‐catalyzed reactions are rearrangements that occur through free radical reactions. To date, eight noncorrin Co‐dependent enzymes also have been isolated and characterized: methylmalonyl‐CoA carboxytransferase, prolidase, methionine aminopeptidase, nitrile hydratase, glucose isomerase, aldehyde decarbonylase, lysine‐2,3‐aminomutase, and bromoperoxidase.^[^ ^177^ ^]^

Beyond its incorporation into vitamin B_12_, free cobalt ions exhibit distinct chemical properties that influence cellular processes. Cobalt can exist in multiple oxidation states under physiological conditions, with Co^2+^ being the predominant form in biological systems.^[^ ^177^ ^]^ This redox activity, while less pronounced than that of iron or copper, enables cobalt to participate in Fenton‐like reactions, generating reactive oxygen species (ROS) that can damage cellular components.^[^ ^178^ ^]^ The ionic radius of Co^2+^ (0.74 Å) is similar to that of other divalent metal ions, such as Fe^2+^ (0.76 Å), Zn^2+^ (0.74 Å), and Mn^2+^ (0.80 Å), allowing cobalt to compete for binding sites on proteins and potentially disrupting normal metalloprotein function.^[^ ^179^ ^]^ For instance, cobalt competes with iron for binding sites on transport proteins like transferrin and receptors such as divalent metal transporter 1 (DMT1), affecting iron absorption and metabolism.^[^ ^180^ ^]^ Furthermore, cobalt influences the metabolism of other essential trace elements like manganese and zinc, potentially disrupting enzymatic activities dependent on these metals.^[^ ^181^ ^]^

The industrial and medical use of cobalt has increased human exposure beyond dietary sources. Cobalt alloys are widely used in orthopedic implants, particularly metal‐on‐metal hip replacements, which can release cobalt ions through wear and corrosion. This has led to cases of systemic cobalt toxicity, manifesting as cardiomyopathy, hypothyroidism, polycythemia, and neurological symptoms—collectively termed “arthroprosthetic cobaltism”.^[^ ^182^, ^183^, ^184^, ^185^, ^186^ ^]^ Additionally, occupational exposure in industries producing cobalt‐containing materials poses health risks, with inhalation of cobalt particles linked to interstitial lung disease and occupational asthma.^[^ ^187^, ^188^ ^]^

Cobalt also influences gene expression and cellular responses through its interaction with hypoxia‐inducible factors (HIFs). Under normoxic conditions, HIF‐1α is hydroxylated on specific proline residues by Fe^2^⁺‐ and 2‐oxoglutarate‐dependent prolyl hydroxylase domain enzymes (PHDs). This hydroxylation targets HIF‐1α for ubiquitination by the von Hippel‐Lindau (VHL) E3 ubiquitin ligase complex and subsequent proteasomal degradation. Cobalt ions (Co^2^⁺) can substitute for Fe^2^⁺ in the active site of PHDs or otherwise interfere with their activity, leading to the inhibition of HIF‐1α hydroxylation even in the presence of oxygen. This stabilizes HIF‐1α, allowing it to accumulate, translocate to the nucleus, dimerize with HIF‐1β (ARNT), and bind to hypoxia‐response elements (HREs) in the promoter regions of target genes.^[^ ^189^ ^]^ Therefore, cobalt plays a significant role in erythropoiesis—the production of red blood cells. It can stimulate erythropoietin (EPO) synthesis in the kidneys by activating HIF pathways, leading to increased red blood cell production.^[^ ^190^ ^]^ Historically, cobalt salts were used to treat certain types of anemia before the advent of recombinant EPO therapy.^[^ ^191^ ^]^ However, excessive cobalt intake can lead to polycythemia, which is characterized by an abnormally high concentration of red blood cells, increasing blood viscosity, and the risk of thrombosis.^[^ ^192^ ^]^

In environmental biology, cobalt plays a role in the nitrogen cycle through its presence in certain enzymes of nitrogen‐fixing bacteria and cyanobacteria. Cobalt‐containing corrinoid enzymes are involved in methyl transfer reactions essential for methanogenesis and other microbial metabolic pathways.^[^ ^177^ ^]^

### 3.2 Cobalt Homeostasis and Metabolism

Maintaining cobalt homeostasis is crucial to harnessing its benefits while preventing toxicity. The metabolism of cobalt involves a finely tuned balance of absorption, intracellular distribution, utilization, storage, and excretion.

In eukaryotic cells, including humans, the uptake of cobalt is less specific and often occurs via divalent metal transporters like DMT1, which also transports iron and other divalent metals.^[^ ^180^ ^]^ In human red blood cells, cobalt uptake occurs through passive transport pathways that are similar to those for calcium.^[^ ^193^ ^]^ In V79 cells, cobalt uptake is mediated by energy‐consuming processes such as ion pumps and endocytosis.^[^ ^194^ ^]^

Once inside human cells, cobalt accumulates in specific cellular compartments. In human keratinocytes, cobalt accumulates in the nucleus and perinuclear regions, potentially interacting with genomic DNA and nuclear proteins. It may also be stored in the endoplasmic reticulum or Golgi apparatus.^[^ ^195^ ^]^ Cobalt disrupts iron–sulfur clusters, leading to oxidative stress and necessitating the upregulation of genes involved in Fe‐S cluster biosynthesis and cobalt efflux.^[^ ^196^ ^]^ The ferroportin (FPN) metal efflux proteins also play a role in cobalt homeostasis in mammalian systems and thus influence both the iron deficiency response and sensitivity to cobalt.^[^ ^197^ ^]^

Prokaryotic organisms have evolved sophisticated mechanisms for cobalt transport and homeostasis. Cobalt uptake is facilitated by high‐affinity ATP‐binding cassette (ABC) transporters such as the CbiMNQO complex, which actively imports cobalt ions into the cell.^[^ ^198^ ^]^ TonB‐dependent systems are involved in the active transport of cobalt across the outer membrane of gram‐negative bacteria.^[^ ^199^ ^]^

Specific transporters have been identified in various bacterial species. In *Sinorhizobium meliloti*, the AitP transporter exports cobalt and iron.^[^ ^200^ ^]^ NhlF, which mediates cobalt uptake, has been identified in *Rhodococcus rhodochrous*.^[^ ^201^ ^]^ The RcnA transporter is involved in cobalt efflux in *Escherichia coli* and *Salmonella enterica*.^[^ ^202^ ^]^ Additionally, resistance‐nodulation‐cell division (RND) family transporters and P‐type ATPases catalyze the efflux of Co^2^⁺.^[^ ^199^ ^]^

In microorganisms, cobalt is allocated to metalloenzymes like cobalamin‐dependent enzymes through a cooperative network of accessory proteins.^[^ ^203^ ^]^ Specialized metalloproteins and chaperones, such as cobalt‐binding proteins, help stabilize cobalt ions and direct them to specific enzymatic functions. COG0523‐family proteins CobW2 and CobW3 control cobalt homeostasis. When zinc is scarce, the cell floods with cobalt ions to protect itself from cadmium toxicity and metal starvation.^[^ ^204^ ^]^

Regulatory systems maintain cobalt balance in bacteria. In *Vibrio parahaemolyticus*, the DmeRF system regulates cobalt homeostasis by promoting cobalt efflux and preventing toxic accumulation.^[^ ^205^ ^]^ The periplasmic manganese‐binding protein MntC is probably the main pathway for cobalt uptake and the locus for manganese and zinc competitive inhibition.^[^ ^206^ ^]^

Some eukaryotic microorganisms have distinct cobalt transport mechanisms. The *COT1* gene in *Saccharomyces cerevisiae* is involved in cobalt accumulation and confers increased tolerance to cobalt toxicity.^[^ ^207^ ^]^ In *Chlamydomonas reinhardtii*, the NRAMP1 transporter is crucial for cobalt and zinc transport.^[^ ^208^ ^]^

### 3.3 Cobalt's Known Cellular Impacts

Before postulating a novel RCD, it is essential to understand the well‐documented ways in which cobalt overload perturbs cellular homeostasis and induces damage. Cobalt ions (primarily Co^2^⁺) are known to exert pleiotropic effects, many of which can lead to cell death through mechanisms that may or may not involve a specific, regulated “cobaltotic” program. From the morphological experiment, scanning electron microscopy (SEM) showed that the morphology of the cells treated with cobalt ions changed significantly. Membrane shrinkage, cell rounding, loose cell junctions, and significant changes in cell morphology were observed.^[^ ^209^, ^210^ ^]^

The generation of oxidative stress represents one of the most well‐documented effects of cobalt exposure, although the mechanisms differ from those of the classical Fenton chemistry associated with iron. While Co^2+^ can participate in Fenton‐like reactions to generate hydroxyl radicals, the rate constants are significantly lower than those for iron.^[^ ^211^ ^]^ Instead, cobalt induces oxidative stress through multiple indirect mechanisms that collectively overwhelm cellular antioxidant defenses.^[^ ^192^ ^]^ Excess cobalt can increase LDH release and MDA concentration; decrease cell viability, SOD activity, GSH production, and CAT activity; inhibit antioxidant enzymes like catalase and superoxide dismutase; and promote lipid peroxidation.^[^ ^212^ ^]^ Cobalt exposure leads to rapid depletion of GSH. This occurs through direct binding of cobalt to the sulfhydryl groups of GSH, forming cobalt‐glutathione complexes that are exported from cells.^[^ ^213^ ^]^ Additionally, cobalt exposure decreases the activity of catalase and superoxide dismutase (SOD), although through different mechanisms.^[^ ^214^, ^215^ ^]^ These changes in antioxidant enzyme function contribute to the accumulation of hydrogen peroxide and superoxide radicals. This oxidative damage affects cellular membranes, proteins, and DNA, contributing to cell dysfunction and death. Moreover, cobalt can induce apoptosis and necrosis.^[^ ^216^, ^217^ ^]^

Mitochondria emerge as primary targets of cobalt toxicity, with dysfunction occurring through multiple interconnected mechanisms. Cobalt accumulation in mitochondria disrupts the electron transport chain at multiple sites.^[^ ^218^, ^219^ ^]^ Complex I shows particular sensitivity to cobalt, with inhibition occurring at concentrations achievable during systemic cobalt exposure. This inhibition appears to result from cobalt binding to iron‐sulfur clusters within the complex, disrupting electron transfer.^[^ ^220^, ^221^ ^]^ Complexes III and IV also show reduced activity, although to a lesser extent. The disruption of electron transport leads to increased electron leakage and superoxide generation at complexes I and III.^[^ ^222^ ^]^ This mitochondrial ROS production creates a feed‐forward cycle, as ROS can further damage electron transport chain components and mitochondrial DNA. The impairment of mitochondrial function can trigger the opening of the mitochondrial permeability transition pore (mPTP), leading to loss of membrane potential and release of pro‐apoptotic factors such as cytochrome c, which activate caspase‐dependent apoptotic pathways.^[^ ^222^ ^]^ This positions mitochondrial dysfunction as a potential point of convergence between cobaltosis and established cell death pathways.

The ability of cobalt to interfere with other metal‐dependent processes extends beyond simple competition for transporters. Cobalt can substitute for native metal cofactors in numerous metalloproteins, often resulting in proteins with altered or abolished functions.^[^ ^223^ ^]^ This metal substitution represents a potential mechanism for triggering regulated cell death if specific metalloenzymes critical for cell survival are affected. Cobalt can displace metals like calcium, magnesium, zinc, and iron from their biological binding sites. For instance, cobalt may act as a Ca^2 +^ channel antagonist(membrane transport pathway for Co^2 +^ uptake appears to be shared with Ca^2 +[^ ^193^ ^]^), inhibiting Ca^2 +^ entry and Ca^2 +^‐signaling and competition with Ca^2 +^ for intracellular Ca^2 +^‐binding proteins.^[^ ^224^ ^]^ And cobalt is not extruded by the Ca‐pump and is effectively bound to the globin moiety of hemoglobin, with the result that the concentration of free, ionized Co^2^⁺ in the cytosol is only about 1% of the total cobalt concentration.^[^ ^225^ ^]^ Similarly, a competitive mechanism with magnesium uptake is because cobalt can substitute for Mg in Mg^2+^ transporters.^[^ ^226^ ^]^ Interestingly, one study found that concentrations of sodium, magnesium, and calcium increased after cobalt treatment, while potassium concentration decreased.^[^ ^209^ ^]^

Zinc finger proteins represent another major target of cobalt substitution. The similar ionic radii and coordination preferences of Co^2+^ and Zn^2+^ allow cobalt to replace zinc in many zinc finger domains.^[^ ^227^, ^228^ ^]^ However, cobalt‐substituted zinc fingers often show altered DNA binding specificity or stability.^[^ ^229^ ^]^ Given the prevalence of zinc fingers in transcription factors, DNA repair enzymes, and signaling proteins, cobalt‐induced zinc finger dysfunction could have widespread cellular consequences. Iron‐sulfur cluster proteins are particularly vulnerable to cobalt interference. These ancient cofactors participate in diverse cellular processes, including mitochondrial respiration, DNA repair, and metabolic regulation. Cobalt can disrupt iron‐sulfur cluster assembly and stability.^[^ ^220^ ^]^ The resulting dysfunction of iron‐sulfur proteins could contribute to metabolic collapse similar to that observed in cuproptosis.

Cobalt exposure induces DNA damage through both direct and indirect mechanisms. Direct interactions between cobalt and DNA occur primarily at guanine bases, where cobalt can coordinate to N7 positions. This binding can destabilize the DNA double helix and interfere with DNA‐protein interactions.^[^ ^230^ ^]^ However, the indirect mechanisms of cobalt genotoxicity, mediated through oxidative stress and interference with DNA repair systems, appear more significant for cellular outcomes. The DNA damage response to cobalt involves activation of multiple checkpoint and repair pathways. Phosphorylation of H2AX (γH2AX) increases within hours of cobalt exposure. This is accompanied by the activation of ATM and ATR kinases, leading to p53 stabilization and cell cycle arrest.^[^ ^231^, ^232^, ^233^ ^]^ Cobalt also impairs DNA repair capacity, potentially converting repairable lesions into lethal damage.^[^ ^211^, ^234^ ^]^ Base excision repair (BER) and nucleotide excision repair (NER) pathways show reduced activity in cobalt‐exposed cells.^[^ ^235^, ^236^ ^]^ This may result from cobalt substitution in zinc finger domains of repair enzymes or from indirect effects on repair protein expression and post‐translational modifications.

In the immune system, cobalt modulates immune responses by affecting the inflammatory response of macrophages. At high concentrations, it promotes macrophage polarization towards an M1‐like phenotype and increases the expression and release of pro‐inflammatory cytokines such as the transcription factor TNF‐α, IL‐6, and NF‐κB.^[^ ^237^ ^]^ This may be related to mitochondrial dysfunction(decreased oxidative phosphorylation capacity), reduced ATP production, and activation of AMP‐activated protein kinase (AMPK).^[^ ^218^ ^]^

Perhaps the most challenging aspect of defining cobaltosis as a unique RCD pathway is that cobalt exposure, depending on the concentration, duration, and cell type, activates multiple established cell death mechanisms. Studies have documented cobalt‐induced apoptosis, characterized by caspase activation, phosphatidylserine externalization, and DNA fragmentation.^[^ ^216^, ^238^, ^239^ ^]^

Cobalt also induces autophagy, initially as a survival response to clear damaged proteins and organelles.^[^ ^240^ ^]^ However, excessive or prolonged autophagy can transition to autophagic cell death. The induction involves classic autophagy regulators, including AMPK activation (due to metabolic stress), mTOR inhibition, and upregulation of autophagy genes like BECN1 and ATG family members.^[^ ^241^, ^242^, ^243^ ^]^ Importantly, cobalt can induce mitophagy through BNIP3, linking HIF activation to selective mitochondrial clearance.^[^ ^244^ ^]^

In previous years, evidence has suggested that cobalt may also trigger ferroptosis‐like cell death under certain conditions. Cobalt's interaction with sulfur‐containing amino acids and thiol groups may impair antioxidant systems and contribute to ferroptosis‐like cell death.^[^ ^245^ ^]^ However, classical ferroptosis inhibitors show variable efficacy against cobalt toxicity, suggesting either incomplete overlap or cell‐type‐specific differences in death pathway activation.^[^ ^246^ ^]^

Given this activation of multiple RCD pathways, the critical question becomes whether cobaltosis represents: (1) a unique pathway with its own molecular mechanism that remains to be discovered, (2) a specific combination or sequence of known pathways triggered by cobalt's unique cellular effects, or (3) simply non‐specific toxicity activating whatever death pathways are primed in a given cell type.

**Figure 3:**
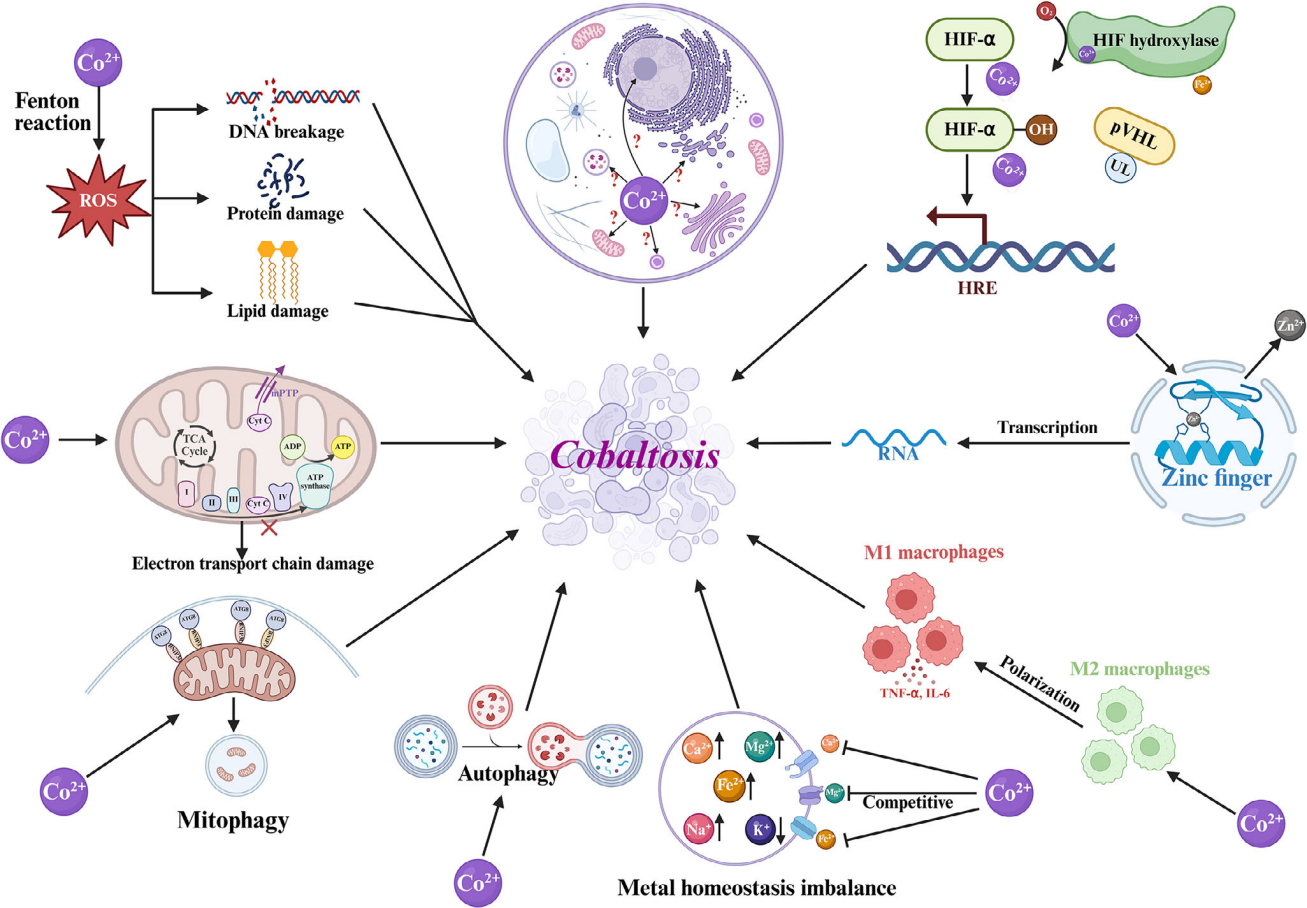
Potential Cellular Impact of Cobaltosis.

The multifaceted cellular effects of cobalt exposure are summarized in **Figure** **3**, which illustrates the potential mechanisms through which cobalt could induce a unique form of regulated cell death.

### 3.4 Defining the Quest: Uniqueness and Specificity of “Cobaltosis”

In the past, there was only a dichotomous approach to cell death, cobalt is already known to induce apoptosis and can cause necrosis at high concentrations. The validation of “cobaltosis” as a bona fide, distinct RCD pathway hinges critically on the identification and characterization of molecular events and regulatory networks that are specifically and predominantly initiated by cobalt ions and are mechanistically distinguishable from both non‐specific cobalt toxicity and other established forms of RCD, including ferroptosis, cuproptosis, apoptosis, and necroptosis(given the properties of cobalt, the focus should be to distinguish it from these four forms). This formidable task requires moving beyond simply observing that cells die in response to cobalt exposure.

Versus Apoptosis: Researchers must show that the putative cobaltosis proceeds independently of canonical apoptotic mechanism. This would involve demonstrating a lack of reliance on caspase activation, Bcl‐2 family protein regulation, and the absence of characteristic apoptotic morphology (e.g., formation of apoptotic bodies). If caspases are activated, it must be shown that they are either non‐essential for death or a late, secondary event.

Versus Necrosis/Necroptosis: If the morphology is necrotic, it must be distinguished from unregulated necrosis by demonstrating regulation and from regulated necroptosis by showing independence from the RIPK1‐RIPK3‐MLKL axis.

Versus Ferroptosis: Since cobalt can induce oxidative stress and interact with iron metabolism, it's conceivable and could indirectly promote ferroptosis. Cobaltosis must be shown to be independent of iron‐dependent lipid peroxidation and GPX4 activity. For example, rescue by iron chelators or ferroptosis inhibitors like ferrostatin‐1 would argue against a distinct cobaltosis.

Versus Cuproptosis: Cuproptosis involves copper binding to lipoylated mitochondrial proteins. Cobaltosis would need to demonstrate a different primary target and mechanism, even if mitochondria are involved. Rescue by copper chelators would also be indicative.

And several stringent criteria would need to be met to elevate “cobaltosis” beyond a hypothetical construct: 1) A Specific Initiating Molecular Event or Sensor: Ideally, “cobaltosis” would be triggered by a unique and direct interaction between cobalt (or some other specific form) and a specific cellular sensor molecule—be it a protein, a lipid, a nucleic acid, or a specific metabolite. This interaction should initiate a signaling cascade. Furthermore, the death pathway should be less efficiently triggered, or not triggered at all, by similar concentrations of other divalent metal ions known to share uptake pathways with cobalt. 2) Cobalt‐Specific Regulatory Proteins and Signaling Pathways: The pathway should involve key regulatory proteins (e.g., specific enzymes, adaptor proteins, and transcription factors) whose activity, expression, or localization are specifically modulated by cobalt (or by the immediate downstream consequences of the initial cobalt‐sensor interaction) to promote cell death. Genetic ablation or pharmacological inhibition of these specific components should selectively block “cobaltosis” without significantly impacting other cell death modalities initiated by different stimuli. 3) Distinct Biochemical and Morphological Signatures: While some overlap in downstream effector events is conceivable (e.g., mitochondrial involvement is common in many RCDs), “cobaltosis” should ideally exhibit a unique and reproducible profile of biochemical changes (e.g., accumulation or depletion of specific metabolites, unique post‐translational modifications of key proteins, specific patterns of ROS generation or lipid alteration distinct from ferroptosis) and/or characteristic ultrastructural morphological alterations (e.g., a specific pattern of organellar damage, unique cytoplasmic inclusions, or distinct membrane dynamics visualized by high‐resolution microscopy) that could serve as diagnostic hallmarks. 4) Evidence of Regulation: The process must be “regulated,” implying that it is genetically encoded and can be modulated by specific cellular interventions. This contrasts with accidental or uncontrolled cell lysis resulting from overwhelming chemical insult. Evidence of regulation could come from identifying specific sensitizers or inhibitors, or demonstrating a dependence on active cellular processes like transcription or translation for its execution.

Additionally, cobaltosis should demonstrate evolutionary conservation across species, suggesting fundamental biological importance rather than being merely an artifact of modern environmental exposure. The pathway must also show physiological or pathological relevance, occurring naturally under specific conditions rather than only under artificial experimental manipulation.

### 3.5 Speculating on Potential Molecular Features of a Distinct “Cobaltosis”

Macroscopically, cobalt might preferentially accumulate at, or disrupt communication between, specific organelles (e.g., ER‐mitochondria contact sites and lysosome‐autophagosome interactions) in a manner that triggers a unique stress signal distinct from general organellar damage. Microscopically, cobalt, due to its specific ionic radius and coordination chemistry, might preferentially bind to and stabilize or destabilize unique conformational states of certain proteins, or interact with specific allosteric sites not readily targeted by other metals. This could lead to a gain‐of‐toxic‐function or loss‐of‐essential‐function that is highly specific and initiates a programmed death response.

One possibility is that cobaltosis involves specific targeting of zinc finger proteins, given cobalt's ability to displace zinc from these structural motifs. This could lead to widespread transcriptional dysregulation through a mechanism distinct from other metal‐induced deaths. The accumulation of misfolded zinc finger proteins might trigger a unique proteotoxic stress response, potentially involving specialized protein quality control pathways not engaged in by cuproptosis.

Another speculative mechanism involves cobalt's unique ability to stabilize hypoxia‐inducible factors (HIFs) under normoxic conditions. This pseudohypoxic state could trigger a distinct metabolic catastrophe where cells simultaneously activate hypoxic responses while maintaining oxidative metabolism, creating an unsustainable metabolic conflict. The resulting energetic crisis might activate a unique cell death mechanism adapted to resolve such metabolic paradoxes. This mechanism would be particularly relevant in highly metabolic tissues like the heart and brain, potentially explaining the organ‐specific toxicities observed with cobalt exposure.

Cobalt's interaction with vitamin B_12_ metabolism presents another unique angle. Given that cobalt can compete with the cobalt center in cobalamin, high cobalt exposure might specifically disrupt one‐carbon metabolism in ways that don't occur with other metal toxicities. This could lead to unique epigenetic changes or nucleotide imbalances that trigger a specialized cell death response. The accumulation of metabolic intermediates normally processed by B_12_‐dependent enzymes might serve as death signals in a putative cobaltosis pathway.

### 3.6 Methods to Induce Potential Cobaltosis

Given that “cobaltosis” is not an established RCD pathway but a hypothesis under examination, methods for its induction must be viewed primarily as experimental tools designed to probe its existence and delineate its potential mechanisms. A key challenge is to induce a specific, regulated death program rather than simply causing generalized cellular damage that leads to apoptosis or necrosis. Therefore, the goal is not only to kill cells with cobalt, but also to do so in a manner that might reveal a unique underlying molecular program.

The most straightforward approach to investigate cobalt‐dependent cell death is to induce intracellular cobalt overload. However, the method of delivery can profoundly influence the outcome. The simplest method is the direct addition of cobalt salts, such as cobalt (II) chloride (CoCl_2_), to cell culture media. When employing these compounds in vitro, meticulous dose‐response curves and time‐course studies are essential to identify concentration ranges and exposure durations that elicit cell death without causing immediate, overwhelming necrotic lysis. The choice of concentration will be critical; researchers must aim for levels that are sublethal in the short term for non‐RCD pathways but sufficient to trigger the hypothesized cobaltosis cascade over a defined period. While it effectively increases intracellular cobalt levels, this method has significant limitations for the specific study of a regulated “cobaltosis.” The free Co^2^⁺ ions can engage in numerous non‐specific interactions, chelate with components of the culture medium, and their uptake is often mediated by promiscuous divalent cation transporters (like DMT1), making it difficult to control the precise intracellular dose and subcellular localization.

Beyond simple salts, organometallic cobalt complexes or specific cobalt coordination compounds (analogous to elesclomol for copper) could also be explored, as these may possess different uptake mechanisms, intracellular trafficking patterns, and reactivity profiles, potentially leading to more specific induction of cobaltosis or revealing unique aspects of cobalt's interaction with cellular machinery. For example, cobalt‐containing porphyrin complexes are used to mimic natural cobalt‐binding in vitamin B_12_, which enhances cellular uptake via receptor‐mediated endocytosis. Similarly, synthetic chelators such as the nucleophilic *meso*‐pyridyl‐containing porphyrin, oxaporphine cobalt (II) can be used to form stable complexes with cobalt, allowing for intracellular cobalt accumulation.^[^ ^247^, ^248^ ^]^ By conjugating these carriers with specific cell‐targeting ligands or antibodies, cobalt can be selectively delivered to particular cell populations, providing more precise models for studying cobalt‐induced toxicity in diseases such as cancer.

Nanotechnology also offers promising methods to induce cobaltosis via cobalt‐based nanomaterials. Cobalt nanoparticles (CoNPs) and cobalt oxide nanoparticles (Co_3_O_4_NPs) have garnered interest due to their unique physicochemical properties and ability to interact with biological systems at the nanoscale. Owing to their high surface area‐to‐volume ratios, these nanoparticles efficiently release cobalt ions intracellularly, either through dissolution or reactive surface interactions.^[^ ^249^, ^250^ ^]^ The small size of these nanoparticles allows them to enter cells via endocytosis, particularly through clathrin‐mediated and caveolae‐mediated internalization, which is generally more pronounced in cancer cells.^[^ ^251^ ^]^ Once inside the cell, cobalt ions are gradually released, causing an accumulation that leads to cobaltosis.^[^ ^252^ ^]^ Moreover, cobalt nanoparticles may exert additional toxicity beyond cobalt ion release, such as by generating ROS, which can further exacerbate oxidative stress and cellular damage.^[^ ^253^, ^254^ ^]^ This dual mechanism—ion release coupled with nanoparticle‐induced oxidative stress—provides a more comprehensive model of cobalt toxicity. Surface modifications, including a PMIDA coating for stability and folic acid conjugation, enable cancer cell targeting, with preferential cytoplasmic distribution, avoiding nuclear accumulation.^[^ ^255^, ^256^ ^]^

To induce cobaltosis effectively, a potential design is to develop cobalt‐doped metal‐organic frameworks (MOFs) or coordination polymer nanoplatforms that encapsulate cobalt within a porous structure, and selectively deliver cobalt to target cells. For instance, zeolitic imidazolate framework‐67 (ZIF‐67) can be modified with polyethylene glycol (PEG) chains containing acid‐labile bonds, creating a system that releases cobalt ions specifically in acidic compartments.^[^ ^257^ ^]^ To enhance selectivity and efficacy, these nanomaterials can be functionalized with targeting ligands that recognize overexpressed receptors on cancer cells.^[^ ^258^, ^259^, ^260^, ^261^ ^]^ To amplify the oxidative stress and overcome cellular antioxidant defenses, the nanomaterials can be co‐loaded with agents that deplete GSH. To achieve organelle‐specific targeting, nanomaterials can incorporate targeting ligands or peptides that are recognized by organelle‐specific receptors or transport mechanisms. For example, mannose‐6‐phosphate (M‐6‐P) facilitates lysosomal targeting, mitochondrial‐targeting moieties like triphenylphosphonium (TPP), while ER retention signals (the KDEL motif) and Golgi‐localization sequences can direct nanomaterials to the ER and Golgi, respectively.^[^ ^262^, ^263^, ^264^, ^265^ ^]^ Despite their promise, several challenges remain in using ion carriers and nanoparticles to study cobaltosis. The physicochemical properties of nanoparticles—such as size, surface charge, and solubility—can influence their biological interactions and toxicity profiles.^[^ ^266^, ^267^ ^]^

If a distinct “cobaltosis” pathway exists, it is by definition regulated. This implies the existence of cellular defense mechanisms that actively suppress this pathway. A more sophisticated and specific strategy to induce cobaltosis would therefore be to inhibit these putative defense pathways, thereby sensitizing cells to die via “cobaltosis” even at sub‐toxic cobalt concentrations. Overexpression of putative cobalt uptake systems or knockout of potential cobalt efflux mechanisms could sensitize cells to physiological cobalt concentrations.

### 3.7 Methods to Inhibit Potential Cobaltosis

Key strategies used to inhibit cobaltosis include reducing cobalt uptake, enhancing its elimination, mitigating its cellular effects, and restoring disrupted physiological functions. One primary approach is chelation therapy, which involves using chelating agents to bind cobalt ions, facilitating their excretion. Agents such as Na_2_Ca‐ethylenediaminetetraacetate (EDTA), Na_3_Ca‐diethylentriaminepentaacetate (DTPA), and 8‐hydroxyquinoline‐cyclodextrin conjugate have been used successfully to treat heavy metal poisoning.^[^ ^268^, ^269^, ^270^ ^]^ In addition to these non‐specific divalent metal chelators, a chelating peptide (wAlb12) targeting cobalt has recently been developed.^[^ ^271^ ^]^

Given cobalt's known pro‐oxidant potential, it is plausible that oxidative stress plays a significant role in cobaltosis, perhaps through mechanisms distinct from those in ferroptosis (which is iron‐ and lipid peroxidation‐centric). If specific types of ROS or particular oxidative targets are identified as hallmarks of cobaltosis, then antioxidants might serve as inhibitors. But if ROS are not the underlying mechanism, then the antioxidant approach has non‐specific limitations. General antioxidants such as N‐acetylcysteine (NAC) and vitamins C and E can replenish antioxidant defenses and neutralize ROS, protecting cellular components from oxidative damage.^[^ ^272^, ^273^, ^274^ ^]^ Polyphenolic compounds prevent cobalt‐mediated DNA damage by competing with ascorbate to bind Co^2+^ and reducing ROS generation.^[^ ^211^, ^275^ ^]^ Alpha‐lipoic acid uniquely alleviates cobalt nanoparticle‐induced ferroptosis‐like features by maintaining GPX4 activity and preventing lipid peroxidation.^[^ ^245^ ^]^ Mitochondria‐targeted antioxidants such as MitoQ show neuroprotective effects through selective accumulation.^[^ ^276^ ^]^

Modulating metal transporters offers a targeted approach, and inhibiting these transporters can reduce cobalt uptake. Genetic or pharmacological inhibition of these transporters may decrease intracellular cobalt accumulation. Additionally, increasing the activity of metal efflux transporters can enhance cobalt export from cells.^[^ ^197^ ^]^ Iron and zinc, which share common transport pathways with cobalt, may also influence their absorption; for instance, iron supplementation can reduce cobalt uptake through competition with the DMT1 transporter.

**Figure 4:**
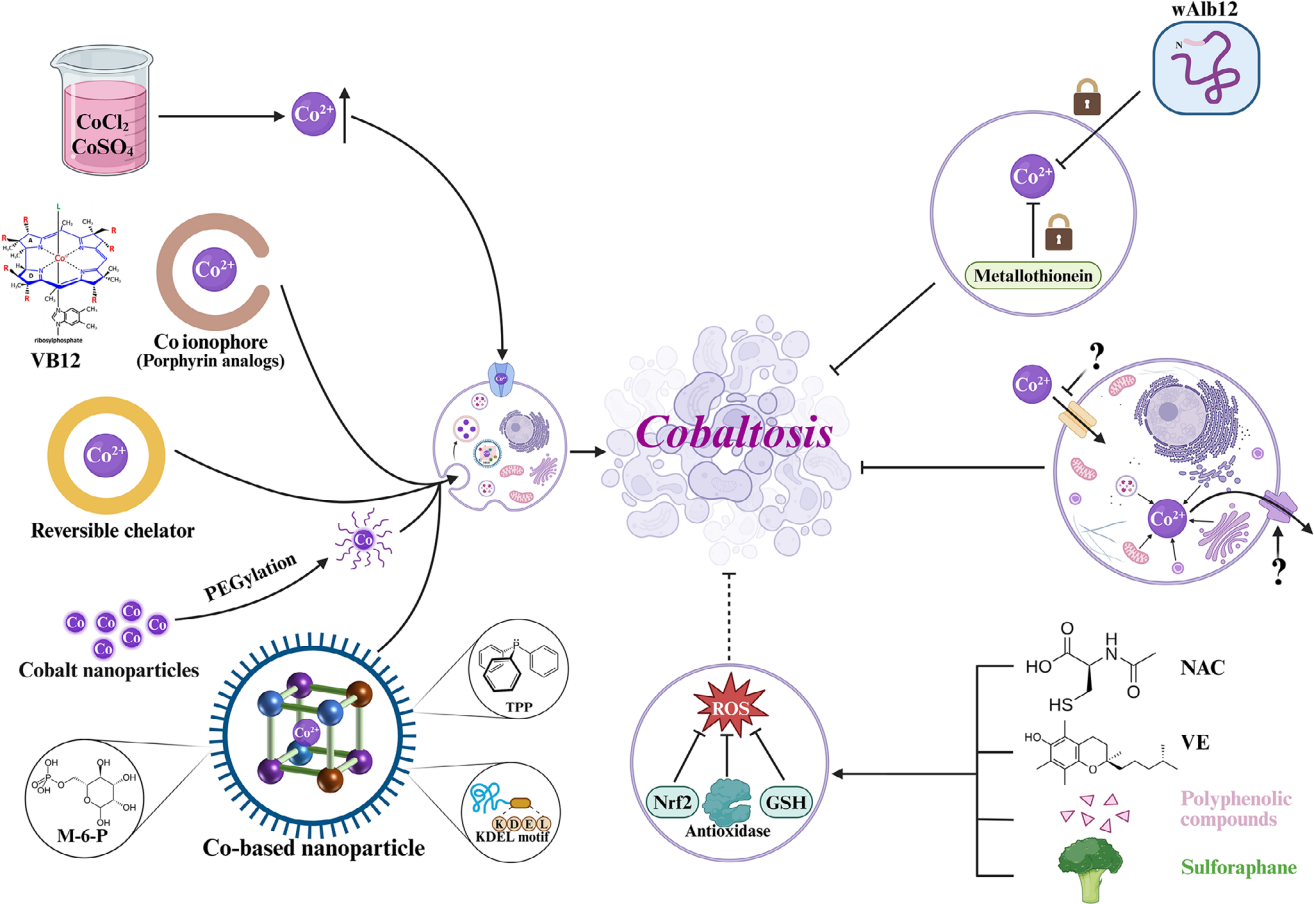
Methods to induce and inhibit cobaltosis.

Enhancing natural detoxification mechanisms is another potential method for mitigating cobaltosis. Metallothioneins, cysteine‐rich proteins that bind heavy metals, can sequester cobalt and reduce its harmful effects.^[^ ^277^ ^]^ The expression of metallothioneins can be induced by zinc supplementation or other environmental cues, bolstering the body's ability to detoxify cobalt. Additionally, activation of the Nrf2 pathway, which regulates the expression of antioxidant enzymes and detoxification proteins, may provide protection against cobalt toxicity. Compounds such as sulforaphane, derived from cruciferous vegetables, can activate Nrf2 and enhance cellular defenses^[^ ^278^ ^]^ (**Figure** **4**).

To discover novel and potentially highly specific inhibitors of cobaltosis, unbiased screening approaches can be employed. High‐throughput screening of diverse small molecule libraries, using cobalt‐induced cell death as the primary readout, could identify compounds that selectively rescue cells from cobaltosis. Finally, the validation of any putative cobaltosis inhibitor requires stringent controls. The inhibitor should ideally demonstrate dose‐dependent rescue from cobalt‐induced cell death. Crucially, it should not broadly inhibit other forms of RCD when tested against specific inducers of apoptosis, necroptosis, ferroptosis, or cuproptosis, unless a shared mechanistic node is intentionally targeted and characterized.

### 3.8 An Experimental Roadmap for Validating “Cobaltosis”

The foundational step is to identify direct and specific molecular targets of cobalt ions within the cell that could serve as the primary initiating sensors or triggers for a unique death cascade. While proteins are prime candidates, exploring cobalt's specific interactions with other classes of biomolecules, such as unique lipid species (beyond general peroxidation effects), specific RNA structures (e.g., cobalt‐responsive riboswitches, if any exist in eukaryotes), or particular metabolites whose alteration by cobalt might initiate a death signal, should also be considered.

To better understand the interactions between cobalt and biological macromolecules, spectroscopy techniques like X‐ray absorption spectroscopy (XAS) and electron paramagnetic resonance (EPR) are employed. These techniques provide information about the chemical speciation of cobalt in biological systems and its binding to proteins, DNA, or other cellular components, helping to elucidate the molecular mechanisms underlying cobalt toxicity.^[^ ^279^ ^]^

Imaging technologies also play a critical role. Fluorescence microscopy can visualize cobalt uptake and localization within cells, while more advanced imaging techniques, such as confocal microscopy and electron microscopy, allow for the examination of subcellular changes, including mitochondrial damage and autophagy. Owing to its high spatial resolution and unique compositional sensitivity, confocal Raman microscopy is used as a unique label‐free tool to trace drugs and nanoparticles within cells.^[^ ^280^, ^281^ ^]^ The interaction between cobalt metal nanoparticles and cancer cells was investigated using Raman spectroscopy as a label‐free tool.^[^ ^280^, ^282^, ^283^ ^]^

Genome‐wide CRISPR screens represent the gold standard for identifying genetic dependencies in cell death pathways. For cobaltosis, both positive and negative selection screens should be employed. Positive selection screens would identify genes whose knockout confers resistance to cobalt‐induced death, revealing essential mediators of the pathway. Negative selection screens could identify genes whose loss sensitizes cells to cobalt, potentially revealing natural resistance mechanisms. Complementary chemical genetic screens using diverse compound libraries could identify small molecules that specifically modulate cobaltosis without affecting other death pathways, providing both mechanistic insights and potential therapeutic leads.

Multi‐omics approaches are essential for capturing the systems‐level changes associated with cobaltosis. Transcriptomics using single‐cell RNA sequencing could reveal whether cobalt induces a unique gene expression signature distinct from other metal‐induced deaths. Particular attention should be paid to the temporal dynamics of gene expression to identify early response genes that might serve as specific biomarkers. Proteomics, especially post‐translational modification analyses, could reveal cobalt‐specific signaling cascades. Metabolomics presents a particularly powerful approach given the metabolic disruptions associated with metal toxicity. If cobaltosis targets specific metabolic pathways, unique metabolite signatures should emerge. Lipidomics could reveal whether cobalt induces specific lipid modifications distinct from the polyunsaturated fatty acid peroxidation characteristic of ferroptosis.

If a distinct molecular pathway for “cobaltosis” is delineated in vitro, the next crucial step is to investigate its relevance in vivo. This presents substantial challenges given the complexity of metal homeostasis in whole organisms. Initial studies should focus on model organisms with well‐characterized genetics, such as *C. elegans* and *Drosophila*, where tissue‐specific genetic manipulations can test the conservation of cobaltosis mechanisms. Mouse models with conditional knockout of putative cobaltosis mediators could reveal whether this pathway contributes to cobalt‐associated pathologies.

Table 1 provides a detailed comparative analysis of the three metal‐dependent cell death pathways discussed in this review. The table systematically compares mechanisms, detection methods, molecular markers, and clinical relevance.

However, validating “cobaltosis” as a distinct regulated cell death pathway requires moving from this conceptual framework to empirical evidence. This transition from hypothesis to validation is critically dependent on a sophisticated toolkit of advanced detection methods and a deep understanding of its potential crosstalk with known pathways, which we will explore next.

## 4 Crosstalk, Divergence, and the “Metal Death Code” in Transition Metal‐Dependent Cell Death

### 4.1 Comparative Analysis of Core Mechanisms

Reactive oxygen species are a common feature of cell death induced by redox‐active transition metals, yet their role ranges from being a direct and specific executioner to a secondary consequence or amplifier of cellular damage. In ferroptosis, ROS are not merely present; they are the central and direct effectors of the lethal event through a very specific process: iron‐dependent lipid peroxidation. The pathway execution hinges on the catalytic activity of redox‐active iron, primarily in the Fe^2^⁺ state within the labile iron pool (LIP), which drives Fenton chemistry to generate highly reactive radicals. These radicals initiate a self‐propagating chain reaction on polyunsaturated fatty acids (PUFAs) embedded within cellular membranes, leading to the overwhelming accumulation of specific lipid hydroperoxides (PLOOH). In stark contrast, the role of ROS in cuproptosis appears to be secondary. The primary insult is the direct binding of copper ions to lipoylated mitochondrial proteins, leading to their aggregation and a subsequent collapse of mitochondrial metabolism. While this profound mitochondrial dysfunction can certainly lead to increased production of mitochondrial ROS, it is largely considered a consequence of the primary proteotoxic event rather than its cause. This distinction is underscored by the observation that cuproptosis is not rescued by ferroptosis‐specific inhibitors like ferrostatin‐1. For the hypothetical “cobaltosis,” cobalt is well‐known to induce oxidative stress through Fenton‐like reactions (although less efficient than iron) and by depleting cellular antioxidants like glutathione.^[^ ^192^ ^]^ If cobaltosis is a distinct RCD, a critical question is whether its ROS signature is unique. Does it generate specific types of ROS, or does it rely on ROS from a particular subcellular compartment or enzymatic source not predominantly involved in ferroptosis or cuproptosis? Or, alternatively, could a putative cobaltosis be ROS‐independent, driven by a different primary toxic effect of cobalt, with ROS being merely an epiphenomenon?

Mitochondria are a central nexus for cellular metabolism, energy production, regulating metal storage and release, and cell death signaling, making them a common arena for all these metal‐dependent pathways. Mitophagy can liberate stored metals, potentially triggering cell death, while mitochondrial dynamics (fusion/fission) are regulated by both pathways.^[^ ^301^, ^302^ ^]^ However, the *mode and primacy* of mitochondrial involvement differ fundamentally. Cuproptosis represents the most direct mitochondrial assault, where copper specifically binds to lipoylated components of the TCA cycle enzymes DLAT and DLST, inducing their oligomerization and aggregation within the mitochondrial matrix. This protein aggregation leads to proteotoxic stress and acute metabolic collapse, with FDX1 playing a crucial role in reducing Cu^2^⁺ to the more reactive Cu^1^⁺ that drives protein aggregation. Ferroptosis, while also involving mitochondrial dysfunction, exhibits a fundamentally different mechanism where mitochondria serve more as amplifiers than initiators of death. The depletion of GPX4 activity leads to the accumulation of lipid peroxides in mitochondrial membranes, compromising membrane integrity and releasing mitochondrial ROS that propagate lipid peroxidation throughout the cell.^[^ ^303^, ^304^ ^]^ Voltage‐dependent anion channels (VDACs) have been implicated in ferroptosis, potentially serving as conduits for mitochondrial metabolite release, which exacerbates cellular dysfunction.^[^ ^36^ ^]^ Cobalt's mitochondrial effects appear more pleiotropic and involve direct inhibition of electron transport chain complexes, disruption of mitochondrial calcium homeostasis, and induction of the mitochondrial permeability transition pore. This multi‐target mitochondrial dysfunction resulting from cobalt exposure raises the question of whether cobaltosis might represent a unique form of mitochondrial stress that integrates features of both proteotoxic and oxidative damage.

Perhaps no molecule better exemplifies the convergent vulnerabilities exploited by metal‐dependent death pathways than GSH, which serves as a critical node linking oxidative stress, metal detoxification, and cell survival across all three pathways. In ferroptosis, GSH depletion through inhibition of the cystine/glutamate antiporter system xc^−^ represents the canonical trigger, as reduced GSH levels compromise GPX4 activity and permit lethal lipid peroxidation. Cuproptosis, while not primarily driven by GSH depletion, shows clear connections to glutathione metabolism, as copper ions can directly bind GSH, forming copper‐glutathione complexes that are exported from cells, effectively depleting both copper and GSH simultaneously.^[^ ^305^, ^306^ ^]^ This dual depletion creates a complex cellular state where reduced copper toxicity comes at the cost of compromised antioxidant defenses,^[^ ^307^ ^]^ potentially sensitizing cells to other stressors. For a hypothetical “cobaltosis,” cobalt is known to deplete GSH and inhibit GSH‐related enzymes, contributing to oxidative stress. A key question would be whether this GSH depletion is a central, regulated initiating event for a specific “cobaltotic” program (and if so, how it would differ from ferroptosis) or merely a consequence of general cobalt‐induced oxidative toxicity.

The disruption of metal homeostasis represents both a cause and a consequence of metal‐dependent cell death, with complex interconnections between different metal regulatory systems. In ferroptosis, the accumulation of redox‐active iron in the labile iron pool drives pathogenesis,^[^ ^69^ ^]^ but this accumulation often results from dysregulation of iron‐handling systems, including increased uptake (via transferrin receptor upregulation), decreased storage (through ferritinophagy), or impaired export (via ferroportin downregulation). Importantly, ferroptosis can be triggered without exogenous iron supplementation, instead resulting from the redistribution of existing cellular iron pools. Cuproptosis requires copper accumulation beyond the cell's substantial buffering capacity, typically necessitating exposure to copper ionophores or genetic defects in copper handling.^[^ ^107^ ^]^ The specificity of copper for lipoylated proteins suggests a more targeted disruption compared to iron's broader catalytic effects. Intriguingly, copper accumulation can secondarily affect iron metabolism by interfering with iron‐sulfur cluster assembly, potentially sensitizing cells to ferroptosis. Moreover, excess copper can displace zinc from numerous zinc finger proteins, potentially disrupting transcriptional responses that might otherwise protect cells. Copper accumulation can also interfere with iron‐sulfur cluster assembly in mitochondria, disrupting iron homeostasis.^[^ ^308^ ^]^ Calcium signaling is another critical molecular pathway shared by these forms of cell death.^[^ ^309^, ^310^ ^]^ Both cuproptosis and ferroptosis are associated with disruptions in calcium homeostasis. The proposed cobaltosis involves perhaps the most promiscuous metal dysregulation, with cobalt competing for binding sites and transporters used by iron, zinc, calcium, and other essential metals.^[^ ^180^ ^]^ This broad interference pattern raises fundamental questions about specificity—does cobaltosis represent a specific response to cobalt or a general consequence of multi‐metal dyshomeostasis?

**Figure 5:**
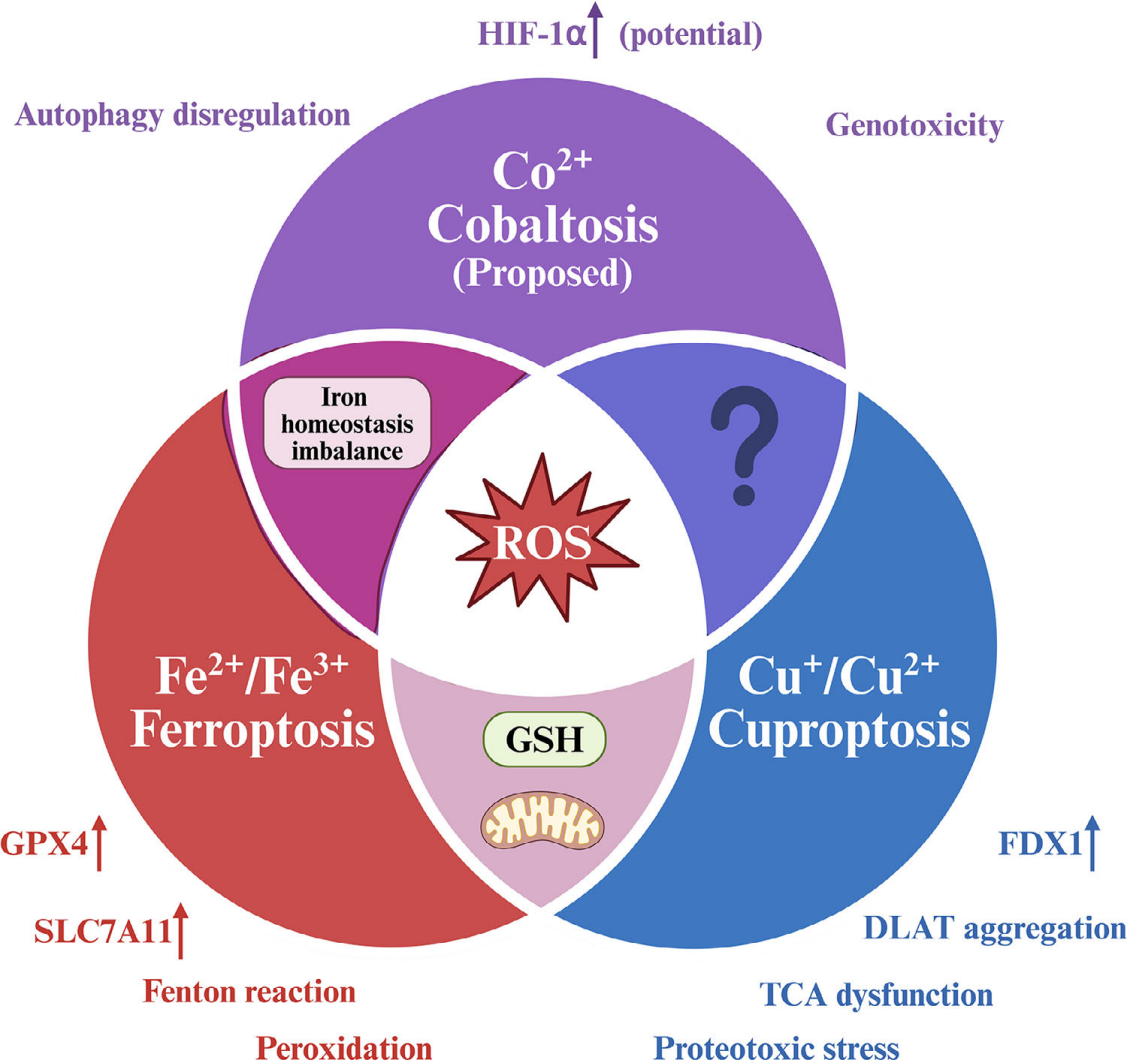
Crosstalk and divergence among metal‐dependent cell death pathways: ferroptosis, cuproptosis, and cobaltosis.

The complex interplay between ferroptosis, cuproptosis, and the hypothetical cobaltosis is illustrated in **Figure** **5**.

### 4.2 The “Metal Death Code”: Cellular Decision‐Making in Response to Transition Metal Stress

The existence of multiple, mechanistically distinct RCD pathways triggered by different transition metals raises a fundamental and fascinating question in cell biology: how does a cell “decide” which fate to embrace when confronted with the dyshomeostasis of one or more of these essential yet potentially toxic elements? This suggests the operation of a sophisticated and dynamic cellular information processing system, a conceptual framework we term the “metal death code.” This code is envisioned not as a simple, linear pathway but as a complex, integrated regulatory network that senses the specific nature of the metallic threat, processes this information in the context of the cell's current physiological and metabolic state, and ultimately dictates a specific outcome, which could range from adaptation and survival to commitment to a particular RCD program.

The foundation of the “metal death code” lies in the cell's ability to sense perturbations in its metallome and integrate these signals with other cellular stress pathways. At the frontline, metal‐responsive transcription factors serve as primary sensors.^[^ ^311^, ^312^ ^]^ However, these classical sensors primarily regulate homeostatic responses rather than death decisions. For ferroptosis, sensing is multi‐layered, involving the perception of depleted GSH and inactivated GPX4, the accumulation of labile iron (influenced by TfR1, NCOA4‐mediated ferritinophagy, and FPN1), and the direct consequences of lipid peroxidation on membrane integrity. For cuproptosis, the “sensing” mechanism appears to be more direct and localized: once homeostatic systems are overwhelmed and excess copper accumulates in the mitochondria, the direct binding of copper ions to the lipoic acid moieties of TCA cycle proteins like DLAT and DLST acts as the critical initiating event. Therefore, the availability of these lipoylated mitochondrial targets, coupled with the activity of upstream regulators like FDX1, constitutes the specific sensing module for cuproptosis induction. For a hypothetical “cobaltosis,” the specific cobalt sensor remains elusive; it could potentially involve proteins that bind cobalt with high affinity and specificity, leading to a unique conformational change or functional alteration that triggers a dedicated cascade, or perhaps a more complex integration of cobalt's known effects, like HIF‐1α stabilization with other currently unappreciated cobalt‐specific interactions.

The selection between different metal‐dependent death pathways depends on multiple cellular parameters that create a multidimensional decision space. Metal ion identity, concentration, and subcellular localization are the most critical determinants. The distinct chemical properties of iron, copper, and cobalt dictate their preferred molecular targets. The specific intracellular concentration thresholds required to trigger each pathway are likely different, and where the metal accumulates is paramount. For example, a significant increase in the cytosolic and membrane‐associated LIP is a prerequisite for ferroptosis, whereas a critical accumulation of copper within the mitochondrial matrix is necessary to initiate cuproptosis. The cell type and its specific metabolic state are key determinants. For instance, cells with high levels of PUFAs in their membranes, high rates of glutaminolysis, and a reliance on the GPX4 system are inherently more susceptible to ferroptosis. Similarly, cells with high mitochondrial respiratory activity and abundant lipoylated proteins or high FDX1 expression may be particularly vulnerable to cuproptosis. The expression levels of specific metal transporters, chaperones, and detoxification systems (e.g., metallothioneins) will significantly modulate intracellular metal availability and thus pathway engagement.

**Figure 6:**
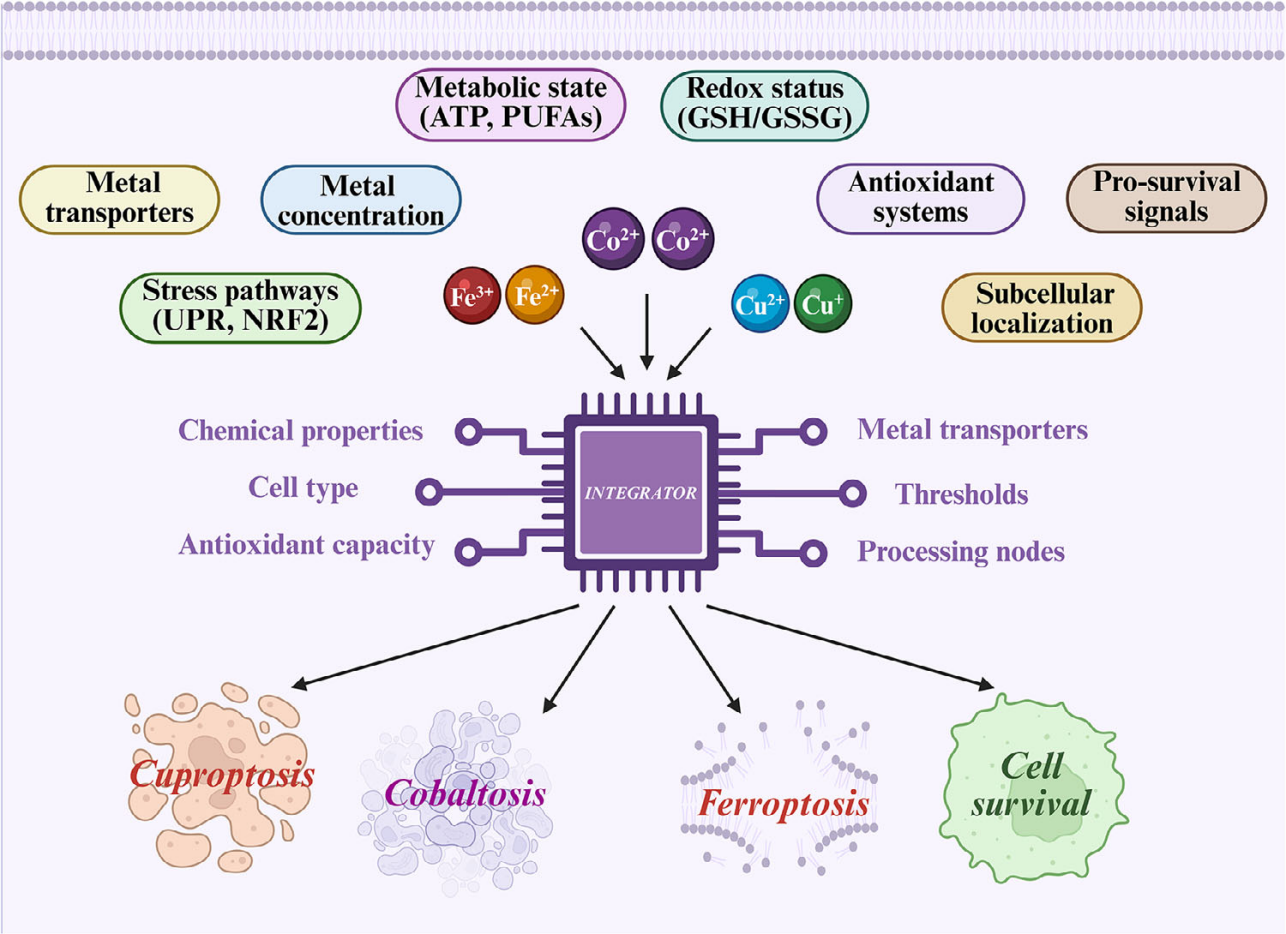
Schematic of “metal‐death integrator”.

As the “Metal Death Code” was gradually deciphered, we can conceptualize a “metal‐death integrator” (**Figure** **6**) as a hypothetical cellular decision‐making module or network that processes various inputs related to metal stress and outputs a decision to activate a specific RCD pathway or an alternative survival/stress response. This integrator wouldn't necessarily be a single molecule but rather interconnected signaling networks. It should consist of at least the following plates:

Inputs: Metal type, concentration, subcellular localization; levels of specific metal‐binding proteins and chaperones; redox status (GSH/GSSG ratio, NADPH levels, ROS levels); status of antioxidant systems (GPX4, SODs, catalase); metabolic state (ATP levels, PUFA availability, TCA cycle flux); activity of general stress pathways (UPR, DDR, NRF2); presence of pro‐survival signals.

Processing Nodes: Key regulatory molecules within each potential RCD pathway (e.g., GPX4 for ferroptosis, FDX1/lipoylated proteins for cuproptosis, caspases for apoptosis). These nodes would be subject to regulation by the input signals. Crosstalk points between pathways (e.g., shared upstream regulators, competition for substrates or cofactors) would also act as processing nodes.

Thresholds: Activation of a specific RCD pathway might require certain input signals to cross specific thresholds. For example, ferroptosis might be triggered only when GPX4 activity falls below a critical level and sufficient PUFAs are available for peroxidation.

Outputs: Activation of a dominant RCD pathway (e.g., ferroptosis, cuproptosis, apoptosis, or a putative “cobaltosis”), or activation of adaptive/survival responses if the stress is sub‐lethal. The system might also allow for sequential activation or hierarchical engagement of pathways.

From an evolutionary perspective, ferroptosis appears ancient, with GPX4 homologs present in bacteria, suggesting that early life forms require defenses against iron‐catalyzed lipid peroxidation.^[^ ^313^, ^314^ ^]^ The conservation of metal death pathways across species supports their physiological importance. Ferroptosis‐like mechanisms exist in plants and fungi, using ferroptosis‐like pathways for pathogen resistance or response to heat stress.^[^ ^315^, ^316^, ^317^, ^318^ ^]^ The evolution of cuproptosis might be linked to the “Great Oxidation Event” (∼2.4 billion years ago), when rising oxygen levels in the atmosphere made soluble, toxic copper ions (Cu⁺/Cu^2^⁺) more bioavailable, creating strong selective pressure for cells to develop mechanisms to cope with copper toxicity.^[^ ^319^, ^320^ ^]^ The TCA cycle and mitochondrial respiration are ancient metabolic pathways. It is plausible that cuproptosis evolved as a fail‐safe mechanism to eliminate cells whose core mitochondrial metabolic mechanism has been irrevocably compromised by copper, thereby preventing the proliferation of metabolically dysfunctional cells. Its specificity for lipoylated proteins of the TCA cycle suggests a highly targeted response to damage within this central metabolic hub.

The “Metal Death Code” is therefore not a static rulebook but a dynamic system shaped by evolutionary pressures, cellular context, and the specific chemical challenges posed by different transition metals. Understanding this code is crucial for deciphering disease pathogenesis where metal dyshomeostasis is implicated and for developing targeted therapeutic strategies that can selectively induce or inhibit these death pathways. Deciphering this code is a central challenge that demands more than just conceptual models. Meeting this challenge is impossible without leveraging the cutting‐edge research methods and technologies that allow for the dissection of these pathways at unprecedented resolution, as we will now discuss.

## 5 Advanced Research Methods for Dissecting Metal‐Dependent Regulated Cell Death

### 5.1 Advanced Imaging and Detection Methods

Visualizing the dynamic and often subtle cellular events that underpin RCD is paramount. Advanced imaging and detection techniques offer unprecedented spatial and temporal resolution, allowing researchers to observe metal ion fluxes, organellar changes, protein interactions, and the generation of specific death signals in real‐time and with molecular specificity. In cuproptosis, TEM can reveal mitochondrial enlargement, crista disruption, and aggregation of mitochondrial proteins,^[^ ^290^ ^]^ Scanning electron microscopy (SEM) offers surface morphological details, useful for observing cellular membrane alterations.^[^ ^321^ ^]^ Super‐resolution microscopy provides detailed structural insights at the nanoscale level.^[^ ^322^ ^]^ Confocal laser scanning microscopy, combined with specific fluorescent probes, allows real‐time imaging of copper and cobalt ions within cellular compartments.^[^ ^323^ ^]^ For cobalt, carbon dots (CDs), polymer dots (PDs), boron and nitrogen‐doped carbon quantum dots (B, N‐CQDs), and Tokyo Green derivative‐based cobalt probe (CP1) offer fluorescence enhancement upon Co^2^⁺ binding, exhibiting remarkable selectivity and enabling the cellular imaging of Co^2^⁺ levels in cells.^[^ ^291^, ^292^, ^293^, ^294^ ^]^

Given that RCDs are fundamentally driven by specific metals, the ability to directly visualize, quantify, and determine the chemical state of these metals at subcellular resolution is critical. Advanced elemental mapping and speciation techniques provide these crucial insights. Laser ablation inductively coupled plasma mass spectrometry (LA‐ICP‐MS) allows spatial mapping of metal ions in biological samples. This technique has been employed to visualize the distribution of copper and cobalt at cellular and tissue levels, providing quantitative data on metal accumulation associated with toxicity.^[^ ^324^ ^]^ Synchrotron radiation‐based X‐ray fluorescence (SR‐XRF) microscopy offers high‐resolution elemental mapping of metal ions in biological specimens. This method provides insights into metal localization and speciation, critical for elucidating metal‐induced cellular damage.^[^ ^325^, ^326^ ^]^ X‐ray absorption spectroscopy (XAS), including X‐ray absorption near‐edge structure (XANES) and extended X‐ray absorption fine structure (EXAFS), enables analysis of the oxidation states and coordination environments of metals in biological systems.^[^ ^327^ ^]^ Electron paramagnetic resonance (EPR) spectroscopy detects paramagnetic species like Cu^2^⁺ and Co^2^⁺, providing information on metal‐protein interactions and redox states.^[^ ^328^ ^]^

Genetically encoded biosensors have been developed to monitor cellular redox states. For instance, roGFP probes detect changes in GSH redox potential, providing insights into oxidative stress during cuproptosis and cobaltosis.^[^ ^329^ ^]^ MitoSOX Red is used to measure mitochondrial superoxide production, indicating mitochondrial dysfunction.^[^ ^162^ ^]^ The development of FRET‐based metal sensors has enabled the detection of metal‐protein interactions: the FRET pair GPX4‐mCherry/GFP‐ACSL4 reveals the spatial relationship between these ferroptosis regulators.^[^ ^330^, ^331^ ^]^

### 5.2 Molecular and Genetic Approaches

Unraveling the complex gene regulatory networks, protein interactions, and metabolic reprogramming that govern metal‐dependent RCDs requires sophisticated molecular and genetic tools.

High‐throughput transcriptomic technologies like RNA sequencing provide comprehensive overviews of global changes in gene expression. Proteomic and metabolomic analyses offer comprehensive insights. Mass spectrometry‐based proteomics identifies proteins differentially expressed or modified during cuproptosis, including lipoylated protein aggregates.^[^ ^332^ ^]^ Metabolomic profiling using liquid chromatography–mass spectrometry (LC–MS) reveals alterations in TCA cycle metabolites, highlighting metabolic disruptions.^[^ ^333^ ^]^

Genetic manipulation techniques, such as RNA interference (RNAi) and CRISPR‐Cas9 gene editing, enable the selective knockdown or knockout of target genes to assess their contribution to metal‐induced toxicity.^[^ ^334^ ^]^ For example, knocking down genes involved in copper transport, like CTR1, reduces copper uptake and mitigates toxicity.^[^ ^335^ ^]^ CRISPR‐Cas9 genome editing allows the creation of cell lines and animal models with specific genetic alterations relevant to cuproptosis and cobaltosis, facilitating the investigation of genes implicated in copper homeostasis disorders. Functional genomics screens using CRISPR libraries have uncovered novel regulators of metal homeostasis and cell death pathways, providing new targets for therapeutic intervention.^[^ ^336^ ^]^ Unbiased CRISPR‐based positive selection screens identified selenium metabolism genes as critical ferroptosis regulators, revealing that ferroptosis is actuated through simultaneous reduction of cellular selenium and increased membrane lipid oxidation.^[^ ^337^ ^]^ Genome‐wide CRISPRi/CRISPRa screens identified ferroptosis‐specific vulnerabilities through the CRISPRbrain data commons platform. The screens revealed that prosaposin (PSAP) knockdown triggers lipofuscin formation, leading to iron accumulation and ferroptosis, providing new therapeutic targets for neurodegenerative diseases.^[^ ^338^ ^]^

Advanced genomic technologies, including genome‐wide association studies (GWASs) and functional genomics screens, have further expanded our understanding of genetic factors that influence susceptibility to cuproptosis and cobaltosis. GWASs have identified single‐nucleotide polymorphisms (SNPs) in genes related to metal metabolism and oxidative stress response that are associated with an increased risk of metal‐induced toxicity.^[^ ^339^ ^]^ Epigenetic studies, including DNA methylation profiling and chromatin immunoprecipitation sequencing (ChIP‐seq), reveal how metal ions regulate gene expression at the epigenetic level, affecting cellular responses to stress.^[^ ^340^ ^]^ Metabolomic and systems biology approaches integrate metabolomic data with transcriptomic and proteomic information, providing a holistic understanding of metabolic networks affected by metal toxicity.^[^ ^333^ ^]^ Single‐cell RNA sequencing (scRNA‐seq) allows analysis of gene expression at the individual cell level, uncovering cellular heterogeneity in response to metal exposure.^[^ ^341^ ^]^ Additionally, microRNAs and other non‐coding RNAs play significant roles in regulating gene expression post‐transcriptionally, with metal exposure altering specific miRNAs that modulate metal‐induced toxicity.^[^ ^342^ ^]^ These molecular and genetic approaches not only advance fundamental knowledge but also have the potential to inform the development of therapies for conditions associated with copper and cobalt dysregulation.

### 5.3 Nanotechnology and Drug Delivery Systems

While conventional small‐molecule inhibitors and inducers are invaluable, nanotechnology and advanced delivery systems have emerged as powerful research platforms for dissecting the molecular mechanisms of ferroptosis, cuproptosis, and other forms of “metalloptosis.”^[^ ^343^ ^]^ These engineered nanomaterials (NMs) offer unparalleled advantages in their ability to precisely deliver metal ions or pathway modulators to specific subcellular locations, achieve controlled release kinetics, and manipulate the cellular microenvironment.

A primary application of nanotechnology in this field is for the controlled and targeted delivery of metal ions. For instance, to investigate the specific consequences of iron overload in ferroptosis, various iron‐containing nanoparticles, such as ultrasmall superparamagnetic iron oxide nanoparticles.^[^ ^344^ ^]^ In addition to direct metal delivery, nanotechnology offers sophisticated tools to indirectly modulate cellular metabolism and signaling pathways. Copper‐based MOFs have been utilized as carriers to deliver copper ions into cancer cells. MOFs such as [Cu(tz)] (where tz = 1,2,4‐triazole) provide a porous scaffold that can encapsulate therapeutic agents such as glucose oxidase (GOx). In the tumor microenvironment (TME), overexpressed GSH triggers the disassembly of the MOF, releasing copper ions and Gox and enhancing cuproptosis.^[^ ^345^ ^]^ Similar strategies involve the use of nanoparticles to deliver cobalt chelators or antioxidants to mitigate oxidative stress. Cobalt‐based nanomaterials have also been investigated for their potential to induce cobaltosis and serve as therapeutic agents. For example, cobalt‐doped zinc imidazole frameworks (Co/ZIF‐8/ICG/Pt) have been developed to achieve synergistic CDT and enhanced PDT. Under near‐infrared (NIR) irradiation, photosensitizers such as indocyanine green (ICG) generate singlet oxygen (^1^O_2_).^[^ ^346^ ^]^

Nanomaterials can also directly interact with metal ions to modulate their availability and toxicity. MOFs and nanochelators are engineered to capture and sequester excess copper or cobalt ions within their porous structures. These nanomaterials act as sponges, reducing free metal ion concentrations in the cellular environment.^[^ ^347^ ^]^

Nanoparticles can be engineered to deliver metal chelators, antioxidants, or inhibitors of cell death pathways directly to cells experiencing metal‐induced stress. This targeted delivery enhances the drug concentration at the site of action while minimizing systemic exposure and adverse effects.^[^ ^348^ ^]^ For cuproptosis, nanoparticles can be designed to deliver copper chelators such as tetrathiomolybdate to cells with excessive copper accumulation. By encapsulating these chelators within biocompatible nanocarriers—such as liposomes, polymeric nanoparticles, or dendrimers—the drugs can be protected from degradation, exhibit prolonged circulation times, and achieve sustained release. Surface modification of nanoparticles with ligands or antibodies that recognize cell‐specific markers allows for active targeting of cells susceptible to cuproptosis. In cuproptosis, targeting mitochondria is crucial since the pathway involves mitochondrial dysfunction. Mitochondria‐targeting sequences (MTS) or TPP cations can be attached to nanoparticles to facilitate mitochondrial delivery.^[^ ^349^ ^]^ Expanding beyond mitochondria, nanomaterials can be engineered to target other organelles, such as lysosomes, ER, and the Golgi apparatus, to induce cell death through alternative pathways.

Nanoparticles can also be functionalized with surface coatings or ligands to enhance their biocompatibility, targeting specificity, and stability in biological fluids. One of the challenges is overcoming biological barriers that limit the distribution and effectiveness of therapeutics, such as the reticuloendothelial system (RES) and, in cases of neurotoxicity, the BBB. Surface modification with PEG, reduces nanoparticle recognition and clearance by the RES, prolonging circulation time and increasing the likelihood of reaching target tissues.^[^ ^350^ ^]^ For neuroprotective applications, nanoparticles can be functionalized with ligands that facilitate BBB crossing, such as transferrin or lactoferrin, ensuring delivery of therapeutic agents to the central nervous system.^[^ ^351^ ^]^ Comprehensive in vitro and in vivo evaluations are essential to assess the long‐term effects of nanoparticle administration, ensuring that they do not induce unintended toxicity or interfere with normal physiological processes.^[^ ^352^ ^]^

Furthermore, nanotechnology provides versatile platforms for the delivery of genetic material, such as siRNA, shRNA, miRNA mimics/inhibitors, or CRISPR‐Cas9 components, to precisely modulate the expression of specific genes involved in metal homeostasis or RCD regulation. Lipid nanoparticles (LNPs), polymeric nanoparticles, and other nano‐constructs can be used to efficiently deliver these genetic payloads into cells in vitro and, with increasing sophistication, in vivo.^[^ ^353^, ^354^, ^355^, ^356^ ^]^

### 5.4 Integration of Advanced Technologies

The true power of modern approaches to studying metal‐dependent cell death lies not in individual technologies but in their integration to provide systems‐level insights. The convergence of advanced imaging, omics technologies, nanotechnology, and computational modeling has begun to reveal the full complexity of how cells integrate metal status with metabolic state, stress responses, and death decisions.

Techniques such as ICP‐MS allow for precise quantification of metal ions at trace levels, providing insights into their distribution and concentration in biological samples.^[^ ^357^ ^]^ Advanced imaging methods enable high‐resolution mapping of metal ions at the cellular and subcellular levels.^[^ ^358^ ^]^ This spatial information is crucial for correlating metal localization with cellular structures and functions.

The advent of omics technologies, including genomics, transcriptomics, proteomics, metabolomics, and metallomics, has revolutionized the ability to study biological systems at a systems level. Integrating data from these platforms through systems biology approaches allows for the construction of complex models that describe the interactions between metals and various cellular components.^[^ ^359^, ^360^, ^361^ ^]^ Computational tools and bioinformatics are essential for managing and analyzing the vast amounts of data generated. Machine learning and artificial intelligence algorithms can identify patterns and predictive markers within multidimensional datasets, facilitating the discovery of novel insights into metal‐induced cell death mechanisms.^[^ ^362^, ^363^, ^364^ ^]^ OmicsNet 2.0 and MetaboAnalyst 5.0 represent cutting‐edge platforms supporting eight different data types with enhanced visualization.^[^ ^365^, ^366^ ^]^ CellDeathPred achieves 95% accuracy in distinguishing ferroptosis from apoptosis using label‐free cell painting microscopy with an EfficientNet‐b0 architecture.^[^ ^171^ ^]^ Mathematical models of ferroptosis using 73 differential equations and 93 species to predict death sensitivity based on metallome parameters, revealing that cell death sensitivity depends on combinatorial metal inputs.^[^ ^367^ ^]^

High‐throughput screening platforms and microfluidic technologies contribute to the integration of advanced technologies by enabling large‐scale experimentation and analysis. Microfluidic devices can create controlled environments for studying cell responses to metal exposure under precisely defined conditions.^[^ ^368^, ^369^ ^]^ These platforms facilitate the screening of compounds that modulate metal homeostasis or protect against metal‐induced toxicity, accelerating the identification of potential therapeutics.

Integration of computational modeling with experimental data is crucial for predicting the behavior of complex biological systems influenced by metal ions. In silico models can simulate metal binding kinetics, transport dynamics, and their effects on cellular processes.^[^ ^370^ ^]^ In the context of therapeutic development, the integration of advanced technologies facilitates the identification of novel drug targets and the design of effective treatment strategies. Computational approaches aid in drug discovery by modeling the interactions between potential therapeutic compounds and metal‐regulatory proteins.^[^ ^371^ ^]^ Deep learning screening of 6 million compounds identified LGOd1 as a novel cuproptosis inducer targeting hepatocellular carcinoma through copper homeostasis interference.^[^ ^372^ ^]^ Nanotechnology‐based drug delivery systems can improve the targeting and efficacy of these therapeutics, reducing side effects and enhancing patient outcomes.

Interdisciplinary collaboration is a cornerstone of integrating advanced technologies. Chemists, biologists, physicists, and computational scientists must work together to develop and apply these methods effectively. For instance, the design of metal‐specific probes requires expertise in chemistry and materials science, while their application in biological systems necessitates knowledge in cell biology and microscopy.

The advanced technologies discussed in this section are summarized in **Table** **2**.

**Table 2:** Advanced technologies for studying transition metal‐dependent cell death.

Technologies/Methods	Description	Applications	Advantages
Imaging and Detection Methods			
High‐Resolution Microscopy^[^ ^321^, ^323^ ^]^ (TEM, SEM, Confocal)	Nanometer to micrometer scale imaging; confocal offers 3D live‐cell imaging.	Visualize mitochondrial damage, protein aggregation, spatially resolve metal ion localization in organelles.	High resolution; detailed visualization; live‐cell imaging.
Super‐Resolution Microscopy^[^ ^322^ ^]^ (STED, PALM)	Nanometer‐scale resolution beyond diffraction limit.	Visualize nanoscale structures like protein aggregates and mitochondrial cristae; study subcellular localization of metals.	Ultra‐high resolution; detailed imaging.
FRET Imaging^[^ ^331^ ^]^	Detects molecular interactions via energy transfer between fluorescent probes.	Study protein‐protein interactions; monitor metal ion binding and protein conformational changes in real‐time.	Sensitive detection; real‐time monitoring in live cells.
Live‐Cell Imaging and Time‐Lapse Microscopy^[^ ^373^ ^]^	Real‐time observation of living cells over time.	Monitor the progression of cell death; observe dynamic processes in response to metal exposure.	Captures dynamic events; non‐invasive.
Multiphoton Microscopy^[^ ^374^ ^]^	Deep tissue imaging using near‐infrared light.	Image metal ion distribution in thick tissues or live animal models; in vivo studies of organ‐specific effects of metal toxicity.	Deep imaging; reduced phototoxicity.
Atomic Force Microscopy (AFM)^[^ ^375^ ^]^	Measures surface topography and mechanical properties.	Assess changes in cell membrane stiffness; detect nanoscale structural alterations.	High‐resolution imaging; mechanical measurements.
Mass Spectrometry Imaging (MSI)^[^ ^376^ ^]^	Spatially resolved chemical composition mapping.	Map distribution of metal ions in tissues; identify metal‐associated biomolecules; study metabolic changes.	High specificity; label‐free detection.
ICP‐MS and Laser Ablation ICP‐MS^[^ ^324^ ^]^	Quantifies metal ions with high sensitivity; spatial analysis via laser ablation.	Measure trace levels and map the distribution of metal; assess metal accumulation.	Extremely sensitive; quantitative; spatial resolution.
Synchrotron X‐ray Fluorescence Microscopy^[^ ^325^, ^326^ ^]^	high‐resolution elemental mapping and reveals oxidation states.	Visualize intracellular localization of metal ions; study co‐localization with organelles; investigate metal‐protein interactions.	Non‐destructive; high elemental sensitivity.
Fluorescent Biosensors and Bioluminescence Imaging^[^ ^377^ ^]^	Probes emitting light in response to specific molecules or conditions.	Detect intracellular metal levels; monitor ROS, redox states, and mitochondrial function; assess oxidative stress.	Real‐time monitoring; high sensitivity.
High‐Content Screening and Automated Analysis^[^ ^378^ ^]^	Automated microscopy with image analysis for high‐throughput studies.	Screen for compounds that modulate cuproptosis or cobaltosis; quantify phenotypic changes.	High‐throughput; provides quantitative, multi‐parameter data; accelerates discovery.
Molecular and Genetic Approaches			
Gene Expression Analysis (qRT‐PCR, RNA‐seq)	Quantifies mRNA levels; RNA‐seq for whole‐transcriptome analysis.	Identify genes affected during cuproptosis and cobaltosis; study stress response pathways; uncover novel regulatory biomarkers.	Sensitive; high‐throughput analysis.
Proteomics with PTM Analysis^[^ ^379^ ^]^	Large‐scale protein study; includes post‐translational modifications.	Identify protein aggregates; map signaling pathways; study PTMs induced by metal exposure; understand functional changes.	Comprehensive protein analysis; insights into function.
Genetic Manipulation (RNAi, CRISPR‐Cas9)^[^ ^380^ ^]^	Gene knockdown or editing techniques.	Functionally validate the roles of specific genes in metal toxicity; create cell and animal models.	Precise gene targeting for functional studies; establish causal relationships.
Epigenetic Studies (ChIP‐seq, Methylation Profiling)^[^ ^381^ ^]^	Examines DNA‐protein interactions and epigenetic modifications.	Investigate epigenetic changes due to metal exposure; understand regulation of toxicity‐related genes; identifying epigenetic biomarkers.	Insights into gene regulation; potential targets.
GWAS and Functional Genomics^[^ ^339^ ^]^	Identifies genetic variations; genome‐wide screens to identify genes that regulate biological process; validates gene functions.	Discover genetic factors influencing susceptibility to metal toxicity; identify risk alleles; guide personalized medicine approaches.	Identifies risk factors; unbiased, genome‐wide approach for gene discovery.
Metabolomics (NMR, LC‐MS)^[^ ^333^ ^]^	Analyzes metabolites; models metabolic pathways.	Uncover global molecular changes; identify biomarkers; understand complex interactions in metal‐induced toxicity.	Holistic view of metabolism; novel targets.
Single‐Cell Sequencing^[^ ^382^ ^]^	Analyzes gene expression of thousands of individual cells simultaneously.	Uncover cellular heterogeneity in response to metal exposure; identify rare or resistant cell populations.	High‐resolution data reveals cell‐to‐cell variability; understands complex tissue responses.
Heterogeneity Studies (Spatial Transcriptomics)^[^ ^383^ ^]^	Investigates variability among cells or tissues.	Understand differences in cellular responses; identify subpopulations susceptible or resistant to metal toxicity; map tissue heterogeneity.	Reveals complexity; informs targeted interventions.
High‐Throughput Screening & Microfluidics^[^ ^384^ ^]^	Rapid testing using microfluidic devices and platforms.	Screen large libraries of compounds; study cell responses to metals; develop drug candidates that modulate toxicity pathways.	Efficient; precise control; scalable.
Nanotechnology and Drug Delivery Systems			
Nanoparticles as Drug Carriers^[^ ^385^, ^386^ ^]^	Engineered nanomaterials that deliver therapeutic agents or metal ions to specific sites.	Targeted delivery of chelators, drugs, or genes to induce or inhibit cobaltosis selectively; improve treatment efficacy and safety.	Enhanced targeting; controlled release; reduced toxicity.
Organelles‐Targeting Nanocarriers^[^ ^387^ ^]^	Nanoparticles targeting mitochondria, lysosomes, etc.	Deliver metal ions or drugs directly to organelles; enhance specificity of toxicity induction.	Increases local drug concentration and specificity; potentiates desired effects.
Nanochelators and Metal Ion Modulators^[^ ^388^ ^]^	Sequester or modulate metal ions within cells.	Reduce excess metal ions to prevent toxicity; increase metal ions in targeted cells to induce cell death; modulate redox balance.	Controlled ion levels; customizable properties.
Integration of Advanced Technologies			
Combining Imaging and Molecular Analyses^[^ ^389^ ^]^	Integrates imaging with genomic, proteomic data.	Correlate structural changes with molecular pathways; enhance understanding of metal‐induced toxicity mechanisms; identify biomarkers.	Comprehensive insights; multidimensional analysis.
Nanotechnology with Genetic Approaches^[^ ^353^, ^354^, ^355^, ^356^ ^]^	Nanoparticles delivering genetic material for modulation.	Gene editing to modulate metal transporters or toxicity pathways; targeted gene therapy; enhancing therapeutic specificity.	Precise intervention; synergistic effects.
Computational Modeling and AI^[^ ^390^ ^]^	Uses machine learning and AI algorithms to analyze multi‐omics datasets and predict biological outcomes.	Predict cellular responses to metal exposure and drug sensitivity; discover novel drug candidates and biomarkers from large datasets; model toxicity pathways.	Handles large data; accelerates discovery and hypothesis generation.
Omics Technologies & Systems Biology^[^ ^391^ ^]^	Integrates genomics, proteomics, metabolomics data.	Identifying metabolic alterations during metal‐induced cell death; mapping metabolic pathways; understanding energy metabolism shifts.	Comprehensive data; systems‐level insights into pathway crosstalk.
Interdisciplinary Collaboration	Integrates expertise across multiple fields.	Advancing research; developing innovative solutions; clinical translation.	Accelerates innovation; diverse perspectives.

## 6 Translational Potential, Clinical Outlook, and Future Challenges in Targeting Metal‐Dependent Regulated Cell Death

The elucidation of distinct transition metal‐dependent regulated cell death (RCD) pathways—notably ferroptosis, cuproptosis, and the broader conceptual framework of “metalloptosis”—has transitioned from a realm of fundamental biological discovery to a vibrant frontier of translational research. The intricate involvement of these pathways in the onset, progression, and potential resolution of a wide spectrum of human diseases presents unprecedented opportunities for novel therapeutic interventions.

The core therapeutic strategy emerging from this field is the precise modulation of cellular metal content to either induce or inhibit a specific RCD. For cancer therapy, the focus is on developing metal‐enhancing agents or pathway inducers that can selectively eliminate pathological cells. For instance, advanced nanotechnology platforms are being engineered to achieve the targeted delivery of iron‐containing agents, such as ultrasmall iron oxide nanoparticles, to elevate the LIP and catalyze Fenton reactions, thereby triggering ferroptosis specifically within the tumor microenvironment.^[^ ^392^, ^393^, ^394^ ^]^ The use of nanomaterials rationally engineered to treat cancer is a burgeoning field that has reported great medical achievements.^[^ ^395^ ^]^ Similarly, exploiting the copper‐avid nature of many tumors, novel copper ionophores or copper‐based nanomaterials are being designed to induce cuproptosis. Recent advances include copper‐based nanomedicines that can induce cuproptosis in cancer both in vitro and in vivo.^[^ ^132^ ^]^ A key therapeutic concept is to combine these metal‐enhancing agents with drugs that cripple cellular defense mechanisms—such as inhibitors of GPX4 or system Xc^−^ for ferroptosis—to create a synthetic lethal therapeutic window and overcome resistance.

Conversely, for cytoprotection in neurodegenerative diseases such as Parkinson's disease or Alzheimer's disease, or in acute conditions like ischemia‐reperfusion injury, the focus is on developing strategies to inhibit pathological cell death. This includes the creation of highly specific, blood‐brain barrier‐penetrant metal‐chelating agents designed to sequester redox‐active iron or copper in affected tissues. Ferroptosis inhibitors, including ferrostatin‐1, liproxstatin‐1, and vitamin E, hold promise for mitigating tissue damage in conditions like IRI and neurodegeneration.^[^ ^101^, ^296^ ^]^ And dysregulation of copper homeostasis has been observed in a wide spectrum of neurological, fibrotic pulmonary, and vascular diseases as well as in different types of cancers. Under these conditions, copper chelation should be ideally able to restore ionic balance by precise modulation of copper homeostasis, but further randomized clinical trials are necessary to confirm the benefit observed in preclinical models.^[^ ^396^ ^]^ A crucial aspect of these cytoprotective strategies is mitigating metal‐related toxicities from long‐term treatment. This requires developing agents with a wide therapeutic index and carefully considering the potential systemic effects on essential physiological processes that rely on these metals.

A cornerstone of translating these therapies is the parallel development of metal‐specific imaging and biosensing technologies to serve as diagnostic, prognostic, and pharmacodynamic tools. To realize the vision of precision medicine, we must be able to stratify patients based on their metal dysregulation status. Non‐invasive imaging techniques such as quantitative susceptibility mapping (QSM) via MRI for mapping brain iron offer precise quantitative measurements of spatial distributions of magnetic susceptibility and have shown promise for imaging isotropic susceptibility, which is dominated by metals in tissue, including iron and calcium.^[^ ^397^, ^398^ ^]^ Moreover, positron emission tomography (PET) imaging with tracers such as ^18^F‐TRX, ^18^F‐FSPG, and ^68^Ga‐NOTATf enables real‐time, noninvasive monitoring of ferroptosis in vivo, by measuring the intracellular LIP, system Xc^−^ activity, and TF uptake, respectively.^[^ ^399^, ^400^, ^401^, ^402^ ^]^ The artemisinin‐based MRI probe (Art‐Gd) exploits redox‐active Fe^2+^ for ferroptosis detection, enabling the diagnosis of injuries 24–48 hours earlier than standard clinical assays.^[^ ^403^ ^] 64^Cu‐based PET tracers can map copper distribution in vivo, potentially identifying tumors susceptible to cuproptosis‐based therapies.^[^ ^404^, ^405^, ^406^ ^]^ Photoacoustic (PA) imaging, developed rapidly in the past decade, represents a noninvasive biomedical imaging method.^[^ ^407^, ^408^ ^]^ The etching of Fe‐Cu@PANI resulted in both photoacoustic imaging of tumors and efficient photothermal therapy.^[^ ^409^ ^]^ A self‐assembled nanosystem consisting of a semiconducting polymer and encapsulated ultrasmall CuS nanoparticles demonstrated not only an improved PA‐imaging ability but also significant tumor growth inhibition.^[^ ^410^ ^]^

Ultimately, the insights gained from studying metal‐dependent RCDs have profound implications for precision medicine. The future of this field lies in moving beyond a one‐size‐fits‐all approach to one where treatment is tailored to the individual. This involves comprehensively profiling a patient's disease at the molecular level to understand its specific metal dependencies and RCD vulnerabilities. Such profiling would ideally include: genomic analysis of RCD pathway genes to identify mutations that confer sensitivity or resistance; metallomic analysis of tissue metal content to identify specific dysregulation; and lipidomic or metabolomic profiling to identify metabolic states that create therapeutic vulnerabilities.

## 7 Conclusion and Discussion

In this review, we have navigated the landscape of transition metal‐dependent RCD by intentionally weaving together the conceptual, mechanistic, and technological threads that define the field. We demonstrated how the established knowledge of ferroptosis and cuproptosis provides a robust foundation for asking new questions, such as the potential existence of “cobaltosis”. We have further argued that answering these questions is inseparable from the advanced technologies and methodologies that enable their investigation, forming a feedback loop where conceptual challenges drive technological innovation and vice versa. The result of this integrated approach is a deeper understanding of the “metal death code” and a clearer path toward novel therapeutic interventions.

Ferroptosis and cuproptosis have established that cells possess distinct programs to die in response to specific metallic insults. These pathways, with their unique molecular mechanisms and biochemical signatures, have moved from novel concepts to promising therapeutic targets in diseases ranging from cancer to neurodegeneration. While cobalt is known to induce cell death through pleiotropic effects—including oxidative stress, mitochondrial damage, and pseudohypoxia via HIF‐1α stabilization—the critical question remains whether these events coalesce into a unique, regulated pathway. Defining cobaltosis requires stringent differentiation from established death modalities such as apoptosis, necroptosis, and, most importantly, its metallic counterparts. The challenge lies in identifying a specific molecular sensor and a dedicated signaling cascade for cobalt that is not merely a reflection of generalized metal toxicity or an overlap with ferroptotic or cuproptotic mechanism. The existence of these distinct yet interconnected pathways points toward a sophisticated “metal death code.” This conceptual framework suggests that cells integrate multiple signals—including the identity, concentration, and subcellular location of the metal ion, alongside the cell's own metabolic and redox state—to orchestrate a specific death response. Common nodes such as ROS generation, mitochondrial function, and glutathione metabolism serve as points of convergence and crosstalk, yet the initial trigger and core executioner mechanism confer pathway specificity. Deciphering this code is fundamental to understanding cellular decision‐making under metallic stress.

Future progress hinges on the integration of advanced technologies. Multi‐omics analyses, super‐resolution imaging, and novel biosensors will be instrumental in mapping these complex networks. Furthermore, nanotechnology offers unprecedented tools for targeted delivery of metal ions or pathway modulators, enabling precise investigation and therapeutic intervention.

From a clinical perspective, the translational potential is immense. Inducing ferroptosis or cuproptosis in therapy‐resistant cancers offers a powerful new strategy, while inhibiting these pathways holds promise for treating ischemia‐reperfusion injury and neurodegenerative diseases. The success of these approaches will depend on the development of specific biomarkers for patient stratification and pharmacodynamic monitoring. Ultimately, by continuing to unravel the intricacies of metal‐dependent cell death, we pave the way for precision medicine tailored to the unique metabolic and metallomic vulnerabilities of disease.

## Conflict of Interest

The authors declare no conflict of interest.

## References

[1] R. S. Hotchkiss , A. Strasser , J. E. Mcdunn , P. E. Swanson , N. Engl. J. Med 2009, 361, 1570. 19828534 10.1056/NEJMra0901217 PMC3760419

[2] I. Khan , A. Yousif , M. Chesnokov , L. Hong , I. Chefetz , Pharmacol. Ther. 2021, 220, 107717. 33164841 10.1016/j.pharmthera.2020.107717

[3] D. Bertheloot , E. Latz , B. S. Franklin , Cell Mol. Immunol. 2021, 18, 1106. 33785842 10.1038/s41423-020-00630-3 PMC8008022

[4] L. Galluzzi , I. Vitale , J. M. Abrams , E. S. Alnemri , E. H. Baehrecke , M. V. Blagosklonny , T. M. Dawson , V. L. Dawson , W. S. El‐Deiry , S. Fulda , E. Gottlieb , D. R. Green , M. O. Hengartner , O. Kepp , R. A. Knight , S. Kumar , S. A. Lipton , X. Lu , F. Madeo , W. Malorni , P. Mehlen , G. Nuñez , M. E. Peter , M. Piacentini , D. C. Rubinsztein , Y. Shi , H.‐U. Simon , P. Vandenabeele , E. White , J. Yuan , et al., Cell Death Differ. 2011, 19, 107. 21760595 10.1038/cdd.2011.96 PMC3252826

[5] G. Kroemer , L. Galluzzi , P. Vandenabeele , J. Abrams , E. S. Alnemri , E. H. Baehrecke , M. V. Blagosklonny , W. S. El‐Deiry , P. Golstein , D. R. Green , M. Hengartner , R. A. Knight , S. Kumar , S. A. Lipton , W. Malorni , G. Nuñez , M. E. Peter , J. Tschopp , J. Yuan , M. Piacentini , B. Zhivotovsky , G. Melino , Cell Death Differ. 2008, 16, 3. 18846107 10.1038/cdd.2008.150 PMC2744427

[6] L. Galluzzi , I. Vitale , S. A. Aaronson , J. M. Abrams , D. Adam , P. Agostinis , E. S. Alnemri , L. Altucci , I. Amelio , D. W. Andrews , M. Annicchiarico‐Petruzzelli , A. V. Antonov , E. Arama , E. H. Baehrecke , N. A. Barlev , N. G. Bazan , F. Bernassola , M. J. M. Bertrand , K. Bianchi , M. V. Blagosklonny , K. Blomgren , C. Borner , P. Boya , C. Brenner , M. Campanella , E. Candi , D. Carmona‐Gutierrez , F. Cecconi , F. K.‐M. Chan , N. S. Chandel , et al., Cell Death Differ. 2018, 25, 486. 29362479 10.1038/s41418-017-0012-4 PMC5864239

[7] G. Kroemer , W. S. El‐Deiry , P. Golstein , M. E. Peter , D. Vaux , P. Vandenabeele , B. Zhivotovsky , M. V. Blagosklonny , W. Malorni , R. A. Knight , M. Piacentini , S. Nagata , G. Melino , Cell Death Differ. 2005, 12, 1463. 16247491 10.1038/sj.cdd.4401724

[8] L. Galluzzi , J. M. Bravo‐San Pedro , I. Vitale , S. A. Aaronson , J. M. Abrams , D. Adam , E. S. Alnemri , L. Altucci , D. Andrews , M. Annicchiarico‐Petruzzelli , E. H. Baehrecke , N. G. Bazan , M. J. Bertrand , K. Bianchi , M. V. Blagosklonny , K. Blomgren , C. Borner , D. E. Bredesen , C. Brenner , M. Campanella , E. Candi , F. Cecconi , F. K. Chan , N. S. Chandel , E. H. Cheng , J. E. Chipuk , J. A. Cidlowski , A. Ciechanover , T. M. Dawson , V. L. Dawson , et al., Differ. 2014, 22, 58.

[9] W. MARET , Free Radical Biol. Med. 2019, 134, 311. 30625394 10.1016/j.freeradbiomed.2019.01.006

[10] L. M. Sayre , G. Perry , M. A. Smith , Curr. Opin. Chem. Biol. 1999, 3, 220. 10226049 10.1016/S1367-5931(99)80035-0

[11] C. J. Chang , Nat. Chem. Biol. 2015, 11, 744. 26379012 10.1038/nchembio.1913

[12] D. Horn , A. Barrientos , IUBMB Life 2008, 60, 421. 18459161 10.1002/iub.50 PMC2864105

[13] J. A. Tainer , E. D. Getzoff , J. S. Richardson , D. C. Richardson , Nature 1983, 306, 284. 6316150 10.1038/306284a0

[14] N. Sanvisens , M. C Bañó , M. Huang , S. Puig , Mol. Cell 2011, 44, 759. 22152479 10.1016/j.molcel.2011.09.021 PMC3240860

[15] T. Furukawa , Y. Naitoh , H. Kohno , R. Tokunaga , S. Taketani , Life Sci. 1992, 50, 2059. 1608289 10.1016/0024-3205(92)90572-7

[16] G. G. Altobelli , S. Van Noorden , A. Balato , V. Cimini , Front. Med. 2020, 7, 2020. 10.3389/fmed.2020.00183 PMC7235401 32478084

[17] X. Wang , P. An , Z. Gu , Y. Luo , J. Luo , Int. J. Mol. Sci. 2021, 22, 7525. 34299144 10.3390/ijms22147525 PMC8305404

[18] M. Valko , K. Jomova , C. J. Rhodes , K. Kuča , K. Musílek , Arch. Toxicol 2015, 90, 1. 26343967 10.1007/s00204-015-1579-5

[19] K. Szentmihályi , Orvosi Hetilap 2019, 160, 1407. 31492083 10.1556/650.2019.31499

[20] R. Sil , A. S. Chakraborti , Front. Chem. 2025, 13. 10.3389/fchem.2025.1543455 PMC11893434 40070406

[21] D. C. Crans , K. Kostenkova , Commun.Chem. 2020, 3, 104. 36703349 10.1038/s42004-020-00341-w PMC9814583

[22] C. Van Cleave , D. C. Crans , Inorganics 2019, 7, 111.

[23] U. Krämer , I. N. Talke , M. Hanikenne , FEBS Lett. 2007, 581, 2263. 17462635 10.1016/j.febslet.2007.04.010

[24] Z. Ma , F. E. Jacobsen , D. P. Giedroc , Chem. Rev. 2009, 109, 4644. 19788177 10.1021/cr900077w PMC2783614

[25] H. Hu , Q, Xu , Z. Mo , X. Hu , Q. He , Z. Zhang , Z. Xu , J. Nanobiotechnol. 2022, 20, 457. 10.1186/s12951-022-01661-w PMC9590139 36274142

[26] Y. Wang , L. Zhang , F. Zhou , Cell Mol. Immunol. 2022, 19, 867. 35459854 10.1038/s41423-022-00866-1 PMC9338229

[27] P. A. Cobine , D. C. Brady , Mol. Cell 2022, 82, 1786. 35594843 10.1016/j.molcel.2022.05.001

[28] J. Li , F. Cao , H.‐L. Yin , Z.‐J. Huang , Z.‐T. Lin , N. Mao , B. Sun , G. Wang , Cell Death Dis. 2020, 11, 88. 32015325 10.1038/s41419-020-2298-2 PMC6997353

[29] L. Chen , J. Min , F. Wang , Signal Transduction Targeted Ther. 2022, 7, 378. 10.1038/s41392-022-01229-y PMC9681860 36414625

[30] H.‐F. Yan , T. Zou , Q.‐Z. Tuo , S. Xu , H. Li , A. A. Belaidi , P. Lei , Signal Transduction Targeted Ther. 2021, 6, 49. 10.1038/s41392-020-00428-9 PMC7858612 33536413

[31] Y. Li , H. Huang , J. Gao , J. Lu , G. Kang , Y. Ge , W. Jiang , X. Cai , G. Zhang , L. Liu , Nat. Commun. 2025, 16, 3419. 40210858 10.1038/s41467-025-58737-y PMC11986041

[32] Z.‐Y. Xi , C.‐Y. Fan , Y.‐Y. Jiang , X.‐R. Xi , G.‐Y. Nie , S. Zhu , J.‐J. Zhang , L. Xu , Theranostics 2025, 15, 4734. 40225560 10.7150/thno.107025 PMC11984402

[33] W. Fu , J. Wang , T. Li , Y. Qiao , Z. Zhang , X. Zhang , M. He , Y. Su , Z. Zhao , C. Li , R. Xiao , Y. Han , S. Zhang , Z. Liu , J. Lin , G. Chen , Y. Li , Q. Zhong , Nat. Chem. Biol. 2025, 21, 1238. 39915626 10.1038/s41589-025-01841-3

[34] Y. Yang , H. Fan , Z. Guo , ChemPlusChem 2024, 89, 202300624. 10.1002/cplu.202300624 38315756

[35] S. Dolma , S. L. Lessnick , W. C. Hahn , B. R. Stockwell , Cancer Cell 2003, 3, 285. 12676586 10.1016/s1535-6108(03)00050-3

[36] N. Yagoda , M. Von Rechenberg , E. Zaganjor , A. J. Bauer , W. S. Yang , D. J. Fridman , A. J. Wolpaw , I. Smukste , J. M. Peltier , J. J Boniface , R. Smith , S. L. Lessnick , S. Sahasrabudhe , B. R. Stockwell , Nature 2007, 447, 865. 10.1038/nature05859 PMC3047570 17568748

[37] D. E. Root , S. P. Flaherty , B. P. Kelley , B. R. Stockwell , Chem. Biol. 2003, 10, 881. 14522058 10.1016/j.chembiol.2003.08.009

[38] W. S. Yang , B. R. Stockwell , Chem. Biol. 2008, 15, 234. 18355723 10.1016/j.chembiol.2008.02.010 PMC2683762

[39] A. J. Wolpaw , B. R. Stockwell , Methods Enzymol. 2014, 545, 265. 25065894 10.1016/B978-0-12-801430-1.00011-1

[40] A. J. Wolpaw , K. Shimada , R. Skouta , M. E. Welsch , U. D. Akavia , D. Pe'er , F. Shaik , J. C Bulinski , B. R. Stockwell , Proc. Natl. Acad. Sci. USA 2011, 108, E771. 21896738 10.1073/pnas.1106149108 PMC3182736

[41] S. J. Dixon , D. N. Patel , M. Welsch , R. Skouta , E. D. Lee , M. Hayano , A. G. Thomas , C. E. Gleason , N. P. Tatonetti , B. S. Slusher , B. R. Stockwell , ELife 2014, 3. 10.7554/eLife.02523 PMC4054777 24844246

[42] W. S. Yang , R. Sriramaratnam , M. E. Welsch , K. Shimada , R. Skouta , V. S. Viswanathan , J. H. Cheah , P. A. Clemons , A. F. Shamji , C. B. Clish , L. M. Brown , A. W. Girotti , V. W. Cornish , S. L. Schreiber , B. R. Stockwell , Cell 2014, 156, 317. 24439385 10.1016/j.cell.2013.12.010 PMC4076414

[43] W. S. Yang , K. J. Kim , M. M. Gaschler , M. Patel , M. S. Shchepinov , B. R. Stockwell , Proc. Natl. Acad. Sci. USA 2016, 113, E4966. 27506793 10.1073/pnas.1603244113 PMC5003261

[44] S. Doll , B. Proneth , Y. Y. Tyurina , E. Panzilius , S. Kobayashi , I. Ingold , M. Irmler , J. Beckers , M. Aichler , A. Walch , H. Prokisch , D. Trümbach , G. Mao , F. Qu , H. Bayir , J. Füllekrug , C. H. Scheel , W. Wurst , J. A. Schick , V. E. Kagan , J. P. F. Angeli , M. Conrad , Nat. Chem. Biol. 2016, 13, 91. 27842070 10.1038/nchembio.2239 PMC5610546

[45] S. Doll , F. P. Freitas , R. Shah , M. Aldrovandi , M. C. Da Silva , I. Ingold , A. Goya Grocin , T. N. Xavier Da Silva , E. Panzilius , C. H. Scheel , A. Mourão , K. Buday , M. Sato , J. Wanninger , T. Vignane , V. Mohana , M. Rehberg , A. Flatley , A. Schepers , A. Kurz , D. White , M. Sauer , M. Sattler , E. W. Tate , W. Schmitz , A. Schulze , V. O'Donnell , B. Proneth , G. M. Popowicz , D. A. Pratt , et al., Nature 2019, 575, 693. 31634899 10.1038/s41586-019-1707-0

[46] V. A. N. Kraft , C. T. Bezjian , S. Pfeiffer , L. Ringelstetter , C. Müller , F. Zandkarimi , J. Merl‐Pham , X. Bao , N. Anastasov , J. Kössl , S. Brandner , J. D. Daniels , P. Schmitt‐Kopplin , S. M. Hauck , B. R. Stockwell , K. Hadian , J. A. Schick , ACS Cent. Sci. 2019, 6, 41. 31989025 10.1021/acscentsci.9b01063 PMC6978838

[47] Y. Zou , H. Li , E. T. Graham , A. A. Deik , J. K. Eaton , W. Wang , G. Sandoval‐Gomez , C. B. Clish , J. G. Doench , S. L. Schreiber , Nat. Chem. Biol. 2020, 16, 302. 32080622 10.1038/s41589-020-0472-6 PMC7353921

[48] Y. Ai , B. Yan , X. Wang , Mol. Cell. Oncol. 2021, 8, 1881393. 33860083 10.1080/23723556.2021.1881393 PMC8018466

[49] S. J. Dixon , B. R. Stockwell , Nat. Chem. Biol. 2013, 10, 9. 10.1038/nchembio.1416 24346035

[50] C. C. Winterbourn , Toxicol. Lett. 1995, 82–83, 969. 10.1016/0378-4274(95)03532-x 8597169

[51] D. B. Kell , BMC Med. Genomics 2009, 2, 2. 19133145 10.1186/1755-8794-2-2 PMC2672098

[52] T. Ganz , E. Nemeth , Nat. Rev. Immunol. 2015, 15, 500. 26160612 10.1038/nri3863 PMC4801113

[53] M. W. Hentze , M. U. Muckenthaler , B. Galy , C. Camaschella , Cell 2010, 142, 24. 20603012 10.1016/j.cell.2010.06.028

[54] M. Knutson , M. Wessling‐Resnick , Crit. Rev. Biochem. Mol. Biol. 2003, 38, 61. 12641343 10.1080/713609210

[55] R. D. Klausner , T. A. Rouault , J. B. Harford , Cell 1993, 72, 19. 8380757 10.1016/0092-8674(93)90046-s

[56] B. Galy , M. Conrad , M. Muckenthaler , Nat. Rev. Mol. Cell Biol. 2023, 25, 133. 37783783 10.1038/s41580-023-00648-1

[57] A. Dautry‐Varsat , Biochimie 1986, 68, 375. 2874839 10.1016/s0300-9084(86)80004-9

[58] R. S. Ohgami , D. R. Campagna , A. Mcdonald , M. D. Fleming , Blood 2006, 108, 1388. 16609065 10.1182/blood-2006-02-003681 PMC1785011

[59] M. D. Fleming , C. C. Trenor , M. A. Su , D. Foernzler , D. R. Beier , W. F. Dietrich , N. C. Andrews , Nat. Genet. 1997, 16, 383. 9241278 10.1038/ng0897-383

[60] P. M. Harrison , P. Arosio , Biochim. Biophys. Acta (BBA) – Bioenergetics 1996, 1275, 161. 8695634 10.1016/0005-2728(96)00022-9

[61] O. Kakhlon , Z. I. Cabantchik , Free Radical Biol. Med. 2002, 33, 1037. 12374615 10.1016/s0891-5849(02)01006-7

[62] P. Arosio , R. Ingrassia , P. Cavadini , Biochim. Biophys. Acta (BBA) – General Subjects 2009, 1790, 589. 18929623 10.1016/j.bbagen.2008.09.004

[63] E. C. Theil , Annu. Rev. Biochem. 1987, 56, 289. 3304136 10.1146/annurev.bi.56.070187.001445

[64] G. Cairo , S. Recalcati , A. Pietrangelo , G. Minotti , Free Radical Biol. Med. 2002, 32, 1237. 12057761 10.1016/s0891-5849(02)00825-0

[65] K. Salnikow , Semin. Cancer Biol. 2021, 76, 189. 33901632 10.1016/j.semcancer.2021.04.001

[66] M. Gao , P. Monian , N. Quadri , R. Ramasamy , X. Jiang , Mol. Cell 2015, 59, 298. 26166707 10.1016/j.molcel.2015.06.011 PMC4506736

[67] W. Hou , Y. Xie , X. Song , X. Sun , M. T. Lotze , H. J. Zeh , R. Kang , D. Tang , Autophagy 2016, 12, 1425. 27245739 10.1080/15548627.2016.1187366 PMC4968231

[68] J. D. Mancias , X. Wang , S. P. Gygi , J. W Harper , A. C. Kimmelman , Nature 2014, 509, 105. 24695223 10.1038/nature13148 PMC4180099

[69] S. J. Dixon , K. M. Lemberg , M. R. Lamprecht , R. Skouta , E. M. Zaitsev , C. E. Gleason , D. N. Patel , A. J. Bauer , A. M. Cantley , W. S. Yang , B. Morrison , B. R. Stockwell , Cell 2012, 149, 1060. 22632970 10.1016/j.cell.2012.03.042 PMC3367386

[70] C. Liang , X. Zhang , M. Yang , X. Dong , Adv. Mater. 2019, 31, 1904197. 10.1002/adma.201904197 31595562

[71] E. Nemeth , M. S. Tuttle , J. Powelson , M. B. Vaughn , A. Donovan , D. M. Ward , T. Ganz , J. Kaplan , Science 2004, 306, 2090. 15514116 10.1126/science.1104742

[72] G. O. Latunde‐Dada , Biochim. Biophys. Acta (BBA) – General Subjects 2017, 1861, 1893. 28552631 10.1016/j.bbagen.2017.05.019

[73] T. A. Rouault , W.‐H. Tong , Nat. Rev. Mol. Cell Biol. 2005, 6, 345. 15803140 10.1038/nrm1620

[74] R. Lill , Nature 2009, 460, 831. 19675643 10.1038/nature08301

[75] H. Wang , P. An , E. Xie , Q. Wu , X. Fang , H. Gao , Z. Zhang , Y. Li , X. Wang , J. Zhang , G. Li , L. Yang , W. Liu , J. Min , F. Wang , Hepatology 2017, 66, 449. 28195347 10.1002/hep.29117 PMC5573904

[76] P. N. Paradkar , K. B. Zumbrennen , B. H. Paw , D. M. Ward , J. Kaplan , Mol. Cell. Biol. 2009, 29, 1007. 19075006 10.1128/MCB.01685-08 PMC2643804

[77] G. C. Shaw , J. J. Cope , L. Li , K. Corson , C. Hersey , G. E. Ackermann , B. Gwynn , A. J. Lambert , R. A. Wingert , D. Traver , N. S. Trede , B. A. Barut , Y. Zhou , E. Minet , A. Donovan , A. Brownlie , R. Balzan , M. J. Weiss , L. L. Peters , J. Kaplan , L. I. Zon , B. H. Paw , Nature 2006, 440, 96. 16511496 10.1038/nature04512

[78] Y. Zhao , M. Yang , X. Liang , J. Transl. Med. 2024, 22, 1057. 39587666 10.1186/s12967-024-05740-4 PMC11587765

[79] J. F. Turrens , J. Physiol. 2003, 552, 335. 14561818 10.1113/jphysiol.2003.049478 PMC2343396

[80] S. Ma , E. S. Henson , Y. Chen , S. B. Gibson , Cell Death Dis. 2016, 7, 2307. 10.1038/cddis.2016.208 PMC4973350 27441659

[81] M. W. Hentze , L. C. Kühn , Proc. Natl. Acad. Sci. USA 1996, 93, 8175.8710843

[82] C. P. Anderson , M. Shen , R. S. Eisenstein , E. A. Leibold , Biochim. Biophys. Acta (BBA) – Mol. Cell Res. 2012, 1823, 1468. 10.1016/j.bbamcr.2012.05.010 PMC3675657 22610083

[83] M. Kobayashi , M. Yamamoto , Antioxid. Redox Signaling 2005, 7, 385. 10.1089/ars.2005.7.385 15706085

[84] Q. Dang , Z. Sun , Y. Wang , L. Wang , Z. Liu , X. Han , Cell Death Dis. 2022, 13, 925. 36335094 10.1038/s41419-022-05384-6 PMC9637147

[85] X. Li , Y. Li , H. Tuerxun , Y. Zhao , X. Liu , Y. Zhao , Biomed. Pharmacother. 2024, 179, 117298. 39151313 10.1016/j.biopha.2024.117298

[86] X. Li , N. Ma , J. Xu , Y. Zhang , P. Yang , X. Su , Y. Xing , N. An , F. Yang , G. Zhang , L. Zhang , Y. Xing , Oxid. Med. Cell. Longev. 2021, 2021. 10.1155/2021/1587922 PMC8568519 34745412

[87] N. Sun , Y. Xing , J. Jiang , P. Wu , L. Qing , J. Tang , Heliyon 2023, 9, 20363. 10.1016/j.heliyon.2023.e20363 PMC10520329 37767486

[88] L. Zhou , S. Han , J. Guo , T. Qiu , J. Zhou , L. Shen , Cells 2022, 11, 3653. 36429080 10.3390/cells11223653 PMC9688314

[89] H.‐F. Yan , Q.‐Z. Tuo , Q.‐Z. Yin , P. Lei , Zool. Res. 2020, 41, 220. 32314558 10.24272/j.issn.2095-8137.2020.042 PMC7231469

[90] C. O. Reichert , F. A. De Freitas , J. Sampaio‐Silva , L. Rokita‐Rosa , P. D. L Barros , D. Levy , S. P. Bydlowski , Int. J. Mol. Sci. 2020, 21, 8765. 33233496 10.3390/ijms21228765 PMC7699575

[91] M. Alrouji , S. Anwar , K. Venkatesan , M. Shahwan , M. d. I Hassan , A. Islam , A. Shamsi , Ageing Res. Rev. 2024, 102, 102575. 39515619 10.1016/j.arr.2024.102575

[92] Y. Ji , K. Zheng , S. Li , S. Li , C. Ren , Y. Shen , L. Tian , H. Zhu , Z. Zhou , Y. Jiang , Front. Cell. Neurosci. 2022, 16, 2022. 10.3389/fncel.2022.1005182 PMC9647641 36385946

[93] G. Lei , L. Zhuang , B. Gan , Cancer Cell 2024, 42, 513. 38593779 10.1016/j.ccell.2024.03.011

[94] C. Zhang , X. Liu , S. Jin , Y. Chen , R. Guo , Mol. Cancer 2022, 21, 47. 35151318 10.1186/s12943-022-01530-y PMC8840702

[95] D. TANG , R. KANG , Cancer Drug Resistance 2024, 7, 41. 39534872 10.20517/cdr.2024.123 PMC11555182

[96] Y. Wang , G. Yu , X. Chen , Cancer Drug Resistance 2024, 7, 47. 39624080 10.20517/cdr.2024.127 PMC11609146

[97] Z. Zhao , J. Wu , H. Xu , C. Zhou , B. Han , H. Zhu , Z. Hu , Z. Ma , Z. Ming , Y. Yao , R. Zeng , G. Xu , Cell Death Dis. 2020, 11, 629. 32796819 10.1038/s41419-020-02871-6 PMC7429848

[98] X. Jin , J. Tang , X. Qiu , X. Nie , S. Ou , G. Wu , R. Zhang , J. Zhu , Cell Death Discovery 2024, 10, 45. 38267442 10.1038/s41420-024-01825-7 PMC10808233

[99] Q. Zhou , Y. Meng , D. Li , L. Yao , J. Le , Y. Liu , Y. Sun , F. Zeng , X. Chen , G. Deng , Signal Transduction Targeted Ther. 2024, 9, 55. 10.1038/s41392-024-01769-5 PMC10920854 38453898

[100] W. Ma , N. Hu , W. Xu , L. Zhao , C. Tian , K.‐I. Kamei , Bioorg. Chem. 2024, 146, 107331. 38579614 10.1016/j.bioorg.2024.107331

[101] S. Sun , J. Shen , J. Jiang , F. Wang , J. Min , Signal Transduction Targeted Ther. 2023, 8, 372. 10.1038/s41392-023-01606-1 PMC10514338 37735472

[102] J. Diao , Y. Jia , E. Dai , J. Liu , R. Kang , D. Tang , L. Han , Y. Zhong , L. Meng , Mol. Cancer 2024, 23, 89. 38702722 10.1186/s12943-024-01999-9 PMC11067110

[103] O. Zilka , R. Shah , B, Li , J. P. Friedmann Angeli , M. Griesser , M. Conrad , D. A. Pratt , ACS Cent. Sci. 2017, 3, 232. 28386601 10.1021/acscentsci.7b00028 PMC5364454

[104] M. Zhang , X. Chen , Y. Zhang , Antioxidants 2024, 13, 1571.39765898

[105] C. Scarpellini , G. Klejborowska , C. Lanthier , B. Hassannia , T. Vanden Berghe , K. Augustyns , Trends Pharmacol. Sci. 2023, 44, 902. 37770317 10.1016/j.tips.2023.08.012

[106] P. Tsvetkov , A. Detappe , K. Cai , H. R. Keys , Z. Brune , W. Ying , P. Thiru , M. Reidy , G. Kugener , J. Rossen , M. Kocak , N. Kory , A. Tsherniak , S. Santagata , L. Whitesell , I. M. Ghobrial , J. L. Markley , S. Lindquist , T. R. Golub , Nat. Chem. Biol. 2019, 15, 681. 31133756 10.1038/s41589-019-0291-9 PMC8183600

[107] P. Tsvetkov , S. Coy , B. Petrova , M. Dreishpoon , A. Verma , M. Abdusamad , J. Rossen , L. Joesch‐Cohen , R. Humeidi , R. D. Spangler , J. K. Eaton , E. Frenkel , M. Kocak , S. M. Corsello , S. Lutsenko , N. Kanarek , S. Santagata , T. R. Golub , Science 2022, 375, 1254. 35298263 10.1126/science.abf0529 PMC9273333

[108] J. R. Gale , K. Hartnett‐Scott , M. M. Ross , P. A. Rosenberg , E. Aizenman , J. Neurochem. 2023, 167, 277. 37702109 10.1111/jnc.15961 PMC10591933

[109] M. Zulkifli , A. N. Spelbring , Y. Zhang , S. Soma , S. Chen , L. Li , T. Le , V. Shanbhag , M. J. Petris , T.‐Y. Chen , M. Ralle , D. P. Barondeau , V. M. Gohil , Proc. Natl. Acad. Sci. USA 2023, 120, 2216722120. 10.1073/pnas.2216722120 PMC10013847 36848556

[110] M. Pan , Q. Zheng , Y. Yu , H. Ai , Y. Xie , X. Zeng , C. Wang , L. Liu , M. Zhao , Nat. Commun. 2021, 12, 121. 33402676 10.1038/s41467-020-20359-x PMC7785736

[111] Z. Skrott , D. Majera , J. Gursky , T. Buchtova , M. Hajduch , M. Mistrik , J. Bartek , Oncogene 2019, 38, 6711. 31391554 10.1038/s41388-019-0915-2

[112] B.‐E. Kim , T. Nevitt , D. J. Thiele , Nat. Chem. Biol. 2008, 4, 176. 18277979 10.1038/nchembio.72

[113] J. F. Eisses , Y. Chi , J. H. Kaplan , J. Biol. Chem. 2005, 280, 9635. 15634665 10.1074/jbc.M500116200

[114] I. Hamza , J. Prohaska , J. D. Gitlin , Proc. Natl. Acad. Sci. USA 2003, 100, 1215. 12538877 10.1073/pnas.0336230100 PMC298753

[115] I. Hamza , M. Schaefer , L. W. J. Klomp , J. D. Gitlin , Proc. Natl. Acad. Sci. USA 1999, 96, 13363. 10557326 10.1073/pnas.96.23.13363 PMC23953

[116] W. I. M. Vonk , P. De Bie , C. G. K. Wichers , P. V. E. Van Den Berghe , R. Van Der Plaats , R. Berger , C. Wijmenga , L. W. J. Klomp , B. Van De Sluis , Cell. Mol. Life Sci. 2011, 69, 149. 21667063 10.1007/s00018-011-0743-1 PMC3249196

[117] S. Narindrasorasak , P. Kulkarni , P. Deschamps , Y.‐M. She , B. Sarkar , Biochemistry 2007, 46, 3116. 17309234 10.1021/bi0620656

[118] P. de Bie , B. van de Sluis , E. Burstein , P. V. E. van de Berghe , P. Muller , R. Berger , J. D. Gitlin , C. Wijmenga , L. W. J. Klomp , Gastroenterology 2007, 133, 1316. 17919502 10.1053/j.gastro.2007.07.020 PMC2857755

[119] V. C. Culotta , L. W. J. Klomp , J. Strain , R. L. B. Casareno , B. Krems , J. D. Gitlin , J. Biol. Chem. 1997, 272, 23469. 9295278 10.1074/jbc.272.38.23469

[120] S. Puig , D. J. Thiele , Curr. Opin. Chem. Biol. 2002, 6, 171. 12039001 10.1016/s1367-5931(02)00298-3

[121] P. A. Cobine , F. Pierrel , D. R. Winge , Biochim. Biophys. Acta (BBA) – Mol. Cell Res. 2006, 1763, 759. 10.1016/j.bbamcr.2006.03.002 16631971

[122] X. Zhu , A. Boulet , K. M. Buckley , C. B. Phillips , M. G. Gammon , L. E. Oldfather , S. A. Moore , S. C. Leary , P. A. Cobine , eLife 2021, 10, 64690. 10.7554/eLife.64690 PMC7924939 33591272

[123] J. Bertinato , M. R. L'Abbé , J. Nutr. Biochem. 2004, 15, 316. 15157936 10.1016/j.jnutbio.2004.02.004

[124] N. E. Hellman , J. D. C Gitlin , Annu. Rev. Nutr. 2002, 22, 439. 12055353 10.1146/annurev.nutr.22.012502.114457

[125] J. R. Prohaska , Am. J. Clin. Nutr. 2008, 88, 826SS. 10.1093/ajcn/88.3.826S PMC2799992 18779302

[126] S. Zhang , Q. Huang , T. Ji , Q. Li , C. Hu , Biomarker Res. 2024, 12, 130. 10.1186/s40364-024-00677-8 PMC11529036 39482784

[127] K. M. Abdullah , J. B. Kaushal , S. Takkar , G. Sharma , Z. W. Alsafwani , R. Pothuraju , S. K. Batra , J. A. Siddiqui , Heliyon 2024, 10, 27496. 10.1016/j.heliyon.2024.e27496 PMC10938126 38486750

[128] A. Ressnerova , M. Raudenska , M. Holubova , M. Svobodova , H. Polanska , P. Babula , M. Masarik , J. Gumulec , Curr. Med. Chem. 2016, 23, 1304. 27048341 10.2174/0929867323666160405111543

[129] P. Zheng , C. Zhou , L. Lu , B. Liu , Y. Ding , J. Exp. Clin. Cancer Res. 2022, 41, 271. 36089608 10.1186/s13046-022-02485-0 PMC9465867

[130] X. Kang , S. Jadhav , M. Annaji , C.‐H. Huang , R. Amin , J. Shen , C. R. Ashby , A. K. Tiwari , R. J Babu , P. Chen , Pharmaceutics 2023, 15, 1567. 37376016 10.3390/pharmaceutics15061567 PMC10302862

[131] V. Oliveri , Front. Mol. Biosci. 2022, 9, 841814. 35309510 10.3389/fmolb.2022.841814 PMC8931543

[132] D. Noh , H. Lee , S. Lee , I.‐C. Sun , H. Y. Yoon , Biomater. Res. 2024, 28, 0094. 39430913 10.34133/bmr.0094 PMC11486892

[133] H. Cheng , L. Zhao , J. Cai , Mater. Today Bio 2025, 32, 101894. 10.1016/j.mtbio.2025.101894 PMC12152900 40502371

[134] Q. Zhao , T. Qi , Front. Oncol. 2023, 13. 10.3389/fonc.2023.1117164 PMC10011146 36925927

[135] P. C. Bull , G. R. Thomas , J. M. Rommens , J. R. Forbes , D. W. Cox , Nat. Genet. 1993, 5, 327. 8298639 10.1038/ng1293-327

[136] R. E. Tanzi , K. Petrukhin , I. Chernov , J. L. Pellequer , W. Wasco , B. Ross , D. M. Romano , E. Parano , L. Pavone , L. M. Brzustowicz , M. Devoto , J. Peppercorn , A. I. Bush , I. Sternlieb , M. Pirastu , J. F. Gusella , O. Evgrafov , G. K. Penchaszadeh , B. Honig , I. S. Edelman , M. B. Soares , I. H. Scheinberg , T. C. Gilliam , Nat. Genet. 1993, 5, 344. 8298641 10.1038/ng1293-344

[137] Z. Tao , S. Kang , J. Liu , R. Wang , J. Zhou , W. Yang , M. Wang , Medicine 2024, 103, 40598. 10.1097/MD.0000000000040598 PMC11596713 39809184

[138] G. Gromadzka , B. Tarnacka , A. Flaga , A. Adamczyk , Int. J. Mol. Sci. 2020, 21, 9259. 33291628 10.3390/ijms21239259 PMC7730516

[139] R. Squitti , M. C. A. Rongioletti , G. Liguri , Vitamins and Minerals in Neurological Disorders, Academic Press, Cambridge, MA 2023, pp. 65‐85.

[140] C.‐J. Lin , H.‐C. Huang , Z.‐F. Jiang , Brain Res. Bull. 2010, 82, 235. 20598459 10.1016/j.brainresbull.2010.06.003

[141] G. Walke , R. Kumar , P. Wittung‐Stafshede , Protein Sci. 2024, 33, 4956. 10.1002/pro.4956 PMC10955613 38511511

[142] T. Decker , M.‐L. Lohmann‐Matthes , J. Immunol. Methods 1988, 115, 61. 3192948 10.1016/0022-1759(88)90310-9

[143] F. Denizot , R. Lang , J. Immunol. Methods 1986, 89, 271. 3486233 10.1016/0022-1759(86)90368-6

[144] H. Ohkawa , N. Ohishi , K. Yagi , Anal. Biochem. 1979, 95, 351. 36810 10.1016/0003-2697(79)90738-3

[145] G. P. C. Drummen , L. C. M. Van Liebergen , J. A. F. Op Den Kamp , J. A. Post , Free Radical Biol. Med. 2002, 33, 473. 12160930 10.1016/s0891-5849(02)00848-1

[146] T. Hirayama , K. Okuda , H. Nagasawa , Chem. Sci. 2013, 4, 1250.

[147] D. Stephenson , T. Nemkov , S. M. Qadri , W. P. Sheffield , A. D'Alessandro , Front. Physiol. 2022, 13, 828087. 35197866 10.3389/fphys.2022.828087 PMC8859330

[148] C. L. Williams , H. M. Neu , S. L. J. Michel , D. S. Merrell , Acinetobacter baumannii Methods and Protocols, Springer, New York 2019, pp. 195–205.

[149] A. J. Miller , S. A. Center , J. F. Randolph , C. H. Friesen , A. D. Miller , K. W. Warner , J. Am. Vet. Med. Assoc. 2021, 258, 395. 33539202 10.2460/javma.258.4.395

[150] S. C. Dodani , S. C. Leary , P. A. Cobine , D. R. Winge , C. J. Chang , J. Am. Chem. Soc. 2011, 133, 8606. 21563821 10.1021/ja2004158 PMC3106114

[151] X. Qian , W. Zhu , H. Yu , Y. Xu , W. Liu , H.‐Y. Wang , Y/ Liu , Dyes Pigm. 2021, 194, 109561.

[152] H. Li , R. Zhang , C. Li , B. Huang , T. Yu , X. Huang , X. Zhang , F. Li , H. Zhou , Y. Tian , Org. Biomol. Chem. 2017, 15, 598. 27929196 10.1039/c6ob02384c

[153] S. T. Smiley , M. Reers , C. Mottola‐Hartshorn , M. Lin , A. Chen , T. W. Smith , G. D. Steele , L. B. Chen , Proc. Natl. Acad. Sci. USA 1991, 88, 3671. 2023917 10.1073/pnas.88.9.3671 PMC51514

[154] J. K. Caines , D. A. Barnes , M. D. Berry , Cancer Cell Biology, Springer, New York 2022.

[155] W. Voos , A. Wilkening , R. Ostermann , M. Bruderek , W. Jaworek , L. Ruland , Methods Enzymol. 2024, 707, 475. 39488387 10.1016/bs.mie.2024.07.048

[156] Y. Zhang , R. V. Swanda , L. Nie , X. Liu , C. Wang , H. Lee , G. Lei , C. Mao , P. Koppula , W. Cheng , J. Zhang , Z. Xiao , L. Zhuang , B. Fang , J. Chen , S.‐B. Qian , B. Gan , Nat. Commun. 2021, 12, 1589. 33707434 10.1038/s41467-021-21841-w PMC7952727

[157] B. R. Stockwell , J. P. Friedmann Angeli , H. Bayir , A. I. Bush , M. Conrad , S. J. Dixon , S. Fulda , S. Gascón , S. K. Hatzios , V. E. Kagan , K. Noel , X. Jiang , A. Linkermann , M. E. Murphy , M. Overholtzer , A. Oyagi , G. C. Pagnussat , J. Park , Q. Ran , C. S. Rosenfeld , K. Salnikow , D. Tang , F. M. Torti , S. V. Torti , S. Toyokuni , K. A. Woerpel , D. D. Zhang , Cell 2017, 171, 273. 28985560 10.1016/j.cell.2017.09.021 PMC5685180

[158] J. P. Friedmann Angeli , M. Schneider , B. Proneth , Y. Y. Tyurina , V. A. Tyurin , V. J. Hammond , N. Herbach , M. Aichler , A. Walch , E. Eggenhofer , D. Basavarajappa , O. Rådmark , S. Kobayashi , T. Seibt , H. Beck , F. Neff , I. Esposito , R. Wanke , H. Förster , O. Yefremova , M. Heinrichmeyer , G. W. Bornkamm , E. K. Geissler , S. B. Thomas , B. R. Stockwell , V. B. O'Donnell , V. E. Kagan , J. A. Schick , M. Conrad , Nat. Cell Biol. 2014, 16, 1180. 25402683 10.1038/ncb3064 PMC4894846

[159] L. Zhang , R. Deng , L. Liu, H. Du , D. Tang , Front. Mol. Biosci. 2024, 11, 2024. 10.3389/fmolb.2024.1477971 PMC11628392 39659361

[160] C.‐H. Lin , Y. Chin , M. Zhou , R. W. Sobol , M.‐C. Hung , M. Tan , Trends Biochem. Sci. 2024, 49, 729. 38714376 10.1016/j.tibs.2024.04.002

[161] C. P. Lebel , H. Ischiropoulos , S. C. Bondy , Chem. Res. Toxicol. 1992, 5, 227. 1322737 10.1021/tx00026a012

[162] P. Mukhopadhyay , M. Rajesh , K. Yoshihiro , G. Haskó , P. Pacher , Biochem. Biophys. Res. Commun. 2007, 358, 203. 17475217 10.1016/j.bbrc.2007.04.106 PMC2228267

[163] A. Seiler , M. Schneider , H. Förster , S. Roth , E. K. Wirth , C. Culmsee , N. Plesnila , E. Kremmer , O. Rådmark , W. Wurst , G. W. Bornkamm , U. Schweizer , M. Conrad , Cell Metab. 2008, 8, 237. 18762024 10.1016/j.cmet.2008.07.005

[164] D. Huster , M. J. Finegold , C. T. Morgan , J. L. Burkhead , R. Nixon , S. M. Vanderwerf , C. T. Gilliam , S. Lutsenko , Am. J. Pathol. 2006, 168, 423. 16436657 10.2353/ajpath.2006.050312 PMC1606493

[165] B. Varynskyi , J. A. Schick , Biomedicines 2024, 12, 541. 38540154 10.3390/biomedicines12030541 PMC10968238

[166] Y. Y. Tyurina , A. A. Kapralov , V. A. Tyurin , G. Shurin , A. A. Amoscato , D. Rajasundaram , H. Tian , Y. L. Bunimovich , Y. Nefedova , W. G. Herrick , R. E. Parchment , J. H. Doroshow , H. Bayir , A. K. Srivastava , V. E. Kagan , Redox Biol. 2023, 61, 102650. 36870109 10.1016/j.redox.2023.102650 PMC9996109

[167] R. Zhang , G. Kroemer , D. Tang , Life Metabolism 2024, 3, loae008. 38523816 10.1093/lifemeta/loae008 PMC10960586

[168] A. T. M. Van Kessel , R. Karimi , G. Cosa , Chem. Sci. 2022, 13, 9727. 36091918 10.1039/d2sc00525e PMC9400630

[169] D. Chen , I. Y. Eyupoglu , N. Savaskan , Cell Viability Assays, Springer, New York 2017, pp. 71–77.

[170] J. Jin , K. Schorpp , D. Samaga , K. Unger , K. Hadian , B. R. Stockwell , ACS Chem. Biol. 2022, 17, 654. 35230809 10.1021/acschembio.1c00953 PMC8938922

[171] K. Schorpp , A. Bessadok , A. Biibosunov , I. Rothenaigner , S. Strasser , T. Peng , K. Hadian , Cell Death Discov. 2023, 9, 277. 37524741 10.1038/s41420-023-01559-y PMC10390533

[172] X. Hui , M. d. Sharifuzzaman , S. Sharma , X. Xuan , S. Zhang , S. G. Ko , S. H. Yoon , J. Y. Park , ACS Appl. Mater. Interfaces 2020, 12, 48928. 33074662 10.1021/acsami.0c12239

[173] B. Lorber , F. Fischer , M. Bailly , H. Roy , D. Kern , Biochem. Mol. Biol. Educ. 2012, 40, 372. 23166025 10.1002/bmb.20644

[174] W. Wang , C. J. Roberts , Int. J. Pharm. 2018, 550, 251. 30145245 10.1016/j.ijpharm.2018.08.043

[175] F. O'Leary , S. Samman , Nutrients 2010, 2, 299. 22254022 10.3390/nu2030299 PMC3257642

[176] R. Banerjee , S. W. Ragsdale , Annu. Rev. Biochem. 2003, 72, 209. 14527323 10.1146/annurev.biochem.72.121801.161828

[177] G. Genchi , G. Lauria , A. Catalano , A. Carocci , M. S. Sinicropi , Biology 2023, 12, 1335. 37887045 10.3390/biology12101335 PMC10604320

[178] S. Leonard , P. M. Gannett , Y. Rojanasakul , D. Schwegler‐Berry , V. Castranova , V. Vallyathan , X. Shi , J. Inorg. Biochem. 1998, 70, 239. 9720310 10.1016/s0162-0134(98)10022-3

[179] A. W. Foster , D. Osman , N. J. ROBINSON , J. Biol. Chem. 2014, 289, 28095. 25160626 10.1074/jbc.R114.588145 PMC4192464

[180] A. C. Illing , A. Shawki , C. L. Cunningham , B. Mackenzie , J. Biol. Chem. 2012, 287, 30485. 22736759 10.1074/jbc.M112.364208 PMC3436370

[181] N. Zaksas , Y. Gluhcheva , S. Sedykh , M. Madzharova , N. Atanassova , G. Nevinsky , J. Trace Elements Med. Biol. 2013, 27, 27. 10.1016/j.jtemb.2012.07.005 22944586

[182] B. A Mosier , L. Maynard , N. G. Sotereanos , J. J Sewecke , Am. J. Orthop. 2016, 45, E132. 26991580

[183] S. S. Tower , J. Bone Joint Surg. 2010, 92, 2847. 21037026 10.2106/JBJS.J.00125

[184] S. S. Tower , BMJ 2012, 344, e430. 22252702 10.1136/bmj.e430

[185] J. R. W. Crutsen , M. C. Koper , J. Jelsma , M. Heymans , I. C. Heyligers , B. Grimm , N. M. C. Mathijssen , M. G. M. Schotanus , EFORT Open Rev. 2022, 7, 188. 35298414 10.1530/EOR-21-0098 PMC8965198

[186] M. Umar , N. Jahangir , M. Faisal Khan , Z. Saeed , F. Sultan , A. Sultan , Arthroplasty Today 2019, 5, 371. 31516984 10.1016/j.artd.2019.04.010 PMC6728440

[187] K. Anna , D. Wojciech , T. Maciej , et al., Int. J. Occup. Med. Environ. Health 2005, 18, 151.16201206

[188] A. Al‐Abcha , L. Wang , M. J. Reilly , K. D. Rosenman , J. Asthma 2020, 58, 1032. 32308078 10.1080/02770903.2020.1759090

[189] Y. Yuan , G. Hilliard , T. Ferguson , D. E. Millhorn , J. Biol. Chem. 2003, 278, 15911. 12606543 10.1074/jbc.M300463200

[190] H. F. Bunn , Cold Spring Harbor Perspect. Med. 2013, 3, a011619. 10.1101/cshperspect.a011619 PMC3579209 23457296

[191] B. Ebert , W. Jelkmann , Drug Test. Anal. 2013, 6, 185. 24039233 10.1002/dta.1528

[192] L. O. Simonsen , H. Harbak , P. Bennekou , Sci. Total Environ. 2012, 432, 210. 22732165 10.1016/j.scitotenv.2012.06.009

[193] L. O. Simonsen , H. Harbak , P. Bennekou , Blood Cells, Mol. Dis. 2011, 47, 214. 21962619 10.1016/j.bcmd.2011.09.002

[194] U. Kasten , A. Hartwig , D. Beyersmann , Arch. Toxicol. 1992, 66, 592. 1281401 10.1007/BF01973391

[195] R. Ortega , C. Bresson , A. Fraysse , C. Sandre , G. Devès , C. Gombert , M. Tabarant , P. Bleuet , H. Seznec , A. Simionovici , P. Moretto , C. Moulin , Toxicol. Lett. 2009, 188, 26. 19433266 10.1016/j.toxlet.2009.02.024

[196] F. Barras , M. Fontecave , Metallomics 2011, 3, 1130. 21952637 10.1039/c1mt00099c

[197] J. Morrissey , I. R. Baxter , J. Lee , L. Li , B. Lahner , N. Grotz , J. Kaplan , D. E. Salt , M. L. Guerinot , Plant Cell 2009, 21, 3326. 19861554 10.1105/tpc.109.069401 PMC2782287

[198] D. A. Rodionov , P. Hebbeln , M. S. Gelfand , T. Eitinger , J. Bacteriol. 2006, 188, 317. 16352848 10.1128/JB.188.1.317-327.2006 PMC1317602

[199] T. Eitinger , Encycl. Inorg. Bioinorg. Chem. 2013, 1.

[200] P. Mihelj , I. Abreu , T. Moreyra , M. González‐Guerrero , D. Raimunda , Appl. Environ. Microbiol. 2023, 89, 3. 10.1128/aem.01901-22 PMC10057888 36853042

[201] H. Komeda , M. Kobayashi , S. Shimizu , Proc. Natl. Acad. Sci. USA 1997, 94, 36. 8990157 10.1073/pnas.94.1.36 PMC19232

[202] D. Koch , D. H. Nies , G. Grass , BioMetals 2006, 20, 759. 17120142 10.1007/s10534-006-9039-6

[203] R. Hausinger , D. Zamble , Molecular Microbiology of Heavy Metals, Springer, New York 2007, pp 287–320.

[204] D. Galea , M. Herzberg , D. H. Nies , J. Bacteriol. 2024, 206, e00226. 39041725 10.1128/jb.00226-24 PMC11340326

[205] Y. Zhao , M. Kong , J. Yang , X. Zhao , Y. Shi , Y. Zhai , J. Qiu , C. Zheng , Int. J. Mol. Sci. 2022, 24, 414. 36613858 10.3390/ijms24010414 PMC9820535

[206] N. J. Hawco , M. A. Saito , Limnol. Oceanogr. 2018, 63, 2229.

[207] D. S. Conklin , J. A. McMaster , M. R. Culbertson , et al., Mol. Cell. Biol. 1992, 12, 3678. 1508175 10.1128/mcb.12.9.3678-3688.1992 PMC360222

[208] P. Chang , H. Yin , T. Imanaka , Y. Igarashi , N. Li , F. Luo , Biochem. Biophys. Res. Commun. 2020, 523, 880. 31955886 10.1016/j.bbrc.2019.12.121

[209] S. Terpiłowska , K. Rafińska , A. Gołębiowski , T. Kowalkowski , B. Buszewski , Ecol. Chem. Eng. S 2023, 30, 471.

[210] A. Kanaji , V. Orhue , M. S. Caicedo , A. S. Virdi , D. R. Sumner , N. J. Hallab , T. Yoshiaki , K. Sena , J. Orthop. Surg. Res. 2014, 9, 91. 25288055 10.1186/s13018-014-0091-6 PMC4194407

[211] C. Angelé‐Martínez , J. Murray , P. A. Stewart , J. Haines , A. A. E. Gaertner , J. L. Brumaghim , J. Inorg. Biochem. 2023, 238, 112024. 36272187 10.1016/j.jinorgbio.2022.112024

[212] M. Li , K. Li , Y. Ren , J. Biol. Res. 2021, 28, 21. 10.1186/s40709-021-00147-4 PMC8436528 34517917

[213] I. Fenoglio , I. Corazzari , C. Francia , S. Bodoardo , B. Fubini , Free Radical Res. 2008, 42, 437. 18712631 10.1080/10715760802350904

[214] Y. Yasukochi , M. Nakamura , S. Minakami , Biochem. J. 1974, 144, 455. 4468818 10.1042/bj1440455 PMC1168523

[215] N. Serrano‐García , R. Pinete‐Sánchez , O. N. Medina‐Campos , M. A. Ramos‐Santander , J. Pedraza‐Chaverri , M. Orozco‐Ibarra , Cell. Mol. Biol. 2024, 70, 53. 38678627 10.14715/cmb/2024.70.4.9

[216] W. Zou , J. Zeng , M. Zhuo , W. Xu , L. Sun , J. Wang , X. Liu , J. Neurosci. Res. 2002, 67, 837. 11891799 10.1002/jnr.10168

[217] O. L. Huk , I. Catelas , F. Mwale , J. Antoniou , D. J. Zukor , A. Petit , J. Arthroplasty 2004, 19, 84. 15578559 10.1016/j.arth.2004.09.011

[218] Z. Salloum , E. A. Lehoux , M.‐E. Harper , I. Catelas , J. Orthop. Res. 2018, 36, 3178. 30144138 10.1002/jor.24130

[219] B. Obied , S. Richard , A. Zahavi , D. Fixler , O. Girshevitz , N. Goldenberg‐Cohen , Cells 2024, 13, 1765. 39513872 10.3390/cells13211765 PMC11545114

[220] C. Ranquet , S. Ollagnier‐De‐Choudens , L. Loiseau , F. Barras , M. Fontecave , J. Biol. Chem. 2007, 282, 30442. 17642475 10.1074/jbc.M702519200

[221] A. Ayswarya , G. Kurian , Indian J. Pharm. Sci. 2016, 78, 151. 27168694 10.4103/0250-474x.180258 PMC4852565

[222] V. Battaglia , A. Compagnone , A. Bandino , M. Bragadin , C. A. Rossi , F. Zanetti , S. Colombatto , M. A. Grillo , A. Toninello , Int. J. Biochem. Cell Biol. 2009, 41, 586. 18708157 10.1016/j.biocel.2008.07.012

[223] E. Shumilina , O. Dobrovolska , R. Del Conte , H. W. Holen , A. Dikiy , JBIC J. Biol. Inorganic Chem. 2013, 19, 85. 10.1007/s00775-013-1064-7 PMC3889830 24271273

[224] M. Akbar , J. M. Brewer , M. H. Grant , J. Immunotoxicol. 2011, 8, 140. 21446789 10.3109/1547691X.2011.553845

[225] L. O. Simonsen , A. M. Brown , H. Harbak , B. I. Kristensen , P. Bennekou , Blood Cells, Mol., Dis. 2011, 46, 266. 21420882 10.1016/j.bcmd.2011.02.009

[226] S. Eshaghi , D. Niegowski , A. Kohl , D. M. Molina , S. A. Lesley , Pär Nordlund , Science 2006, 313, 354. 16857941 10.1126/science.1127121

[227] C. Abbehausen , Metallomics 2019, 11, 15. 30303505 10.1039/c8mt00262b

[228] A. Y. Louie , T. J. Meade , Proc. Natl. Acad. Sci. USA 1998, 95, 6663. 9618469 10.1073/pnas.95.12.6663 PMC22591

[229] C. R. Brue , M. W. Dukes , M. Masotti , R. Holmgren , T. J. Meade , ChemMedChem 2022, 17, 202200025. 10.1002/cmdc.202200025 PMC10826845 35302712

[230] Y.‐G. Gao , M. Sriram , A. H.‐J. Wang , Nucleic Acids Res. 1993, 21, 4093. 8371984 10.1093/nar/21.17.4093 PMC310011

[231] R. Wan , Y. Mo , L. Feng , S. Chien , D. J. Tollerud , Q. Zhang , Chem. Res. Toxicol. 2012, 25, 1402. 22559321 10.1021/tx200513t PMC3398242

[232] N. Rahmanian , M. Shokrzadeh , M. Eskandani , DNA Repair 2021, 108, 103243. 34710661 10.1016/j.dnarep.2021.103243

[233] D. R. Pilch , O. A. Sedelnikova , C. Redon , A. Celeste , A. Nussenzweig , W. M. Bonner , Biochem. Cell Biol. 2003, 81, 123. 12897845 10.1139/o03-042

[234] V. Kumar , R. K. Mishra , G. Kaur , D. Dutta , Metallomics 2017, 9, 1596. 29058747 10.1039/c7mt00231a

[235] P. Wang , A. B. Guliaev , B. Hang , Toxicol. Lett. 2006, 166, 237. 16938414 10.1016/j.toxlet.2006.06.647

[236] A. Hartwig , T. Schwerdtle , Toxicol. Lett. 2002, 127, 47. 12052640 10.1016/s0378-4274(01)00482-9

[237] L. Díez‐Tercero , L. M. Delgado , E. Bosch‐Rué , R. A. Perez , Sci. Rep. 2021, 11, 11707. 34083604 10.1038/s41598-021-91070-0 PMC8175577

[238] W. Zou , M. Yan , W. Xu , H. Huo , L. Sun , Z. Zheng , X. Liu , J. Neurosci. Res. 2001, 64, 646. 11398189 10.1002/jnr.1118

[239] A. Vengellur , Toxicol. Sci. 2004, 82, 638. 15375294 10.1093/toxsci/kfh278

[240] B.‐C. Cheng , J.‐T. Chen , S.‐T. Yang , C.‐C. Chio , S.‐H. Liu , R.‐M. Chen , Int. J. Oncol. 2017, 50, 964. 28197638 10.3892/ijo.2017.3861

[241] R. Chen , T. Jiang , Y. She , J. Xu , C. Li , S. Zhou , H. Shen , H. Shi , S. Liu , Biomed Res. Int. 2017, 2017, 7097580. 28706950 10.1155/2017/7097580 PMC5494548

[242] L. Sun , N. Liu , S.‐S. Liu , W.‐Y. Xia , M.‐Y. Liu , L.‐F. Li , J.‐X. Gao , Am. J. Cancer Res. 2015, 5, 2626. 26609472 PMC4633894

[243] S. Owada , H. Endo , Y. Shida , et al., Oncol. Rep. 2018, 39, 1805. 29484444 10.3892/or.2018.6279

[244] Z. Luo , Q. Gao , H. Zhang , Y. Zhang , S. Zhou , J. Zhang , W. Xu , J. Xu , Ecotoxicol. Environ. Saf. 2022, 232, 113219. 35104775 10.1016/j.ecoenv.2022.113219

[245] Y. Liu , W. Zhu , D. Ni , Z. Zhou , J.‐H. Gu , W. Zhang , H. Sun , F. Liu , J. Nanobiotechnol. 2020, 18, 141. 10.1186/s12951-020-00700-8 PMC7532644 33008409

[246] A. Triantafyllou , P. Liakos , A. Tsakalof , E. Georgatsou , G. Simos , S. Bonanou , Free Radical Res. 2006, 40, 847. 17015263 10.1080/10715760600730810

[247] M. Sibrian‐Vazquez , E. Hao , T. J. Jensen , M. G. H. Vicente , Bioconjugate Chem. 2006, 17, 928. 10.1021/bc060047v 16848399

[248] J.‐L. Qin , W.‐Y. Shen , Z.‐F. Chen , L.‐F. Zhao , Q.‐P. Qin , Y.‐C. Yu , H. Liang , Sci. Rep. 2017, 7, 46056. 28436418 10.1038/srep46056 PMC5402304

[249] M. Abudayyak , T. A. Gurkaynak , G. Özhan , Toxicol. Industrial Health 2017, 33, 646. 10.1177/0748233717706633 28595480

[250] G. Gupta , A. Gliga , J. Hedberg , A. Serra , D. Greco , I. Odnevall Wallinder , B. Fadeel , FASEB J. 2020, 34, 5262. 32060981 10.1096/fj.201902191RR

[251] N. Gal , S. Massalha , O. Samuelly‐Nafta , D. Weihs , Med. Eng. Phys. 2015, 37, 478. 25862332 10.1016/j.medengphy.2015.03.003

[252] C. Uboldi , T. Orsière , C. Darolles , V. Aloin , V. Tassistro , I. George , V. Malard , Part. Fibre Toxicol. 2015, 13, 5. 10.1186/s12989-016-0118-8 PMC4739324 26843362

[253] M. Savi , L. Bocchi , F. Cacciani , R. Vilella , A. Buschini , A. Perotti , S. Galati , S. Montalbano , S. Pinelli , C. Frati , E. Corradini , F. Quaini , R. Ruotolo , D. Stilli , M. Zaniboni , Part. Fibre Toxicol. 2021, 18, 1. 33407654 10.1186/s12989-020-00396-6 PMC7788732

[254] S. Chattopadhyay , S. Roy , Handbook of Oxidative Stress in Cancer, Mechanistic Aspects, Springer, Singapore 2021, pp 1–17.

[255] S. Chattopadhyay , S. P. Chakraborty , D. Laha , R. Baral , P. Pramanik , S. Roy , Cancer Nanotechnol. 2012, 3, 13. 26069493 10.1007/s12645-012-0026-z PMC4452042

[256] S. Chattopadhyay , S. K. Dash , T. Ghosh , D. Das , P. Pramanik , S. Roy , Cancer Nanotechnol. 2013, 4, 103. 26069506 10.1007/s12645-013-0042-7 PMC4451626

[257] N. Missaoui , A. Chrouda , H. Kahri , A. J. Gross , M. Rezaei Ardani , A. L. Pang , M. Ahmadipour , Sep. Purif. Technol. 2023, 316, 123755.

[258] L. Ding , X. Lin , Z. Lin , Y. Wu , X. Liu , J. Liu , M. Wu , X. Zhang , Y. Zeng , ACS Appl. Mater. Interfaces 2020, 12, 36906. 32706242 10.1021/acsami.0c09657

[259] M. Wu , Y. Yang , Adv. Mater. 2017, 29, 1606134. 10.1002/adma.201606134 28370555

[260] J. Yang , D. Dai , X. Zhang , L. Teng , L. Ma , Y.‐W. Yang , Theranostics 2023, 13, 295. 36593957 10.7150/thno.80687 PMC9800740

[261] M. Ibrahim , R. Sabouni , G. Husseini , Curr. Med. Chem. 2017, 24, 193. 27686655 10.2174/0929867323666160926151216

[262] L. D. Field , J. B. Delehanty , Y. Chen , I. L. Medintz , Accounts Chem. Res. 2015, 48, 1380. 10.1021/ar500449v 25853734

[263] A. A. Khan , K. S. Allemailem , A. Almatroudi , S. A. Almatroodi , M. A. Alsahli , A. H. Rahmani , J. Drug Deliv. Sci. Technol. 2021, 61, 102315.

[264] J. Yoo , C. Park , G. Yi , D. Lee , H. Koo , Cancers 2019, 11, 640. 31072061 10.3390/cancers11050640 PMC6562917

[265] J. Yang , A. Griffin , Z. Qiang , J. Ren , Signal Transduction Targeted Ther. 2022, 7, 379. 10.1038/s41392-022-01243-0 PMC9675787 36402753

[266] N. Zhang , G. Xiong , Z. Liu , Front. Bioeng. Biotechnol. 2022, 10. 10.3389/fbioe.2022.1001572 PMC9822575 36619393

[267] Z. Yuan , R. Yan , Z. Fu , T. Wu , C. Ren , Sci. Total Environ. 2024, 927, 172240. 38582114 10.1016/j.scitotenv.2024.172240

[268] J. M. Llobet , J. L. domingo , J. Corbella , Arch. Toxicol. 1986, 58, 278. 3087329 10.1007/BF00297121

[269] M. Moravcová , Z. Lomozová , R. Kučera , P. Mladěnka , RSC Adv. 2023, 13, 29242. 37809024 10.1039/d3ra02735j PMC10551802

[270] C. Sgarlata , V. Oliveri , J. Spencer , Eur. J. Inorg. Chem. 2015, 2015, 5886.

[271] Y. Cho , A. Mirzapour‐Kouhdasht , H. Yun , J. H. Park , H. J. Min , C. W. Lee , Int. J. Mol. Sci. 2022, 23, 719. 35054904 10.3390/ijms23020719 PMC8775498

[272] P. Chaudhary , P. Janmeda , A. O. Docea , B. Yeskaliyeva , A. F. Abdull Razis , B. Modu , D. Calina , J. Sharifi‐Rad , Fron. Chem. 2023, 11, 2023. 10.3389/fchem.2023.1158198 PMC10206224 37234200

[273] Y.‐K. Liu , H.‐W. Yang , M.‐H. Wang , W. Wang , F. Liu , H.‐L. Yang , Orthop. Surg. 2016, 8, 496. 28032714 10.1111/os.12298 PMC6584440

[274] R. D'Ambrosi , N. Ursino , Arthroplasty Today 2020, 6, 149. 32346587 10.1016/j.artd.2020.03.010 PMC7183002

[275] J. A. G. Crispo , D. R. Ansell , M. Piche , J. K. Eibl , N. Khaper , G. M. Ross , T. C. Tai , Can. J. Physiol. Pharmacol. 2010, 88, 429. 20555411 10.1139/y09-137

[276] J. Chen , C. Chen , N. Wang , C. Wang , Z. Gong , J. Du , H. Lai , X. Lin , W. Wang , X. Chang , M. Aschner , Z. Guo , S. Wu , H. Li , F. Zheng , NeuroToxicology 2023, 95, 155. 36716931 10.1016/j.neuro.2023.01.010

[277] M. Vasák , J. H. Kägi , Proc. Natl. Acad. Sci. USA 1981, 78, 6709. 6273885 10.1073/pnas.78.11.6709 PMC349119

[278] A. Cascajosa‐Lira , A. I. Prieto , S. Pichardo , A. Jos , A. M. Cameán , Phytomedicine 2024, 130, 155731. 38824824 10.1016/j.phymed.2024.155731

[279] C. Bresson , C. Lamouroux , C. Sandre , M. Tabarant , N. Gault , J. L. Poncy , J. L. Lefaix , C. Den Auwer , R. Spezia , M.‐P. Gaigeot , E. Ansoborlo , S. Mounicou , A. Fraysse , G. Deves , T. Bacquart , H. Seznec , T. Pouthier , P. Moretto , R. Ortega , R. Lobinski , C. Moulin , Biochimie 2006, 88, 1619. 17007991 10.1016/j.biochi.2006.09.003

[280] H. Salehi , I. Calas‐Bennasar , J.‐C. Durand , E. Middendorp , J. Valcarcel , C. Larroque , K. Nagy , K. Turzó K , I. Dekany , F. J. G. Cuisinier , J. Raman Spectrosc. 2014, 45, 807.

[281] H. Salehi , L. Derely , A.‐G. Vegh , J.‐C. Durand , C. Gergely , C. Larroque , M.‐A. Fauroux , F. J. G. Cuisinier , Appl. Phys. Lett. 2013, 102, 113701.

[282] H. Salehi , S. Al‐Arag , E. Middendorp , C. Gergley , F. Cuisinier , SPIE Proc. 2017, 10068, 1006805.

[283] E. Rauwel , S. Al‐Arag , H. Salehi , C. O. Amorim , F. Cuisinier , M. Guha , M. S. Rosario , P. Rauwel , Int. J. Nanomed. 2020, 15, 7051. 10.2147/IJN.S258060 PMC7522600 33061367

[284] B. R. Stockwell , Cell 2022, 185, 2401. 35803244 10.1016/j.cell.2022.06.003 PMC9273022

[285] D. Tang , X. Chen , R. Kang , G. Kroemer , Cell Res. 2020, 31, 107. 33268902 10.1038/s41422-020-00441-1 PMC8026611

[286] X. Jiang , B. R. Stockwell , M. Conrad , Nat. Rev. Mol. Cell Biol. 2021, 22, 266. 33495651 10.1038/s41580-020-00324-8 PMC8142022

[287] Q.‐M. Lou , F.‐F. Lai , J.‐W. Li , K.‐J. Mao , H.‐T. Wan , Y. He , Apoptosis 2024, 29, 981. 38824478 10.1007/s10495-024-01983-0

[288] S. J. Dixon , K. M. Lemberg , M. R. Lamprecht , R. Skouta , E. M. Zaitsev , C. E. Gleason , D. N. Patel , A. J. Bauer , A. M. Cantley , W. S. Yang , B. Morrison , B. R. Stockwell , Cell 2012, 149, 1060. 22632970 10.1016/j.cell.2012.03.042 PMC3367386

[289] J. Xie , Y. Yang , Y. Gao , J. He , Mol. Cancer 2023, 22, 46. 36882769 10.1186/s12943-023-01732-y PMC9990368

[290] L. Zhang , R. Deng , R. Guo , Y. Jiang , Y. Guan , C. Chen , W. Zhao , G. Huang , R. Guo , L. Liu , H. Du , D. Tang , Front. Mol. Biosci. 2024, 11, 1460987. 39297074 10.3389/fmolb.2024.1460987 PMC11408227

[291] D. Kong , F. Yan , Z. Han , J. Xu , X. Guo , L. Chen , RSC Adv. 2016, 6, 67481.

[292] L. Zhao , H. Li , H. Liu , M. Liu , N. Huang , Z. He , Y. Li , Y. Chen , L. Ding , Anal. Bioanal. Chem. 2019, 411, 2373. 30877345 10.1007/s00216-019-01678-5

[293] X. Zhao , L. Wang , Q. Liu , M. Chen , X. Chen , Microchem. J. 2021, 163, 105888.

[294] H. Y. Au‐Yeung , E. J. New , C. J. Chang , Chem. Commun. 2012, 48, 5268. 10.1039/c2cc31681a 22531796

[295] X. Fang , H. Wang , D. Han , E. Xie , X. Yang , J. Wei , S. Gu , F. Gao , N. Zhu , X. Yin , Q. Cheng , P. Zhang , W. Dai , J. Chen , F. Yang , H.‐T. Yang , A. Linkermann , W. Gu , J. Min , F. Wang , Proc. Natl. Acad. Sci. USA 2019, 116, 2672. 30692261 10.1073/pnas.1821022116 PMC6377499

[296] Q. Tan , D. Wu , Y. Lin , H. Ai , J. Xu , H. Zhou , Q. Gu , Bioorg. Chem. 2024, 146, 107261. 38460336 10.1016/j.bioorg.2024.107261

[297] J. Wang , L. K. Y. Chan , T. Zhang , J. Li , J. Liu , T. S. Lau , C. C. Wang , Cancer Treatment Modalities, An Interdisciplinary Approach, Springer International Publishing, Cham 2025, pp 295–331.

[298] B. Obied , G. Saar , S. Richard , Y. Rotenstreich , I. Sher , A. Zahavi , N. Goldenberg‐Cohen , Methods Protoc. 2025, 8, 1. 39846687 10.3390/mps8010001 PMC11755644

[299] V. Venkatraman , M. K. Wong , C. Shalita , B. Parente , S. P. Lad , Cureus 2020, 12, 12368. 10.7759/cureus.12368 PMC7842236 33527049

[300] L. Horev‐Azaria , C. J. Kirkpatrick , R. Korenstein , P. N. Marche , O. Maimon , J. Ponti , R. Romano , F. Rossi , U. Golla‐Schindler , D. Sommer , C. Uboldi , R. E. Unger , C. Villiers , Toxicol. Sci. 2011, 122, 489. 21602188 10.1093/toxsci/kfr124

[301] Q.‐Y. Zhou , C. Ren , J.‐Y. Li , L. Wang , Y. Duan , R.‐Q. Yao , Y.‐P. Tian , Y.‐M. Yao , Cell Death Dis. 2024, 15, 299. 38678018 10.1038/s41419-024-06691-w PMC11055915

[302] G. Eskander , S. G. Abdelhamid , S. A. Wahdan , S. M. Radwan , Cell Death Discovery 2025, 11, 56. 39929794 10.1038/s41420-025-02328-9 PMC11811070

[303] Y. E. Liu , S. Lu , L.‐L. Wu , L. Yang , L. Yang , J. Wang , Cell Death Dis. 2023, 14, 519. 37580393 10.1038/s41419-023-06045-y PMC10425449

[304] P. Malaviya , J. Kumar , R. A. Kowluru , Free Radical Biol. Med. 2024, 225, 821. 39433112 10.1016/j.freeradbiomed.2024.10.296 PMC11624098

[305] M. E. Aliaga , C. López‐Alarcón , G. Barriga , C. Olea‐Azar , H. Speisky , J. Inorg. Biochem. 2010, 104, 1084. 20638134 10.1016/j.jinorgbio.2010.06.006

[306] H. Speisky , M. Gómez , F. Burgos‐Bravo , C. López‐Alarcón , C. Jullian , C. Olea‐Azar , M. E. Aliaga , Bioorg. Med. Chem. 2009, 17, 1803. 19230679 10.1016/j.bmc.2009.01.069

[307] J. Liu , H. Tang , F. Chen , C. Li , Y. Xie , R. Kang , D. Tang , Sci. Rep. 2024, 14, 29579. 39609608 10.1038/s41598-024-81317-x PMC11605005

[308] J. Du , Z. Huang , Y. Li , X. Ren , C. Zhou , R. Liu , P. Zhang , G. Lei , J. Lyu , J. Li , G. Tan , Free Radical Biol. Med. 2023, 204, 359. 37225108 10.1016/j.freeradbiomed.2023.05.017

[309] I. Stejerean‐Todoran , C. S. Gibhardt , I. Bogeski , Cell Calcium 2024, 124, 102966. 39504596 10.1016/j.ceca.2024.102966

[310] W. Xu , A. Suo , A. J. M. Aldai , Y. Wang , J. Fan , Y. Xia , J. Xu , Z. Chen , H. Zhao , M. Zhang , J. Qian , ACS Nano 2024, 18, 30053. 39412236 10.1021/acsnano.4c11455

[311] J. C. Rutherford , A. J. Bird , Eukaryotic Cell 2004, 3, 1. 14871932 10.1128/EC.3.1.1-13.2004 PMC329510

[312] U. Lindert , M. Cramer , M. Meuli , O. Georgiev , W. Schaffner , Mol. Cell. Biol. 2009, 29, 6283. 19797083 10.1128/MCB.00847-09 PMC2786702

[313] G. Veeckmans , E. Van San , T. Vanden Berghe , FEBS J. 2023, 291, 2767. 37935445 10.1111/febs.16993

[314] T. A. Agbor , Z. Demma , R. J. Mrsny , A. Castillo , E. J. Boll , B. A. Mccormick , Cell. Microbiol. 2014, 16, 1339. 24617613 10.1111/cmi.12290 PMC4146641

[315] R. Riyazuddin , R. Gupta , Front. Plant Sci. 2021, 12, 680709. 34262583 10.3389/fpls.2021.680709 PMC8273338

[316] A. M. Distéfano , M. V. Martin , J. P. Córdoba , A. M. Bellido , S. D'Ippólito , S. L. Colman , D. Soto , J. A. Roldán , C. G. Bartoli , E. J. Zabaleta , D. F. Fiol , B. R. Stockwell , S. J. Dixon , G. C. Pagnussat , J. Cell Biol. 2017, 216, 463. 28100685 10.1083/jcb.201605110 PMC5294777

[317] J. Salguero‐Linares , N. S. Coll , PLoS Pathog. 2023, 19, 1011253. 10.1371/journal.ppat.1011253 PMC10079105 37023043

[318] Q. Shen , N. I. Naqvi , PLoS Pathog. 2021, 17, 1009298. 10.1371/journal.ppat.1009298 PMC7932112 33662044

[319] E. Chi Fru , N. P. Rodríguez , C. A. Partin , S. V. Lalonde , P. Andersson , D. J. Weiss , A. El Albani , I. Rodushkin , K. O. Konhauser , Proc. Natl. Acad. Sci. USA 2016, 113, 4941. 27091980 10.1073/pnas.1523544113 PMC4983842

[320] D. E. Canfield , L. Ngombi‐Pemba , E. U. Hammarlund , S. Bengtson , M. Chaussidon , F. Gauthier‐Lafaye , A. Meunier , A. Riboulleau , C. Rollion‐Bard , O. Rouxel , D. Asael , A.‐C. Pierson‐Wickmann , A. El Albani , Proc. Natl. Acad. Sci. USA 2013, 110, 16736. 24082125 10.1073/pnas.1315570110 PMC3801071

[321] S. Passey , S. Pellegrin , H. Mellor , Current Protoc. Cell Biol. 2007, 37. 10.1002/0471143030.cb0417s37 18228517

[322] L. Schermelleh , A. Ferrand , T. Huser , C. Eggeling , M. Sauer , O. Biehlmaier , G. P. C. Drummen , Nat. Cell Biol. 2019, 21, 72. 30602772 10.1038/s41556-018-0251-8

[323] K. P. Carter , A. M. Young , A. E. Palmer , Chem. Rev. 2014, 114, 4564. 24588137 10.1021/cr400546e PMC4096685

[324] J. S Becker , M. Zoriy , A. Matusch , B. Wu , D. Salber , C. Palm , J. S Becker , Mass Spectrometry Rev. 2009, 29, 156. 10.1002/mas.20239 19557838

[325] L. Yang , R. Mcrae , M. M. Henary , R. Patel , B. Lai , S. Vogt , C. J. Fahrni , Proc. Natl. Acad. Sci. USA 2005, 102, 11179. 16061820 10.1073/pnas.0406547102 PMC1183533

[326] K. G. Slepchenko , S. Chen , K. L. Corbin , R. A. Colvin , C. S. Nunemaker , Metallomics 2023, 15, mfad006. 36737500 10.1093/mtomcs/mfad006 PMC9933206

[327] A. Iglesias‐Juez , G. L. Chiarello , G. S. Patience , M. O Guerrero‐Pérez , Canadian J. Chem. Eng. 2021, 100, 3.

[328] A. Jasniewski , Y. Hu , M. W. Ribbe , Metalloproteins, Springer, New York, 2019, pp 197–211. 10.1007/978-1-4939-8864-8_13 30317483

[329] M. Gutscher , A.‐L. Pauleau , L. Marty , T. Brach , G. H. Wabnitz , Y. Samstag , A. J. Meyer , T. P. Dick , Nat. Methods 2008, 5, 553. 18469822 10.1038/nmeth.1212

[330] N. Soleja , M. Mohsin , Sensors Diagn. 2024, 3, 1714.

[331] W.‐C. Yang , S.‐Y. Li , S. Ni , G. Liu , Aggregate 2023, 5, 460.

[332] M. Mann , O. N. Jensen , Nat. Biotechnol. 2003, 21, 255. 12610572 10.1038/nbt0303-255

[333] G. J. Patti , O. Yanes , G. Siuzdak , Nat. Rev. Mol. Cell Biol. 2012, 13, 263. 22436749 10.1038/nrm3314 PMC3682684

[334] P. D. Hsu , E. S. Lander , F. Zhang , Cell 2014, 157, 1262. 24906146 10.1016/j.cell.2014.05.010 PMC4343198

[335] S. G. Aller , E. T. Eng , C. J. De Feo , V. M. Unger , J. Biol. Chem. 2004, 279, 53435. 15385536 10.1074/jbc.M409421200 PMC1201109

[336] L. Przybyla , L. A. Gilbert , Nat. Rev. Genet. 2021, 23, 89. 34545248 10.1038/s41576-021-00409-w

[337] S. M. Lamperis , K. M. Mcmahon , A. E. Calvert , J. S. Rink , K. Vasan , M. R. Pandkar , E. U. Crentsil , Z. R. Chalmers , N. R. Mcdonald , C. J. Kosmala , M. G. Bonini , D. Matei , L. I. Gordon , N. S. Chandel , C. S Thaxton , Proc. Natl. Acad. Sci. USA 2025, 122, 2502876122. 10.1073/pnas.2502876122 PMC12146712 40445760

[338] R. Tian , A. Abarientos , J. Hong , S. H. Hashemi , R. Yan , N. Dräger , K. Leng , M. A. Nalls , A. B. Singleton , K. Xu , F. Faghri , M. Kampmann , Nat. Neurosci. 2021, 24, 1020. 34031600 10.1038/s41593-021-00862-0 PMC8254803

[339] E. Ng , P. M Lind , C. Lindgren , E. Ingelsson , A. Mahajan , A. Morris , L. Lind , Hum. Mol. Genet. 2015, 24, 4739. 26025379 10.1093/hmg/ddv190 PMC4512629

[340] N. Kim , D. Filipovic , S. Bhattacharya , S. Cuddapah , Environ. Int. 2024, 193, 109084. 39437622 10.1016/j.envint.2024.109084

[341] F. Tang , C. Barbacioru , Y. Wang , E. Nordman , C. Lee , N. Xu , X. Wang , J. Bodeau , B. B. Tuch , A. Siddiqui , K. Lao , M. A. Surani , Nat. Methods 2009, 6, 377. 19349980 10.1038/nmeth.1315

[342] D. P. Bartel , Cell 2004, 116, 281. 14744438 10.1016/s0092-8674(04)00045-5

[343] S. Wang , R. Ma , Z. Mei , Y. Hou , MedMat 2024, 1, 6.

[344] X. Liang , M. Chen , P. Bhattarai , S. Hameed , Y. Tang , Z. Dai , ACS Nano 2021, 15, 20164. 34898184 10.1021/acsnano.1c08108

[345] Y. Xu , S.‐Y. Liu , L. Zeng , H. Ma , Y. Zhang , H. Yang , Y. Liu , S. Fang , J. Zhao , Y. Xu , C. R. Ashby , Y. He , Z. Dai , Y. Pan , Adv. Mater. 2022, 34, 2204733. 10.1002/adma.202204733 36054475

[346] F. Jiang , Y. Zhao , C. Yang , Z. Cheng , M. Liu , B. Xing , B. Ding , P. '. Ma , J. Lin , Dalton Trans. 2022, 51, 2798. 35084419 10.1039/d1dt04120g

[347] K. Li , L. Wu , H. Wang , Z. Fu , J. Gao , X. Liu , Y. Fan , X. Qin , D. Ni , J. Wang , D. Xie , J. Nanobiotechnol. 2024, 22, 546. 10.1186/s12951-024-02828-3 PMC11378619 39237931

[348] R. Cheng , S. Wang , Drug Deliv. Transl. Res. 2024, 14, 3032. 38615157 10.1007/s13346-024-01591-0 PMC11445310

[349] Y. Li , X. Li , L. Wei , J.‐F. Ye , Front. Immunol. 2024, 15, 1451989. 39483479 10.3389/fimmu.2024.1451989 PMC11524880

[350] J. S. Suk , Q. Xu , N. Kim , J. Hanes , L. M. Ensign , Adv. Drug Delivery Rev. 2016, 99, 28. 10.1016/j.addr.2015.09.012 PMC4798869 26456916

[351] C. Saraiva , C. Praça , R. Ferreira , T. Santos , L. Ferreira , L. Bernardino , J. Controlled Release 2016, 235, 34. 10.1016/j.jconrel.2016.05.044 27208862

[352] T. R. Kyriakides , A. Raj , T. H. Tseng , H. Xiao , R. Nguyen , F. S. Mohammed , S. Halder , M. Xu , M. J. Wu , S. Bao , W. C. Sheu , Biomed. Mater. 2021, 16, 042005. 10.1088/1748-605X/abe5fa PMC8357854 33578402

[353] G. Dogheim , S. Chinnam , M. T. Amralla , Curr. Drug Delivery 2024, 21, 837. 10.2174/0115672018292331240404070236 38698743

[354] T. Jiang , K. M. Gonzalez , L. E. Cordova , J. Lu , Expert Opin. Drug Deliv. 2023, 20, 523. 37017558 10.1080/17425247.2023.2200246 PMC10164135

[355] S. W. L. Lee , C. Paoletti , M. Campisi , T. Osaki , G. Adriani , R. D. Kamm , C. Mattu , V. Chiono , J. Controlled Release 2019, 313, 80. 10.1016/j.jconrel.2019.10.007 PMC6900258 31622695

[356] R. A. Foley , P. G. Ayoub , V. Sinha , et al., bioRxiv 2025.

[357] J. Szpunar , Analyst 2005, 130, 442. 15776152 10.1039/b418265k

[358] R. Ortega , P. Cloetens , G. Devès , A. Carmona , S. Bohic , PLoS One 2007, 2, 925. 10.1371/journal.pone.0000925 PMC1976597 17895967

[359] C. D. Gutierrez Reyes , G. Alejo‐Jacuinde , B. Perez Sanchez , J. Chavez Reyes , S. Onigbinde , D. Mogut , I. Hernández‐Jasso , D. Calderón‐Vallejo , J. L Quintanar , Y. Mechref , Current Issues Mol. Biol. 2024, 46, 5777. 10.3390/cimb46060345 PMC11202207 38921016

[360] I. Subramanian , S. Verma , S. Kumar , A. Jere , K. Anamika , Bioinform. Biol. Insights 2020, 14, 117793221989905. 10.1177/1177932219899051 PMC7003173 32076369

[361] J. Yan , S. L. Risacher , L. Shen , A. J. Saykin , Brief. Bioinform. 2017, 19, 1370. 10.1093/bib/bbx066 PMC6454489 28679163

[362] P. S. Reel , S. Reel , E. Pearson , E. Trucco , E. Jefferson , Biotechnol. Adv. 2021, 49, 107739. 33794304 10.1016/j.biotechadv.2021.107739

[363] P. H. G Sanches , N. C. De Melo , A. M. Porcari , L. M. De Carvalho , Biology 2024, 13, 848. 39596803 10.3390/biology13110848 PMC11592251

[364] A. Yetgin , Quantitative Biol. 2025, 13, 70002. 10.1002/qub2.70002 PMC12806145 41675959

[365] J. D. Ewald , G. Zhou , Y. Lu , J. Kolic , C. Ellis , J. D. Johnson , P. E. Macdonald , J. Xia , Nat. Protoc. 2024, 19, 1467. 38355833 10.1038/s41596-023-00950-4

[366] G. Zhou , Z. Pang , Y. Lu , J. Ewald , J. Xia , Nucleic Acids Res. 2022, 50, W527. 35639733 10.1093/nar/gkac376 PMC9252810

[367] M. Arbatskiy , D. Balandin , I. Akberdin , A. Churov , Int. J. Mol. Sci. 2024, 25, 11782. 39519341 10.3390/ijms252111782 PMC11546516

[368] G. Du , Q. Fang , J. M. J. Den Toonder , Anal. Chim. Acta 2016, 903, 36. 26709297 10.1016/j.aca.2015.11.023

[369] P. De Stefano , E. Bianchi , G. Dubini , Biomicrofluidics 2022, 16, 031501. 35646223 10.1063/5.0087294 PMC9142169

[370] J. Beardmore , C. Exley , J. Inorg. Biochem. 2009, 103, 205. 19013648 10.1016/j.jinorgbio.2008.10.003

[371] S. Ekins , J. Mestres , B. Testa , Br. J. Pharmacol. 2007, 152, 9. 17549047 10.1038/sj.bjp.0707305 PMC1978274

[372] F. Yang , L. Jia , H.‐C. Zhou , J.‐N. Huang , M.‐Y. Hou , F.‐T. Liu , N. Prabhu , Z.‐J. Li , C.‐B. Yang , C. Zou , P. Nordlund , J.‐G. Wang , L.‐Y. Dai , Acta Pharmacol. Sin. 2023, 45, 391. 37803139 10.1038/s41401-023-01167-7 PMC10789809

[373] V. P. Sharma , D. Entenberg , J. Condeelis , Adhesion Protein Protocols, Humana Press, Totowa, NJ 2013, pp 343–357.

[374] C. Xu , M. Nedergaard , D. J. Fowell , P. Friedl , N. Ji , Cell 2024, 187, 4458. 39178829 10.1016/j.cell.2024.07.036 PMC11373887

[375] Y. F. Dufrêne , T. Ando , R. Garcia , D. Alsteens , D. Martinez‐Martin , A. Engel , C. Gerber , D. J. Müller , Nat. Nanotechnol. 2017, 12, 295. 28383040 10.1038/nnano.2017.45

[376] S. Shimma , Mass Spectrometry 2022, 11, A0102. 35291501 10.5702/massspectrometry.A0102 PMC8900255

[377] X. Zhou , S. Mehta , J. Zhang , Trends Biochem. Sci. 2020, 45, 889. 32660810 10.1016/j.tibs.2020.06.001 PMC7502535

[378] A. Shariff , J. Kangas , L. P. Coelho , S. Quinn , R. F. Murphy , SLAS Discov. 2010, 15, 726. 10.1177/1087057110370894 20488979

[379] Y. Zhao , O. N. Jensen , Proteomics 2009, 9, 4632. 19743430 10.1002/pmic.200900398 PMC2892724

[380] J. Taylor , S. Woodcock , SLAS Discov. 2015, 20, 1040.

[381] M. Mehrmohamadi , M. H. Sepehri , N. Nazer , M. R. Norouzi , Front. Cell Dev. Biol. 2021, 9, 2021. 10.3389/fcell.2021.714687 PMC8340004 34368164

[382] A. Haque , J. Engel , S. A. Teichmann , T. Lönnberg , Genome Med. 2017, 9, 75. 28821273 10.1186/s13073-017-0467-4 PMC5561556

[383] Q. Li , X. Zhang , R. Ke , Front. Genet. 2022, 13, 2022. 10.3389/fgene.2022.906158 PMC9309247 35899203

[384] A. Bhusal , S. Yogeshwaran , H. Goodarzi Hosseinabadi , B. Cecen , A. K. Miri , Biomed. Mater. Devices 2024, 3, 93.

[385] C.‐Y. Hsu , A. M. Rheima , M. M. Kadhim , N. N. Ahmed , S. H. Mohammed , F. H. Abbas , Z. T. Abed , Z. M. Mahdi , Z. S. Abbas , S. K. Hachim , F. K. Ali , Z. H. Mahmoud , E. Kianfar , South Afr. J. Chem. Eng. 2023, 46, 233.

[386] J. K. Patra , G. Das , L. F. Fraceto , E. V. R. Campos , M. D. P. Rodriguez‐Torres , L. S. Acosta‐Torres , L. A. Diaz‐Torres , R. Grillo , M. K. Swamy , S. Sharma , S. Habtemariam , H.‐S. Shin , J. Nanobiotechnol. 2018, 16, 71. 10.1186/s12951-018-0392-8 PMC6145203 30231877

[387] J. Soukar , N. A. Peppas , A. K. Gaharwar , Adv. Sci. 2025, 12, 2411720. 10.1002/advs.202411720 PMC11831507 39806939

[388] L. Xu , M. Peng , T. Gao , D. Wang , X. Lian , H. Sun , J. Shi , Y. Wang , P. Wang , Adv. Sci. 2023, 11, 2306203. 10.1002/advs.202306203 PMC10870045 38063781

[389] B. Lobato‐Delgado , B. Priego‐Torres , D. Sanchez‐Morillo , Cancers 2022, 14, 3215. 35804988 10.3390/cancers14133215 PMC9265023

[390] B. L. Puniya , J. Mol. Biol. 2025, 437, 169181. 40316010 10.1016/j.jmb.2025.169181

[391] F. R. Pinu , D. J. Beale , A. M. Paten , K. Kouremenos , S. Swarup , H. J. Schirra , D. Wishart , Metabolites 2019, 9, 76. 31003499 10.3390/metabo9040076 PMC6523452

[392] Q. T. H. Shubhra , Med. Rev. 2023, 3, 444. 10.1515/mr-2023-0029 PMC10811351 38283254

[393] X. Tian , L. Ruan , S. Zhou , L. Wu , J. Cao , X. Qi , X. Zhang , S. Shen , ACS Appl. Bio Mater. 2022, 5, 1692. 10.1021/acsabm.2c00068 35297253

[394] R. Fernández‐Acosta , C. Iriarte‐Mesa , D. Alvarez‐Alminaque , B. Hassannia , B. Wiernicki , A. M. Díaz‐García , P. Vandenabeele , T. Vanden Berghe , G. L. Pardo Andreu , Molecules 2022, 27, 3970. 35807217 10.3390/molecules27133970 PMC9268471

[395] H. Zafar , F. Raza , S. Ma , Y. Wei , J. Zhang , Q. Shen , Biomater. Sci. 2021, 9, 5092. 34160488 10.1039/d1bm00721a

[396] S. Baldari , G. Di Rocco , G. Toietta , Int. J. Mol. Sci. 2020, 21, 1069. 32041110 10.3390/ijms21031069 PMC7037088

[397] K. Deh , M. Zaman , Y. Vedvyas , Z. Liu , K. M. Gillen , P. O’ Malley , D. Bedretdinova , T. Nguyen , R. Lee , P. Spincemaille , J. Kim , Y. Wang , M. M. Jin , Sci. Rep. 2020, 10, 1171. 31980695 10.1038/s41598-020-58219-9 PMC6981186

[398] Y. Wang , T. Liu , Magnetic Resonance in Medicine 2014, 73, 82. 25044035 10.1002/mrm.25358 PMC4297605

[399] Y. Shibata , H. Yasui , K. Higashikawa , Y. Kuge , Biochem. Biophys. Rep. 2021, 26, 100957. 33681481 10.1016/j.bbrep.2021.100957 PMC7910409

[400] N. Zhao , Y. Huang , Y.‐H. Wang , R. K. Muir , Y.‐C. Chen , J. Wei , N. Hooshdaran , P. Viswanath , Y. Seo , D. Ruggero , A. R. Renslo , M. J. Evans , J. Nucl. Med. 2020, 62, 949. 33246980 10.2967/jnumed.120.252460 PMC8882888

[401] A. Hoehne , M. L. James , I. S. Alam , J. A. Ronald , B. Schneider , A. D'Souza , T. H. Witney , L. E. Andrews , H. C. Cropper , D. Behera , G. Gowrishankar , Z. Ding , T. Wyss‐Coray , F. T. Chin , S. Biswal , S. S. Gambhir , J. Neuroinflamm. 2018, 15, 55. 10.1186/s12974-018-1080-1 PMC5822551 29471880

[402] S. Y. Park , S. J. Na , M. Kumar , C. Mosci , M. Wardak , N. Koglin , S. Bullich , A. Mueller , M. Berndt , A. W. Stephens , Y. M. Cho , H. Ahn , S. Y. Chae , H. O. Kim , D. H. Moon , S. S. Gambhir , E. S. Mittra , Clin. Cancer Res. 2020, 26, 5380. 32694158 10.1158/1078-0432.CCR-20-0644

[403] F. Zeng , S. Nijiati , Y. Liu , et al., Sci. Adv. 2023, 9, add8539. 10.1126/sciadv.add8539 PMC9995079 36888714

[404] J. T. Jørgensen , M. Persson , J. Madsen , A. Kjær , Nucl. Med. Biol. 2013, 40, 345. 23394821 10.1016/j.nucmedbio.2013.01.002

[405] G. Capriotti , A. Piccardo , E. Giovannelli , A. Signore , J. Clin. Med. 2022, 12, 223. 36615024 10.3390/jcm12010223 PMC9821557

[406] H. Xu , Z. Guo , M. Li , H. V. Chaves , V. D. P. T Pinto , G. C. Filho , M. Du , M. M. Bezerra , BIO Integr. 2024, 5, 1.

[407] H. F. Zhang , K. Maslov , G. Stoica , L. V. Wang , Nat. Biotechnol. 2006, 24, 848. 16823374 10.1038/nbt1220

[408] Z. Wang , F. Yang , W. Zhang , K. Xiong , S. Yang , Fundamental Res. 2024, 4, 1314. 10.1016/j.fmre.2023.01.008 PMC11489505 39431136

[409] S. Wang , L. Zhang , J. Zhao , M. He , Y. Huang , S. Zhao , Sci. Adv. 2021, 7, abe3588. 10.1126/sciadv.abe3588 PMC7978424 33741594

[410] A. K. Bindra , D. Wang , Z. Zheng , D. Jana , W. Zhou , S. Yan , H. Wu , Y. Zheng , Y. Zhao , Biomaterials 2021, 279, 121188. 34678649 10.1016/j.biomaterials.2021.121188

